# Tumor-repopulating cells evade ferroptosis via PCK2-dependent phospholipid remodeling

**DOI:** 10.1038/s41589-024-01612-6

**Published:** 2024-05-08

**Authors:** Zhe Li, Zhi-min Xu, Wei-peng Chen, Xiao-jing Du, Chun-xian Ou, Zi-kang Luo, Rong Wang, Chu-qing Zhang, Chao-dong Ge, Meng Han, Fudi Wang, Rong-Rong He, Wan-yang Sun, Jun Ma, Xiao-yu Liang, Zhuo-wei Liu

**Affiliations:** 1 State Key Laboratory of Oncology in South China, Collaborative Innovation Center of Cancer Medicine, Sun Yat-sen University Cancer Center, Guangzhou, China; 2 Guangdong Key Laboratory of Nasopharyngeal Carcinoma Diagnosis and Therapy, Sun Yat-sen University Cancer Center, Guangzhou, China; 3 Department of Radiation Oncology, Sun Yat-sen University Cancer Center, Guangzhou, China; 4 Guangdong Engineering Research Center of Chinese Medicine & Disease Susceptibility, State Key Laboratory of Bioactive Molecules and Druggability Assessment, Jinan University, Guangzhou, China; 5 The Second Affiliated Hospital, School of Public Health, State Key Laboratory of Experimental Hematology, Zhejiang University School of Medicine, Hangzhou, China; 6 The First Affiliated Hospital, Basic Medical Sciences, School of Public Health, Hengyang Medical School, University of South China, Hengyang, China; 7 Protein Research Technology Center Protein Chemistry and Omics Platform, Tsinghua University, Beijing, China; 8 School of Pharmacy, Shenyang Pharmaceutical University, Shenyang, China; 9 Department of Urology, Sun Yat-sen University Cancer Center, Guangzhou, China

**Keywords:** Cancer therapy, Membrane lipids, Cell death, Post-translational modifications, Kinases

## Abstract

Whether stem-cell-like cancer cells avert ferroptosis to mediate therapy resistance remains unclear. In this study, using a soft fibrin gel culture system, we found that tumor-repopulating cells (TRCs) with stem-cell-like cancer cell characteristics resist chemotherapy and radiotherapy by decreasing ferroptosis sensitivity. Mechanistically, through quantitative mass spectrometry and lipidomic analysis, we determined that mitochondria metabolic kinase PCK2 phosphorylates and activates ACSL4 to drive ferroptosis-associated phospholipid remodeling. TRCs downregulate the PCK2 expression to confer themselves on a structural ferroptosis-resistant state. Notably, in addition to confirming the role of PCK2-pACSL4(T679) in multiple preclinical models, we discovered that higher PCK2 and pACSL4(T679) levels are correlated with better response to chemotherapy and radiotherapy as well as lower distant metastasis in nasopharyngeal carcinoma cohorts.

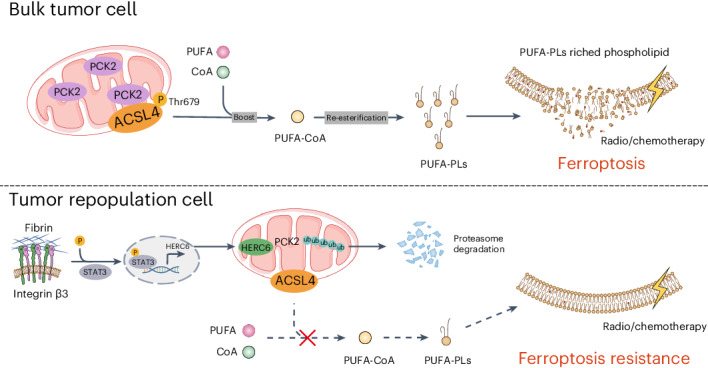

## Abstract (web summary)

Phosphorylation of ACSL4 by mitochondria-located metabolic kinase PCK2 is critical to regulating ferroptosis-associated phospholipid remodeling in tumor-repopulating cells that are resistant to chemotherapy and radiotherapy.

## Main

Ferroptosis is characterized by iron-dependent lipid peroxidation^1^. Accumulating evidence suggests that ferroptosis plays a critical role in tumor suppression. Tumor suppressor genes (for example, P53 (ref. ^2^)) or immune factors (for example, IFNγ (ref. ^3^)) are involved in enhancing or inducing ferroptosis, leading to the suppression of tumor progression. Importantly, chemotherapy^4^, radiotherapy^5^ and immunotherapy^6^ can induce ferroptosis. Stem-cell-like cancer cells (SCLCCs), which are self-renewing and highly tumorigenic subpopulations of cancer cells, can repopulate tumors and are resistant to multiple treatments, thus mediating tumor recurrence^7^. The resistance of SCLCCs toward various treatments is primarily mediated by avoiding programmed death processes to escape from predetermined death^7^. However, whether SCLCCs avert ferroptosis to mediate therapy resistance remains unclear. Specifically, in a neuroblastoma cell line SH-SY5Y-based differentiation model, precursor cells with higher stemness were resistant to ferroptosis as compared to differentiated progeny cells^8^; similarly, cardiomyocyte precursors also show resistance to ferroptosis compared to cardiomyocytes^8^. In contrast, tumor cells that highly express CD44 or undergo epithelial–mesenchymal transition, which are the characteristics of SCLCCs derived from certain tissues, such as breast, bladder or colon tissue, are more susceptible to ferroptosis^9^. Therefore, it is crucial to clarify the relationship between stem-like tumor cells and ferroptosis sensitivity and to explore the underlying mechanisms.

The degree of phospholipid (PL) unsaturation is a critical factor in determining the susceptibility of cells to ferroptosis. Acyl-CoA synthetase long chain family member 4 (ACSL4) catalyzes the production of polyunsaturated fatty acid (PUFA)-CoA^10^. Activated PUFA is further integrated into PLs catalyzed by membrane-bound *O*-acyltransferases, particularly lysophosphatidylcholine acyltransferase 3 (LPCAT3), which subsequently enters the cell membrane^11^. Furthermore, in eukaryotes, post-transcriptional modifications, such as phosphorylation of ACSL4 (ref. ^12^), are crucial for regulating enzymatic activity, and different kinases or modification sites may lead to opposite effects. Interestingly, a special class of kinases, such as phosphoglycerate kinase 1 (PGK1) (ref. ^13^) or pyruvate kinase M2 (PKM2) (ref. ^14^), which typically act as metabolic enzymes to mediate the phosphorylation of small-molecule metabolites, can also function as protein kinases, thereby creating strong crosstalk between metabolism and signal transduction^15^. Although it is thought that ferroptosis is regulated mainly by intracellular redox balance and metabolic flow shift^16^, whether this occurs through non-canonical functions of metabolic enzymes needs to be further elucidated.

In the present study, we used a biomechanical forces-based three-dimensional (3D) soft fibrin gel culture system to obtain tumor-repopulating cells (TRCs) and further explored whether the resistance of TRCs to chemotherapy and radiotherapy was related to ferroptosis sensitivity. Through quantitative mass spectrometry analysis, lipidomic/redox lipidomic analysis and gene editing, we revealed an unrecognized link between mitochondrial phosphoenolpyruvate carboxykinase 2 (PCK2) and ACSL4 T679 phosphorylation. PCK2 downregulation and ACSL4 inactivation facilitate TRCs on a structural ferroptosis-resistant state. More notably, we verified the effects of PCK2-pACSL4(T679) in in vivo tumor models and clarified its clinical relevance in nasopharyngeal carcinoma (NPC) chemotherapy and radiotherapy cohorts.

## Results

### TRCs resist chemoradiotherapy by evading ferroptosis

**Fig. 1:**
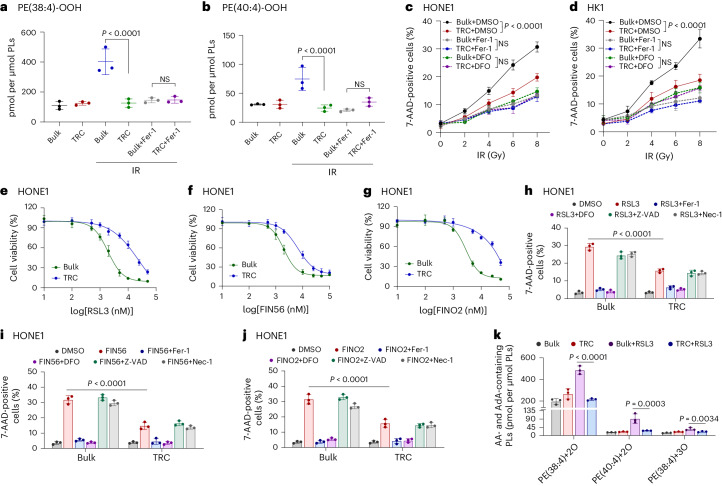
TRCs resist radiotherapy and chemotherapy by evading ferroptosis. **a**,**b**, HONE1 TRCs and bulk cells were treated with radiation (IR, 8 Gy) with or without Fer-1 (1 µM) for 60 h. Fer-1 was added 6 h before IR treatment. The content of ferroptotic cell death signal PE (38:4)-OOH (**a**) and PE (40:4)-OOH (**b**) was measured by LC–MS/MS. *P* = 0.000028, 0.9999996, 0.000344 and 0.474506. **c**, Percentage of dead cells in TRCs and bulk tumor cells from HONE1 cells treated with IR in the absence or presence of Fer-1 (1 μM) and DFO (20 μM). *P* = 0.000000000000101. **d**, Percentage of dead cells in TRCs and bulk tumor cells from HK1 cells treated with IR in the absence or presence of Fer-1 (2 μM) and DFO (20 μM). *P* = 0.000000000000019. **e**–**g**, Dose-dependent toxicity of ferroptosis inducers RSL3 (**e**), FIN56 (**f**) or FINO2 (**g**) induced cell death of TRCs and bulk tumor cells (HONE1). *n* = 3 replicates from one representative of three independent experiments. **h**–**j**, Percentage of dead cells in TRCs and bulk tumor cells from HONE1 cells treated with indicated inhibitors and RSL3 (15 μM) (**h**), FIN56 (10 μM) (**i**) or FINO2 (10 μM) (**j**). Fer-1, 1 μM; Z-VAD (Z-VAD-FMK), 10 μM and Nec-1 (necrostatin-1), 2 μM. *P* = 0.000000000042792, 0.000000000075223 and 0.000000002261459. **k**, Accumulation of RSL3-induced oxygenated PE that contains AA (C20:4) or AdA (C22:4) in TRCs and bulk tumor cells from HONE1 cells. *P* = 0.00000879. *n* = 3 replicates from one representative of three independent experiments. Data are shown as mean ± s.d.; one-way ANOVA (**a**,**b**,**k**) or two-way ANOVA (**c**,**d**,**h**–**j**). *n* = 3 independent experiments. NS, not significant. Source data

**Extended Data Fig. 1:**
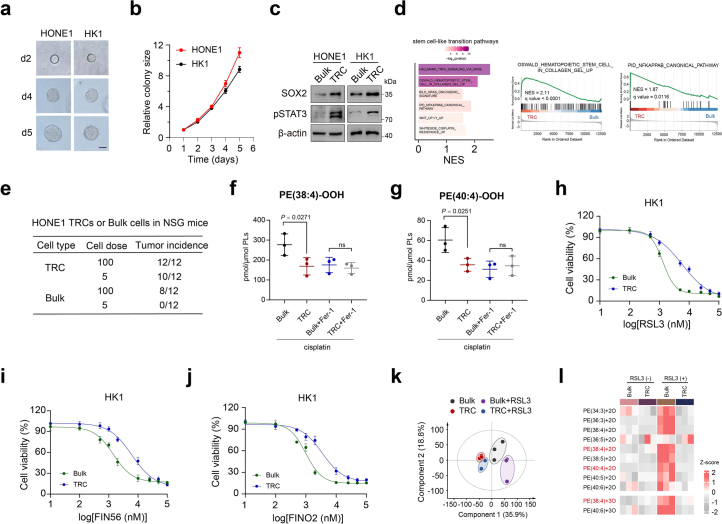
TRCs resist radiotherapy and chemotherapy via evading ferroptosis. **a-b**, HONE1 cells and HK1 cells were seeded in 3D fibrin gels for 5 days of culture. The colony size of the tumor spheroid was presented **(a)**. The colony sizes at different time points were quantified and the colony size on d1 was set to 1 **(b)**. Bar, 50 mm. n = 6. **c**, Immunoblots showing the expression of cancer stem cell markers (pSTAT3 and SOX2) in TRCs and bulk cells. **d**, RNA-seq was performed on HONE1 TRCs and bulk cells. The gene set enrichment analysis (GSEA) shows that multiple stem cell-like transition pathways were enriched in the TRCs group. Two-sided *P* values were calculated with Kolmogorov-Smirnov test and multiple-comparison adjusted q values were calculated using Benjamini-Hochberg (BH) method. **e**, The HONE1 TRCs and bulk cells (5 cells or 100 cells) were injected into the mammary fat pads of NSG mice. Eight weeks later, the tumor formation was recorded. n = 12. **f-g**, HONE1 TRCs and bulk cells were treated with cisplatin (Cis) with or without Fer-1. The content of ferroptotic cell death signal, PE (38:4)-OOH **(f)** and PE (40:4)-OOH **(g)** was measured. **h-j**, Dose-dependent toxicity of RSL3 **(h)**, FIN56 **(i)** or FINO2 **(j)** induced cell death of HK1 cells. **k**, Principal component analysis (PCA) of oxygenated phospholipids in HONE1 TRCs and bulk cells. Each point represents a sample. **l**, Heatmap showing the relative changes in RSL3-induced oxygenated PE and PC molecular species in HONE1 TRCs and bulk cells. Row z-scores were obtained from averaged values of the content of lipid species. Data are shown as mean ± SD (b, f-g), one-way ANOVA (f-g). n = 3 independent experiments. One of three experiments is shown (c). ns, not significant. Source data

Comprehensive chemoradiotherapy, instead of surgery, is the mainstay treatment for NPC^17,18^. Ferroptosis plays an important role in determining chemoradiotherapy efficacy, and SCLCCs mediate treatment resistance in multiple tumors^19^. Therefore, we explored whether SCLCCs mediate chemoradiotherapy resistance by regulating ferroptosis. Previously, we established a mechanics-based 3D soft fibrin gel culture system to select and amplify highly tumorigenic TRCs^20^. In this study, we further confirmed that the soft fibrin gel cultured NPC cell lines (HONE1 and HK1) exhibited SCLCC characteristics, as we previously observed^21^ (Extended Data Fig. 1a–e). Notably, five TRCs derived from the HONE1 cells could form palpable tumors in 10 out of 12 NSG mice within 8 weeks (Extended Data Fig. 1e). We then assessed ferroptosis through liquid chromatography–mass spectromety (LC–MS)-based redox lipidomic analysis^22^. Interestingly, TRCs derived from HONE1 cells showed markedly decreased levels of ferroptosis PL signals, typically di-oxygenated arachidonoyl and adrenoyl-phosphatidylethanolamine (PE) species^23^ (PE-(38:4)-OOH;PE-(40:4)-OOH) under the treatments of radiation or cisplatin. Notably, when lipophilic radical-trapping antioxidant ferrostatin-1 (Fer-1) was added, the differences of ferroptosis PL signals between bulk tumor cells and TRCs were abolished (Fig. 1a,b and Extended Data Fig. 1f,g). Consistently, under cisplatin or radiation treatment, TRCs derived from either HONE1 or HK1 cells showed less cell death compared to bulk tumor cells, whereas Fer-1 or iron chelator deferoxamine (DFO) treatment significantly decreased the cell death in bulk tumor cells to a similar level to that of TRCs (Fig. 1c,d and Supplementary Fig. 1a,b). To generalize our findings, we extended our studies to cell lines (A375/HCT116) derived from other tissues (melanoma/colon) and found that A375 and HCT116 TRCs also showed decreased cell death under cisplatin or radiation. In addition, the cell death discrepancy between TRCs and bulk tumor cells could also be diminished by adding Fer-1 or DFO (Supplementary Fig. 1c,d).

To further confirm that TRCs resisted ferroptosis, we evaluated the sensitivity of HONE1, HK1 and A375 cells toward multiple ferroptosis inducers based on different mechanisms. These three cell lines were found to be insensitive to intracellular cysteine deprivation (medium cystine starvation and erastin) but consistently showed high sensitivity to RSL3 (GPX4 inhibitor), FIN56 (GPX4 deprivation and squalene synthase agonist) and FINO2 (endoperoxide). In addition, HONE1 cells were insensitive to the recently discovered endogenous ferroptosis-inducing methods (IFNγ + arachidonic acid (AA))^6^ (Supplementary Fig. 1e). As expected, we found that TRCs derived from HONE1 or HK1 cells were highly tolerant to RSL3, FIN56 and FINO2 at multiple dosages compared to their bulk tumor cells (Fig. 1e–g and Extended Data Fig. 1h–j). To exclude the involvement of other forms of cell death, we treated indicated cells with corresponding inhibitors that specifically target necroptosis (necrostatin-1), apoptosis (Z-VAD-FMK) or ferroptosis (Fer-1/DFO) and found that only Fer-1 or DFO could rescue the cell death conferred by ferroptosis inducers. Notably, ferroptosis inhibitors almost abolished the differences of cell death between TRCs and bulk tumor cells (Fig. 1h–j). Consistently, TRCs from A375 cells also showed enhanced tolerance to the three agents above (RSL3, FIN56 and FINO2) and IFNγ + AA, in which ferroptosis inhibitors could also exert similar effect (Supplementary Fig. 1f–i). To extend our findings, we used HCT116 cells, which were sensitive to cysteine deficiency, and found that HCT116-derived TRCs were more tolerant to both medium cystine starvation and erastin (Supplementary Fig. 1j). More importantly, under RSL3 treatment, TRCs exhibited decreased formation of di-oxygenated and tri-oxygenated PUFA-containing PE species, especially ferroptosis PL signals di-oxygenated and tri-oxygenated arachidonoyl and adrenoyl-PE species (Fig. 1k and Extended Data Fig. 1k,l). Together, these data suggest that TRCs mediate resistance to radiation or cisplatin through diminishing lipid peroxidation and ferroptosis.

### PL remodeling of TRCs inhibits ferroptosis

**Extended Data Fig. 2:**
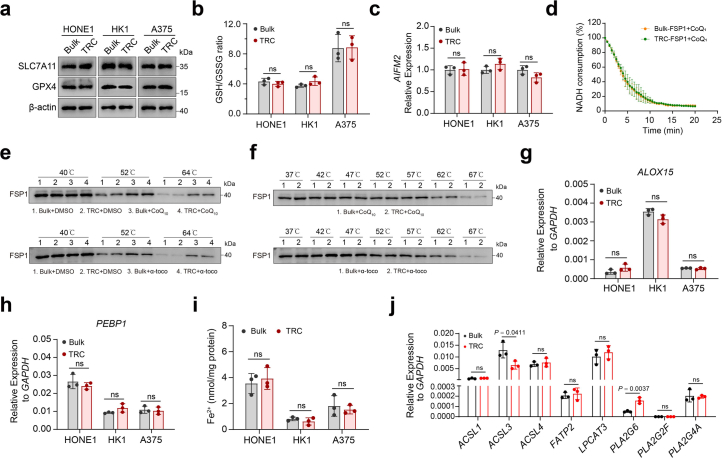
Interrogation the involvement of previously identified key genes in mediating resistance to ferroptosis in TRCs. **a**, Immunoblots showing the expression of GPX4 and SLC7A11 in TRCs and bulk cells. **b**, GSH/GSSG ratios in TRCs and bulk cells. **c**, The relative mRNA expression of *AIFM2* (encoding FSP1 protein) in TRCs and bulk tumor cells. **d**, NADH consumption assay with CoQ_1_ to determine the FSP1 activity. **e**, Representative results of a cellular thermal shift assay (CETSA). FSP1 protein was resistant to heat denaturation in the presence of CoQ_10_ (upper) and α-tocopherol (lower). **f**, Representative results of a cellular thermal shift assay (CETSA). FSP1 protein from HONE1 bulk tumor cells and TRCs showed similar heat denaturation in the presence of CoQ_10_ (upper) and α- tocopherol (lower). **g-h**, The relative mRNA expression of *ALOX15* **(i)** or *PEBP1***(j)** in TRCs and bulk tumor cells. *P* = 0.1556, 0.0683, 0.4532, 0.1007, 0.1243 and 0.7519. **i**, The cellular ferrous iron content of TRCs and bulk tumor cells was measured. *P* = 0.5932, 0.3396 and 0.6071. **j**, The relative mRNA expression of *ACSL1*, *ACSL3*, *ACSL4*, *FATP2*, *LPCAT3*, *PLA2G6*, *PLA2G2F* and *PLA2G4A* in TRCs and bulk tumor cells from HONE1 cells. Data are shown as mean ± SD, unpaired two-tailed *t*-test (b-c, g- j), n = 3 independent experiments. Bulk, black points; TRC, red points. One of three experiments is shown (a, e-f). ns, not significant. Source data

Iron-dependent PL peroxidation, lipid peroxidation detoxification system and membrane PL composition are the three pillars in determining cell ferroptosis sensitivity^24^. We previously showed that TRCs were insensitive to System X_c_^−^/GSH/GPX4 blocking (Fig. 1e,f,h,i,k, Extended Data Fig. 1h,i,l and Supplementary Fig. 1f,g), and the expression of key genes (SLC7A11 and GPX4) and intracellular GSH levels showed no significant difference between bulk tumor cells and TRCs (Extended Data Fig. 2a,b). We then investigated the involvement of the FSP1-CoQ_10_-NAD(P)H pathway, a standalone parallel lipid peroxidation detoxification system^25^. We found that not only were there no differences in FSP1 expression between TRCs and bulk cells but also the enzyme catalytic activity was similar (Extended Data Fig. 2c–f). Consistently, knocking down *AIFM2* (encoding the FSP1 protein) could mildly sensitize TRCs to RSL3, and the extent of this heightened sensitivity was not as pronounced as that observed in bulk tumor cells (Supplementary Fig. 2a,b). We further explored the role of iron-associated PL peroxidation. 15-LOX (refs. ^26,27^) and its scaffold protein PEBP1, as well as ferrous iron^24^, play decisive roles in this scenario. However, we observed no significant differences in the 15-LOX and PEBP1 expression levels or ferrous iron levels between TRCs and bulk tumor cells from various cell lines (Extended Data Fig. 2g–i). These data suggest that the iron-dependent PL peroxidation and lipid peroxidation detoxification system might not contribute to the ferroptosis tolerance of TRCs.

**Fig. 2:**
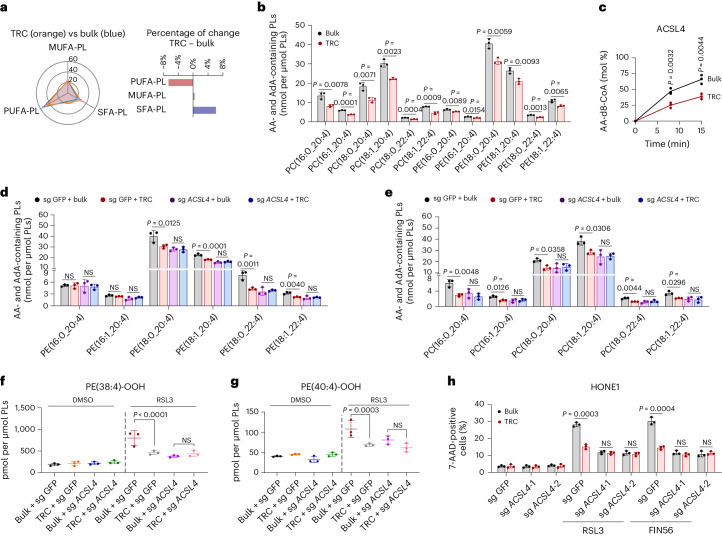
ACSL4-dependent PL remodeling is required for ferroptosis resistance of TRCs. **a**, The radar chart illustrates changes of SFA-PLs, MUFA-PLs and PUFA-PLs in HONE1 TRCs and bulk tumor cells (left). The bar chart (right) represents the relative increment of these PLs. **b**, The content of esterified AA (C20:4) or AdA (C22:4) PE or PC molecular species in HONE1 TRCs and bulk cells. **c**, The formation rate of AA-d8-CoA in reactions catalyzed by ACSL4 purified from HONE1 TRCs and bulk cells at different timepoints. AA-d8 and coenzyme A were used as substrates. AA-d8-CoA was undetectable in the samples with no enzyme added. **d**, The content of esterified AA (C20:4) or AdA (C22:4) PE molecular species in TRCs and bulk tumor cells from *ACSL4*-knockout HONE1 cells and parental cells. **e**, The content of esterified AA (C20:4) or AdA (C22:4) PC molecular species in TRCs and bulk tumor cells from *ACSL4*-knockout HONE1 cells and parental cells. **f**,**g**, TRCs and bulk cells from *ACSL4*-knockout HONE1 cells (sg *ACSL4*) or parental cells (sg GFP) were treated with RSL3 (15 µM) for 13 h. The content of ferroptotic cell death signal PE (38:4)-OOH (**f**) and PE (40:4)-OOH (**g**) was measured by LC–MS/MS. *P* = 0.0000596 and 0.5509789. **h**, TRCs and bulk tumor cells from *ACSL4*-knockout HONE1 cells or parental cells were treated with RSL3 (15 µM) or FIN56 (10 µM). The percentage of dead cells was measured after indicated treatment for 16 h. Data are shown as mean ± s.d.; unpaired two-tailed *t*-test (**b**,**c**,**h**) or one-way ANOVA (**d**–**g**). *n* = 3 independent experiments. NS, not significant. Source data

Next, we explored whether there were differences in PL composition between TRCs and bulk tumor cells. LC–MS lipidomic analysis showed that TRCs exhibited decreased PUFA-PLs, especially AA (C20:4)-containing and adrenic acid (AdA) (C22:4)-containing PE or phosphatidylcholine (PC) species, which were strongly associated with ferroptosis (Fig. 2a,b). Indeed, the balance between monounsaturated fatty acid (MUFA)-PLs and PUFA-PLs profoundly influences cell ferroptosis sensitivity^28^. Lands’ cycle is the core mechanism for fatty acid replacement at the *sn-2* position of PLs, leading to PL composition remodeling^29^. Acyl-CoA synthetases (ACSs), lyso-PL acyltransferases (LPLATs) and phospholipases A2 (PLA2s) constitute pivotal members to maintain this mechanism, which is involved in providing activated fatty acid and in catalyzing the acylation reaction and the de-acylation reaction, respectively. Considering that numerous molecules are involved in Lands’ cycle, we focused on several key genes known to be involved in ferroptosis for further analysis. For ACSs, ACSL1, ACSL3 (ref. ^30^), ACSL4 (refs. ^6,10^) and FATP2 (refs. ^31,32^) were included; for LPLATs, LPCAT3 (ref. ^33^) was included; and for PLA2s, PLA2G6 (ref. ^34^), PLA2G2F (ref. ^28^) and PLA2G4A (ref. ^35^) were included. Through quantitative reverse transcription PCR (RT–qPCR), we found that *PLA2G2F* was undetectable and the expression levels of *FATP2*, *PLA2G6* and *PLA2G4A* were low, whereas other genes were expressed at relatively high levels in HONE1 cells. Interestingly, most gene expressions exhibited no significant differences between TRCs and bulk tumor cells, except for *ACSL3* and *PLA2G6*, which were noticeably downregulated and upregulated in TRCs, respectively (Extended Data Fig. 2j). On the other hand, by siRNA screening, we observed that knocking down any of these genes did not change the sensitivity of TRCs to RSL3, whereas knocking down of *ACSL4* or *LPCAT3* significantly decreased the sensitivity of cells to RSL3 in bulk tumor cells (Supplementary Fig. 2c–j). Notably, bulk tumor cells with *ACSL4* knockdown exhibited similar RSL3-induced cell death to that of TRCs with or without *ACSL4* knockdown (Supplementary Fig. 2f). Consistent with previous studies^36^, knocking down of *ACSL3* mildly increased the sensitivity of bulk tumor cells to RSL3 (Supplementary Fig. 2e). However, considering that *ACSL3* was downregulated in TRCs (Extended Data Fig. 2j), its potential role in mediating resistance to ferroptosis in TRCs could be excluded.

**Extended Data Fig. 3:**
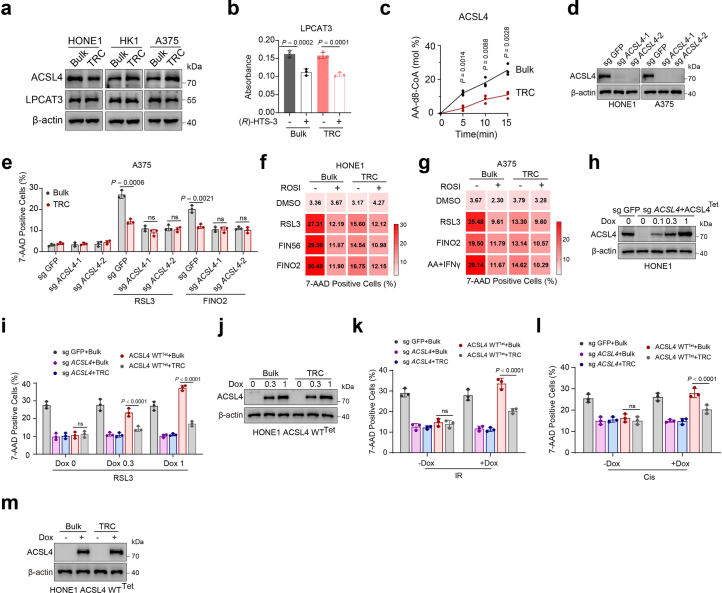
ACSL4 dependent phospholipid remodeling is required for ferroptosis resistance of TRCs. **a**, Immunoblots showing the expression of ACSL4 and LPCAT3 in TRCs and bulk tumor cells. **b**, Cell lysates of TRCs and bulk tumor cells from HONE1 cells were incubated with or without LPCAT3 inhibitors, (*R*)-HTS-3. LPCAT3 enzymatic activity was assessed. **c**, Cell lysates of TRCs and bulk tumor cells from HONE1 cells were incubated with or without ACSL4 inhibitors, Rosiglitazone. The formation of AA-d8-CoA in reactions was detected by LC-MS. **d**, Immunoblots showing the expression of ACSL4 in *ACSL4*-knockout HONE1 cells and A375 cells. **e**, TRCs and bulk cells from *ACSL4*-knockout A375 cells or parental cells were treated with RSL3 or FINO2. The percentage of dead cells was measured. **f-g**, Percentage of dead cells in TRCs and bulk tumor cells from HONE1 cells **(f)** or A375 cells **(g)** with indicated treatment. Rosiglitazone (ROSI, 10 µM) was added before the treatment of ferroptosis inducers. **h**, Immunoblots showing the expression of ACSL4 in *ACSL4*- knockout HONE1 cells transfected with Dox-inducible ACSL4-WT expression plasmids. Cells were treated with or without Dox. **i**, The parental HONE1 cells (sg GFP), TRCs and bulk tumor cells from *ACSL4*-knockout HONE1 cells (sg *ACSL4*) and *ACSL4*-knockout HONE1 cells stably expressing Dox-inducible ACSL4-wild type (ACSL4 WT^Tet^) were treated with Dox before RSL3 treatment. The percentage of dead cells was measured by 7-AAD staining and flow cytometry. *P* = 0.8154, 0.0000173 and 0.000000000998. **j**, Immunoblots showing the expression of ACSL4 in the cells depicted in **(i)**. **k-l**, The parental HONE1 cells (sg GFP), TRCs and bulk tumor cells from *ACSL4*-knockout HONE1 cells (sg *ACSL4*) and *ACSL4*- knockout HONE1 cells stably expressing Dox-inducible ACSL4-wild type (ACSL4 WT^Tet^) were treated with or without Dox, followed by treatment with IR **(k)** or cisplatin (Cis) **(l)**. The percentage of dead cells was measured. *P* = 0.0951, 0.0000000432, 0.6187 and 0.00000273. **m**, Immunoblots showing the expression of ACSL4 in the cells depicted in **(k-l)**. Data are shown as mean ± SD, unpaired two-tailed *t*-test (b-c, e) or one-way ANOVA (i, k-l), n = 3 independent experiments. One of three experiments is shown (a, d, h, j, m). ns, not significant. Source data

Based on the above data, we speculated that iPLA2β (encoded by the *PLA2G6* gene), LPCAT3 or ACSL4 may play a pivotal role in PL remodeling and decreased ferroptosis sensitivity in TRCs. Although knockdown of *PLA2G6* showed no obvious effect in both bulk tumor cells and TRCs (Supplementary Fig. 2i), *PLA2G6* was upregulated approximately three-fold in TRCs (Extended Data Fig. 2j). Therefore, we further explored whether the expression change magnitude of *PLA2G6* was sufficient for TRCs to avert ferroptosis. To this regard, by evaluating the conversion rate of its substrates 1-SA-2-15-HpETE-PE or 1-SA-2-ETE-PE to 1-SA-2-OH-PE, we observed no differences in the catalytic activity of iPLA2β purified from HONE1 TRCs and bulk tumor cells (Supplementary Fig. 2k,l). We next generated inducible PLA2G6 overexpression HONE1 cells and found that a 50–100-fold upregulation from baseline was needed for iPLA2β to manifest significant inhibitory effects on ferroptosis (Supplementary Fig. 2m,n). Based on these data, it is unlikely that iPLA2β contributed to the ferroptosis resistance observed in TRCs. Given that the protein levels of ACSL4 and LPCAT3 were similar between TRCs and bulk tumor cells (Extended Data Fig. 3a), we tested whether there were differences in the enzymatic activity. By using specific inhibitors toward LPCAT3 ((*R*)-HTS-3) or ACSL4 (rosiglitazone) in enzymatic activity assays, we found that the substrate conversion rate of LPCAT3 did not exhibit differences between TRCs and bulk tumor cells, whereas ACSL4 catalytic activity was significantly decreased in TRCs (Extended Data Fig. 3b,c and Supplementary Fig. 3a–c). We further purified ACSL4 from HONE1 bulk tumor cells and TRCs, and enzyme activity assay indicated that ACSL4 from TRCs resulted in a lower product (AA-d8-CoA) formation than those from bulk tumor cells (Fig. 2c).

Thus, we speculated that ACSL4 activity was dampened in TRCs, leading to decreased PL unsaturation and ferroptosis resistance. As expected, the bulk tumor cells and TRCs from *ACSL4*-knockout cells showed similar PL unsaturation patterns and ferroptosis sensitivity, both of which were at similar levels with TRCs from wild-type (WT) cells (Fig. 2d–h and Extended Data Fig. 3d,e). Consistently, inhibition of ACSL4 via rosiglitazone almost abolished ferroptosis differences between bulk tumor cells and TRCs (Extended Data Fig. 3f,g). In addition, we ruled out the possibility that rosiglitazone exerted ferroptosis protective effect through targets (PPARγ^37^ and AMPK^38,39^) other than ACSL4 (Supplementary Fig. 4a–c). Furthermore, we re-expressed ACSL4 with inducible pattern in ACSL4^−/−^ HONE1 cells (Extended Data Fig. 3h). We observed that overexpression of ACSL4 sensitized bulk tumor cells to RSL3 but did not confer an apparent effect in TRCs, despite the similar levels of ACSL4 expression between these two groups (Extended Data Fig. 3i,j). Re-expression of ACSL4 also sensitized the response to cisplatin or radiation only in bulk tumor cells but not in TRCs (Extended Data Fig. 3k–m). Taken together, these findings suggest that TRCs resist ferroptosis via ACSL4-dependent cellular PL remodeling.

### T679 phosphorylation is the key event for ACSL4 to function

**Fig. 3:**
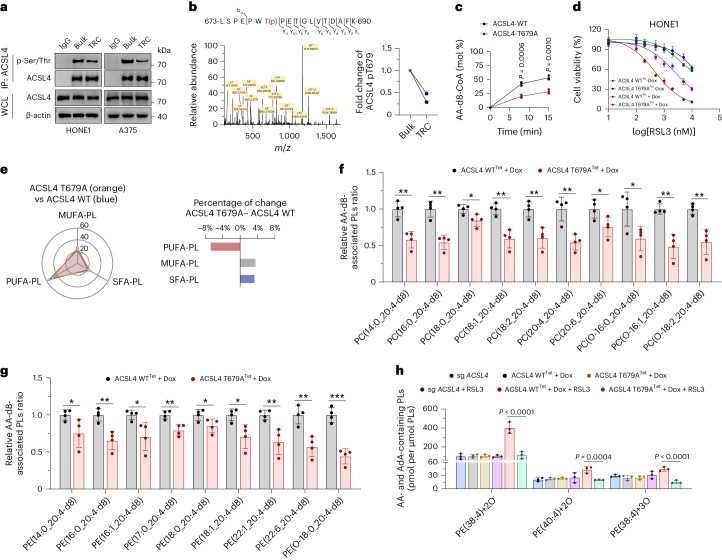
T679 phosphorylation is the key event for ACSL4 to function. **a**, Immunoprecipitation and immunoblot analysis of the level of phospho-Ser/Thr ACSL4 in TRCs and bulk tumor cells (HONE1 and A375). **b**, Mass spectrometry analysis of the phosphorylation site in ACSL4 protein purified from HONE1 TRCs or bulk cells (left). The percentage of ACSL4 Thr679 phosphorylation in HONE1 TRCs and bulk tumor cells (right). **c**, The formation rate of AA-d8-CoA in reactions catalyzed by ACSL4 protein purified from Dox-inducible ACSL4-WT HONE1 cells or Dox-inducible ACSL4-T679A HONE1 cells. AA-d8 and coenzyme A were used as substrates. AA-d8-CoA was undetectable in the samples with no enzyme added. **d**, Dose-dependent toxicity of RSL3-induced cell death of *ACSL4*-knockout HONE1 cells stably expressing Dox-inducible ACSL4-WT (ACSL4 WT^Tet^) or Dox-inducible ACSL4-T679A (ACSL4 T679A^Tet^) with or without Dox (0.3 μg ml^−1^). **e**, The radar chart indicates the changes of SFA-PLs, MUFA-PLs and PUFA-PLs in Dox-inducible ACSL4-WT HONE1 cells or Dox-inducible ACSL4-T679A HONE1 cells (left). The relative increment of these PLs is represented by a bar chart (right). **f**,**g**, Dox-inducible ACSL4-WT HONE1 cells or Dox-inducible ACSL4-T679A HONE1 cells were treated with Dox (0.3 μg ml^−1^) followed by AA-d8 (10 µM) for 36 h. The relative changes of PC (**f**) or PE (**g**) that contain AA (20:4)-d8 are shown. *n* = 4. *P < 0.05; **P < 0.01; ***P < 0.001. **f**, *P* = 0.0017, 0.0008, 0.0236, 0.0017, 0.0045, 0.0035, 0.0373, 0.0312, 0.0013 and 0.0030. **g**, *P* = 0.0481, 0.0039, 0.0263, 0.0045, 0.0446, 0.0164, 0.0073, 0.0035 and 0.0002. **h**, Accumulation of RSL3-induced oxygenated PE that contains AA or AdA in *ACSL4*-knockout HONE1 cells (sg *ACSL4*), Dox-inducible ACSL4-WT expression HONE1 cells (ACSL4 WT^Tet^) or Dox-inducible ACSL4-T679A expression HONE1 cells (ACSL4 T679A^Tet^) treated with Dox (0.3 μg ml^−1^). *P* = 0.000000182, 0.0004 and 0.0000171. Data are shown as mean ± s.d.; unpaired two-tailed *t*-test (**c**,**f**,**g**) or one-way ANOVA (**h**). *n* = 3 independent experiments. One of three experiments is shown (**a**). Source data

**Extended Data Fig. 4:**
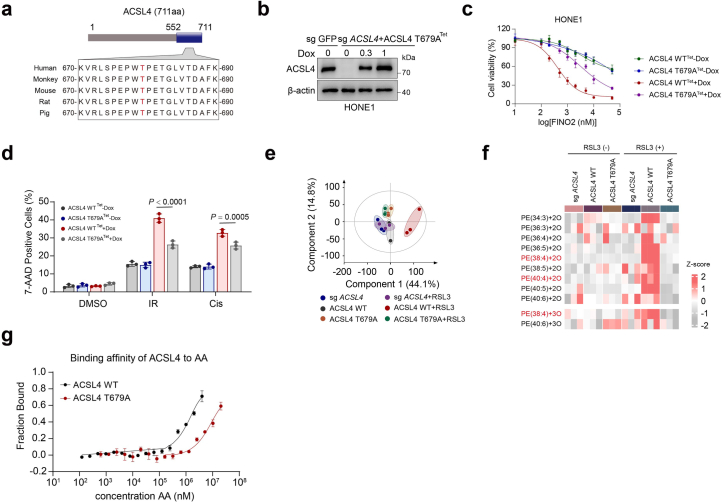
T679 phosphorylation is the key event for ACSL4 to function. **a**, Alignment of protein sequences spanning ACSL4 T679 from different species. **b**, Immunoblots showing the ACSL4 expression in *ACSL4*-knockout HONE1 cells transfected with Dox-inducible ACSL4-T679A mutation expression plasmids. Cells were treated without or with Dox. **c**, Dose-dependent toxicity of FINO2 induced cell death of *ACSL4*- knockout HONE1 cells stably expressing Doxycycline (Dox)- inducible ACSL4-wild type (ACSL4 WT^Tet^) or Dox-inducible ACSL4-T679A (ACSL4 T679A^Tet^) without or with Dox. **d**, Dox-inducible ACSL4-WT HONE1 cells or Dox-inducible ACSL4-T679A HONE1 cells pretreated with Dox, followed by IR or cisplatin. The percentage of dead cells was measured. *P* = 0.0000104 and 0.0005. **e**, Principal component analysis (PCA) of RSL3-induced oxygenated phospholipids in *ACSL4*-knockout HONE1 cells (sg ACSL4), Dox-inducible ACSL4-WT expression HONE1 cells (ACSL4 WT^Tet^) or Dox-inducible ACSL4-T679A expression HONE1 cells (ACSL4 T679A^Tet^) treated with Dox, with or without RSL3. Lipids were analyzed by LC-MS/MS. Each point represents a sample. **f**, Heatmap showing LC-MS analysis of the relative changes in RSL3-induced oxygenated PE molecular species in *ACSL4*-knockout HONE1 cells (sg ACSL4), Dox-inducible ACSL4-WT expression HONE1 cells (ACSL4 WT^Tet^) or Dox-inducible ACSL4-T679A expression HONE1 cells (ACSL4 T679A^Tet^) treated with Dox (0.3 μg/mL). Row z-scores were obtained from averaged values of the content of lipid species. **g**, Microscale thermophoresis (MST) demonstrated a direct interaction between Arachidonic acid and GFP-tagged ACSL4 in lysates from GFP-ACSL4-WT or GFP-ACSL4-T679A expressing HEK293T cells; mutation of T679A partly blocks the interaction. Data are shown as mean ± SD, one-way ANOVA (d), n = 3 independent experiments. One of three experiments is shown (b, g). aa, amino acid. Source data

The enzymatic function of ACSL4 is tightly regulated via post-transcriptional modifications, particularly phosphorylation^12^. We observed that the total phosphorylation level of ACSL4 was lower in TRCs than in bulk tumor cells (Fig. 3a). Because phosphorylation of different sites on ACSL4 may confer distinct biological effects, with mass spectrometric analysis, we found that the T679 residue phosphorylation level of ACSL4 was markedly decreased in HONE1-derived TRCs compared to bulk tumor cells (Fig. 3b). Moreover, this phosphorylation site was highly conserved among different species (Extended Data Fig. 4a).

To test whether T679 phosphorylation was critical for maintaining the enzymatic activity of ACSL4, we re-expressed inducible T679 non-phosphorylatable ACSL4 mutation (T679A) in ACSL4^−/−^ HONE1 cells (Extended Data Fig. 4b). Compared to ACSL4-WT, tumor cells re-expressing ACSL4-T679A showed dampened enzymatic activity and decreased ferroptosis (Fig. 3c,d and Extended Data Fig. 4c). Moreover, radiation and cisplatin induced much less cell death in tumor cells re-expressing ACSL4-T679A (Extended Data Fig. 4d). Consistently, compared to ACSL4-WT, re-expression of ACSL4-T679A led to significant decreased cellular PL unsaturation; specifically, PUFA-PL decreased by 6.8%, whereas MUFA-PL and saturated fatty acid (SFA)-PL increased by 3.5% and 3.3%, respectively (Fig. 3e). Furthermore, under AA-d8 treatment, cells re-expressing ACSL4-T679A contained less AA-d8-associated PE or PC species, particularly in C16 and C18 species (Fig. 3f,g and Supplementary Fig. 5a–d). ACSL4-T679A-re-expressing cells also had less di-oxygenated and tri-oxygenated PUFA-PE species under RSL3 treatment (Fig. 3h and Extended Data Fig. 4e,f).

Next, we conducted molecular dynamics simulation to explore the effect of T679 phosphorylation in enhancing ACSL4 enzymatic activity. Both ACSL4^WT^ model and ACSL4^pT679^ model can quickly reach a stable state within 25 ns, and they maintained this state throughout the simulation, whereas the ACSL4^pT679^ protein residues showed less root mean square fluctuation (RMSF) in three regions (B1, H1 and CTD) (Supplementary Fig. 6a–c). Principal component analysis (PCA) of ACSL4^WT^ protein model showed that the B1/H1/CTD motif gradually moved away from each other in PC1, and the B1 motif gradually moved away from the active pocket in PC2, whereas, in the ACSL4^pT679^ protein model, the B1/H1 /CTD motif approached each other and the B1 motif tended to approach the active pocket (Supplementary Fig. 6d–i and Supplementary Videos 1–4). These results indicate that the phosphorylation of T679 enables ACSL4 to form a more stable and compact active pocket, which provides a better basis for enzyme activity.

We then analyzed the differences in the binding of three substrates (ATP, AA and CoA) to ACSL4 in different states. The binding of ATP exhibited no differences in ACSL4^WT^ protein and ACSL4^pT679^ protein (Supplementary Fig. 6j), whereas the binding of AA to ACSL4^pT679^ protein was more stable than ACSL4^WT^ protein (Supplementary Fig. 6k). Consistently, microscale thermophoresis (MST) experiment further showed that T679A mutation markedly decreased the interaction between ACSL4 and AA (Extended Data Fig. 4g). For CoA, we found that the S atom on CoA tended to be away from AA in ACSL4^WT^ protein model, whereas ACSL4-pT679 hindered the tendency of CoA to move away from AA and T679 in ACSL4^pT679^ protein model (Supplementary Fig. 6l–q and Supplementary Videos 5–8). These results indicate that T679 phosphorylation affects ACSL4 catalytic activity mainly by altering the state of the AA and CoA binding to ACSL4.

Together, these data suggest that T679 phosphorylation is critical for maintaining ACSL4 enzymatic activity and determines cell sensitivity to ferroptosis.

### PCK2 phosphorylates ACSL4 at T679 to enhance ferroptosis

**Fig. 4:**
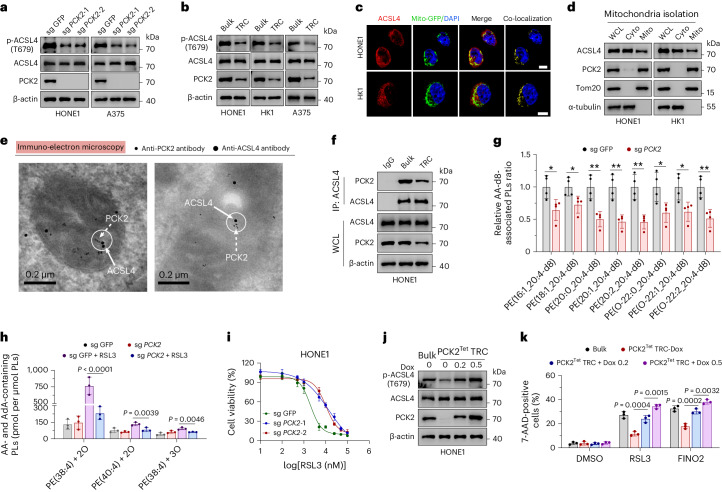
PCK2 directly phosphorylates ACSL4 at T679 to enhance ferroptosis sensitivity. **a**, Immunoblots showing the expression of ACSL4 and p-ACSL4(T679) in *PCK2*-knockout cells and parental cells. **b**, Immunoblots showing the expression of ACSL4 and p-ACSL4(T679) in TRCs and bulk tumor cells. **c**, Representative immunofluorescence staining image showing the co-localization of ACSL4 and mitochondria in cells stably transfected with mito-GFP. Scale bar, 10 μm. **d**, Immunoblots showing the localization of ACSL4 in mitochondria. Cyto, cytosol; Mito, mitochondria; WCL, whole-cell lysate. Tom20 and α-tubulin are markers for mitochondria and cytosol, respectively. **e**, Immunoelectron microscopic localization of ACSL4 and PCK2 in the mitochondria by using a gold-labeled anti-ACSL4 antibody (15 nm gold) and a gold-labeled anti-PCK2 antibody (6 nm gold). The co-localization of ACSL4 and PCK2 in sections of HONE1 cells is shown. Bars, 0.2 μm. **f**, Immunoprecipitation and immunoblotting showing the interaction of ACSL4 and PCK2 in HONE1 TRCs and bulk cells. **g**, *PCK2*-knockout HONE1 cells and parental cells were treated with AA-d8 (10 µM). The relative changes of PE that contain AA (20:4)-d8 are shown. *n* = 4. *P < 0.05; **P < 0.01; ***P < 0.001. *P* = 0.0254, 0.0294, 0.0038, 0.0024, 0.0027, 0.0267, 0.0225 and 0.004. **h**, Accumulation of RSL3-induced oxPE that contains AA or AdA in *PCK2*-knockout HONE1 cells and parental cells. *P* = 0.0000417, 0.0039 and 0.0046. **i**, Dose-dependent toxicity of RSL3-induced cell death of *PCK2*-knockout HONE1 cells (sg *PCK2*) and parental cells (sg GFP). **j**, Parental HONE1 cells and TRCs from *PCK2*-knockout HONE1 cells stably expressing Dox-inducible PCK2 (PCK2^Tet^) were treated without or with Dox. Immunoblots showing the expression of p-ACSL4(T679) and ACSL4 in cells. **k**, The cells depicted in **j** were treated with or without Dox before RSL3 or FINO2 treatment (15 μM). The percentage of dead cells was measured by 7-AAD staining and flow cytometry. Data are shown as mean ± s.d. (**g**,**h**,**k**); unpaired two-tailed *t*-test (**g**) or one-way ANOVA (**h**,**k**). *n* = 3 independent experiments. One of three experiments is shown (**a**,**b**,**d**,**f**,**j**). Source data

**Extended Data Fig. 5:**
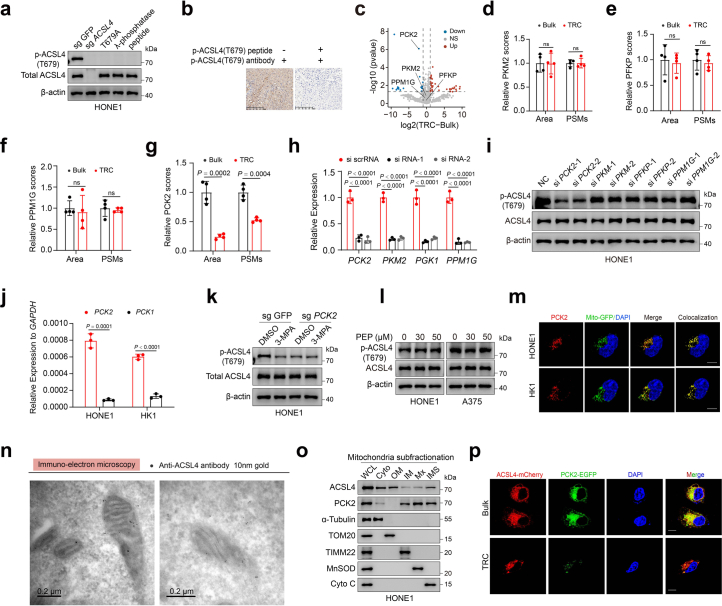
PCK2 phosphorylates ACSL4 at T679. **a**, Immunoblots were performed with parental HONE1 cells (sg GFP), ACSL4-knockout HONE1 cells (sg ACSL4), *ACSL4*-knockout HONE1 cells stably expressing ACSL4 T679A mutation (T679A), treating cell lysates with λ protein phosphatase (λ-phosphatase) or in the presence of blocking peptide for ACSL4 Thr679 phosphorylation (peptide). The p-ACSL4 (T679) antibody was used to detect the phosphorylation level of ACSL4 T679 site. **b**, IHC analyses of human NPC samples were performed with the p-ACSL4(T679) antibody in the presence or absence of a blocking peptide for p-ACSL4(T679). **c-g**, LC–MS/MS detection for ACSL4-interacting proteins in ACSL4-containing protein complex immunoprecipitated from HONE1 TRCs and bulk cells. Volcano plot showing the fold change and −log_10_ (*P*-value of two-sided Student’s *t*-test) of differential interaction proteins in HONE1 TRCs and bulk cells. Dotted line indicates factors with fold change > 2 and significance *P* < 0.05 **(c)**. The peak area and PSMs of PKM2 **(d)**, PFKP **(e)**, PPM1G **(f)** and PCK2 **(g)** are shown. n = 4. **h**, The relative mRNA expressions of indicated genes were analyzed by qRT-PCR for gene knocked-down efficiency in HONE1 cells. *P* = 0.00005, 0.000040, 0.0000050, 0.000005, 0.00003, 0.000048, 0.000013 and 0.000012. **i**, Immunoblots showing the expression of ACSL4 and ACSL4 pT679 in HONE1 cells transfected with indicated siRNAs. **j**, The relative mRNA expression of *PCK2* and *PCK1* in HONE1 and HK1 cells. *P* = 0.0001 and 0.0000366. **k**, *PCK2*-knockout HONE1 cells and parental cells were treated with or without 3-MPA. Immunoblots showing the expression of p-ACSL4(T679) and ACSL4 in cells. **l**, Immunoblots showing the expression of ACSL4 and ACSL4 pT679 in HONE1 cells or A375 cells treated with phosphoenolpyruvate (PEP). **m**, Representative immunofluorescence staining image showing the co-localization of PCK2 (red) and mitochondria (green) in HONE1 cells and HK1 cells stably transfected with mito-GFP. Scale bar: 10 μm. **n**, Immunoelectron microscopy of ACSL4 localization in mitochondria in HONE1 cells by using a gold-labeled anti-ACSL4 antibody (10 nm gold). Bars: 0.2 μm. **o**, Subfractionation of highly purified mitochondria from HONE1 cells showing the presence of ACSL4 protein in mitochondria of HONE1 cells and HK1 cells. WCL, whole cell lysate; Cyto, cytosol; OM, mitochondria outer membrane; IM, inner membrane; Mx, mitochondria matrix; IMS, mitochondria intermembrane space. α-Tubulin, Tom20, TIMM22, MnSOD and Cyto C are markers for cytosol, mitochondria outer membrane (OM), inner membrane (IM), matrix (Mx) and inter-membrane space (IMS), respectively. **p**, Cells expressing ACSL4-mCherry and PCK2-GFP were seeded in 3D fibrin gels (TRCs) or not (bulk cells). Immunofluorescence analysis to detect ACSL4 (red) or PCK2 (green). Scale bar: 10 μm. Data are shown as mean ± SD (d-h, j), unpaired two-tailed *t*-test (d-g, j) or one-way ANOVA (h), n = 3 independent experiments. One of three experiments is shown (a, i, k-l, o). Source data

Next, we sought to identify the key kinases or phosphatases that regulate T679 phosphorylation of ACSL4. To facilitate subsequent experiments, we manufactured a phospho-Thr679-ACSL4-specific (p-ACSL4(T679)) antibody and tested its specificity by immunoblotting and immunohistochemistry (IHC) staining analysis (Extended Data Fig. 5a,b). By quantitative mass spectrometry analysis with anti-ACSL4 immunoprecipitates from lysates of HONE1 bulk tumor cells or TRCs, we identified kinases PCK2, PKM2 and PFKP, as well as phosphatase PPM1G, from the top 250 most enriched proteins in the precipitates. Interestingly, PCK2, PKM2 and PFKP were all non-canonical kinases involved in glycolysis or gluconeogenesis metabolism, whereas no canonical kinase was identified among the top 250 proteins. The abundance of PCK2 was markedly decreased in precipitates from TRCs, whereas PKM2, PFKP and PPM1G showed no significant changes (Extended Data Fig. 5c–g). To test whether PCK2 was critical in mediating the phosphorylation of T679 on ACSL4, we conducted siRNA transfection targeting above genes. As expected, only knocking down of *PCK2* led to markedly decreased ACSL4(T679) phosphorylation level (Extended Data Fig. 5h,i). More convincingly, we also confirmed the role of PCK2 in regulating ACSL4(T679) in *PCK2*-knockout (*PCK2*^−/−^) cells (Fig. 4a). 3-Mercaptopicolinic acid (3-MPA) is a specific pharmacological inhibitor of both PCK1 and PCK2 (ref. ^40^), whereas PCK1 was barely detected in HONE1 and HK1 cells (Extended Data Fig. 5j). Consistently, 3-MPA inhibited the level of p-ACSL4(T679) in HONE1 WT cells but showed no obvious effect on *PCK2*-knockout HONE1 cells (Extended Data Fig. 5k). Moreover, the levels of PCK2 and p-ACSL4(T679) were downregulated in TRCs compared to bulk tumor cells in multiple cell lines as well (Fig. 4b).

**Extended Data Fig. 6:**
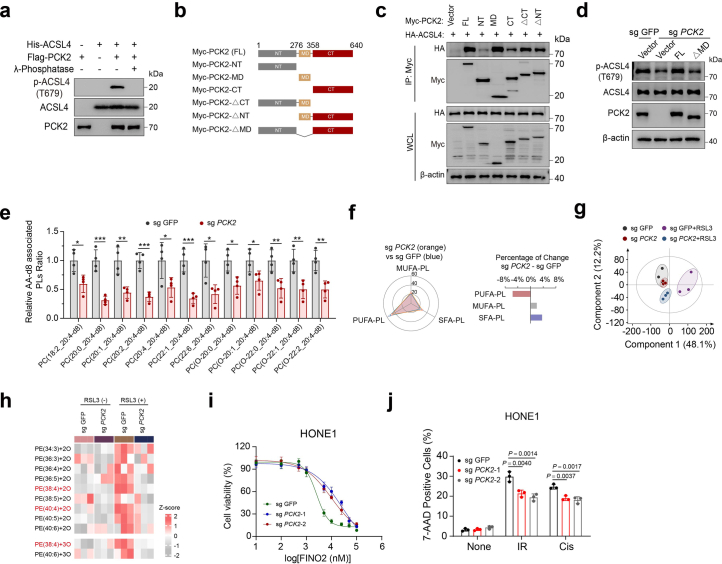
PCK2 directly phosphorylates ACSL4 and promotes its activity. **a**, *In vitro* kinase assays were performed by mixing *Escherichia coli*-purified His-ACSL4 (AA552-711) and 293T-purified active Flag-PCK2 with or without λ-Phosphatase. **b**, Schematic of PCK2 full-length and truncated mutants. **c**, HEK293T cells were transfected with the indicated Myc-tagged constructs, the ACSL4-PCK2 interaction was analyzed by immunoprecipitation with anti-Myc beads and immunoblotting. **d**, Immunoblots showing the expression of PCK2, ACSL4 and ACSL4 pT679 in HONE1 cells that expressing PCK2 wild type (FL) or catalytic domain-deletion mutation (ΔMD). **e**, *PCK2*-knockout HONE1 cells and parental cells were treated with AA-d8 (10 µM) for 36 h. The relative changes of PC that contain AA (20:4)-d8 are shown. n = 4. * *P* < 0.05; ** *P* < 0.01; *** *P* < 0.001. *P* = 0.0157, 0.0004, 0.0047, 0.0002, 0.0395, 0.0009, 0.0124, 0.0103, 0.036, 0.0085, 0.0076 and 0.0048. **f**, Radar chart indicates the changes of SFA-PLs, MUFA-PLs, and PUFA-PLs in *PCK2*-knockout cells and parental cells (upper). The relative increment of these phospholipids was represented by a bar chart (lower). **g**, Principal component analysis (PCA) of oxygenated phospholipids in *PCK2*-knockout cells and parental cells in the present or in the absence of RSL3. **h**, LC-MS-based heatmap showing relative changes in RSL3-induced oxygenated PE molecular species in *PCK2*-knockout HONE1 cells and parental cells. Row z-scores were obtained from averaged values of the content of lipid species. **i**, Dose-dependent toxicity of FINO2 induced cell death of *PCK2*-knockout HONE1 cells and parental cells. **j**, Percentage of dead cells in *PCK2*-knockout HONE1 cells and parental cells treated with IR or Cis. Data are shown as mean ± SD (e, j), unpaired two-tailed *t*-test (e) or one-way ANOVA (j), n = 3 independent experiments. One of three experiments is shown (a, c-d). Source data

Given the mitochondria localization of PCK2, we sought to explore whether PCK2 regulated ACSL4 through direct interaction. By treating cells with phosphoenolpyruvate, a direct metabolite of PCK2, we excluded the possibility that PCK2 regulated pACSL4(T679) in a metabolism-dependent manner (Extended Data Fig. 5l). Next, we explored the possibility that PCK2 and ACSL4 interacted spatially. Multiple studies demonstrated that ACSL4 could localize in mitochondria^41,42^. Through immunofluorescence and cellular fraction analysis, we found that PCK2 was indeed strictly distributed in mitochondria, whereas ACSL4 was distributed both inside and outside of the mitochondria (Fig. 4c,d and Extended Data Fig. 5m). Consistently, the presence of ACSL4 within mitochondria was also confirmed through immune electron microscopy (Extended Data Fig. 5n). More convincingly, mitochondria subfractionation analysis demonstrated that PCK2 resided in mitochondria matrix (Mx) and intermembrane space (IMS), whereas ACSL4 was mainly located in outer membrane of mitochondria (OM) and IMS (Extended Data Fig. 5o). Through immunofluorescence and immune electron microscopy, we found that PCK2 and ACSL4 could co-localize in mitochondria. However, immunofluorescence also demonstrated that such co-localization decreased in TRCs compared to bulk tumor cells (Fig. 4e and Extended Data Fig. 5p). Consistently, co-immunoprecipitation and immunoblotting analysis showed that PCK2 and ACSL4 interacted with each other endogenously, and such interaction was dampened in TRCs as PCK2 was downregulated (Fig. 4f). Moreover, in vitro kinase assays showed that PCK2 could directly phosphorylate ACSL4, and the phosphorylation was abrogated on addition of λ-phosphatase (Extended Data Fig. 6a).

We further analyzed the interaction between PCK2 and ACSL4 in detail by roughly dividing PCK2 into amino-terminal, catalytic and carboxy-terminal domains and constructed a series of MYC-tagged PCK2 truncated mutants (Extended Data Fig. 6b). Co-immunoprecipitation and immunoblotting analysis demonstrated that PCK2 bound to ACSL4 mainly through its catalytic domain (Extended Data Fig. 6c). More importantly, re-expressing PCK2-ΔMD (residues 1–276 and 359–640, lack of catalytic domain) in *PCK2*^−/−^ HONE1 failed to rescue the level of pACSL4(T679), in contrast to re-expression of full-length PCK2 (Extended Data Fig. 6d).

Next, we verified the role of PCK2 in ferroptosis. Upon treatment with AA-d8, *PCK2*^−/−^ cells contained less AA-d8-containing PE or PC species compared to WT cells (Fig. 4g and Extended Data Fig. 6e). Consistently, *PCK2*^−/−^ cells showed decreased PL unsaturation, as indicated by a 5.6% decrease in PUFA-PL and a 2.0% and 3.6% increase in MUFA-PL and SFA-PL, respectively (Extended Data Fig. 6f). Furthermore, on ferroptosis inducer treatment, *PCK2*^−/−^ cells showed reduced di-oxygenated and tri-oxygenated PUFA-PE species as well as decreased ferroptosis compared to their counterparts (Fig. 4h,i and Extended Data Fig. 6g–i). Upon treatment with cisplatin or radiation, *PCK2*^−/−^ cells also showed decreased cell death (Extended Data Fig. 6j). Notably, with the exogenous expression of PCK2 increased in TRCs, there was a concurrent enhancement in the level of pACSL4(T679) and the cellular sensitivity to RSL3 (Fig. 4j,k).

**Extended Data Fig. 7:**
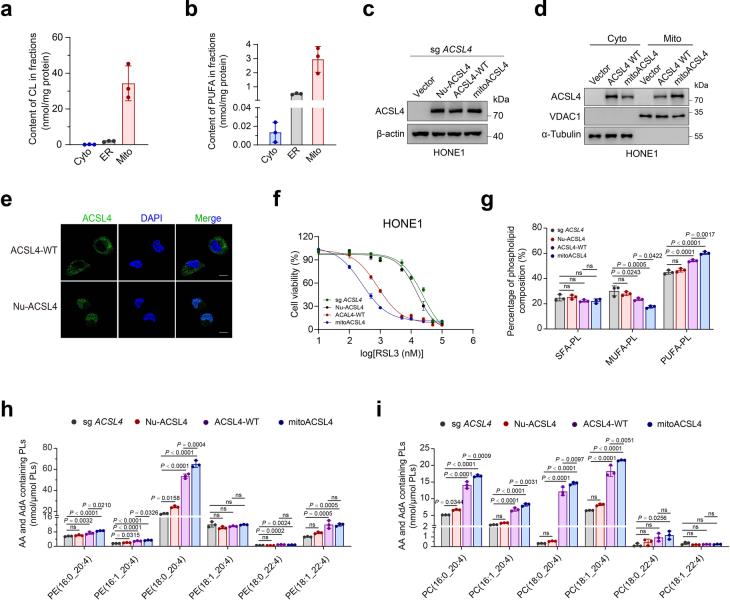
PCK2 increases cellular ferroptosis sensitivity via mitochondria located ACSL4. **a**, Comparison between the levels of cardiolipin (CL) in the cytoplasm (Cyto), endoplasmic reticulum (ER) and mitochondria (Mito). **b**, The content of PUFA in the cytoplasm (Cyto), endoplasmic reticulum (ER) and mitochondria (Mito). **c**, Immunoblot analysis of ACSL4 expression in ACSL4-knockout (sg ACSL4) HONE1 cells stably expressing vector (Vector), human NLS-ACSL4 (Nu-ACSL4), human ACSL4 wild type (ACSL4-WT), or human mito-ACSL4 (mito-ACSL4). **d**, Immunoblots showing increased ACSL4 mitochondrial localization in *ACSL4*-knockout HONE1 cells stably expressing mitoACSL4 (mitoACSL4) compared to ACSL4 wild type overexpressing HONE1 cells (ACSL4 WT). Cyto, cytosol; Mito, mitochondrial. VDAC1 and α-Tubulin are markers for mitochondrial and cytosol, respectively. **e**, Representative immunofluorescence staining image showing the co-localization of ACSL4 (green) and Nucleus (blue) in the ACSL4-knockout HONE1 cells stably expressing human ACSL4 wild type (ACSL4-WT) or human NLS-ACSL4 (Nu-ACSL4). Scale bar: 10 μm. **f**, Dose-dependent toxicity of RSL3 of the cells depicted in ACSL4- knockout (sg *ACSL4*) HONE1 cells stably expressing vector (Vector), human NLS-ACSL4 (Nu-ACSL4), human ACSL4 wild type (ACSL4-WT) or human mito-ACSL4 (mito-ACSL4). **g**, The relative phospholipid composition changes of cells in ACSL4-knockout (sg *ACSL4*) HONE1 cells stably expressing vector (Vector), human NLS-ACSL4 (Nu-ACSL4), human ACSL4 wild type (ACSL4-WT) or human mito-ACSL4 (mito-ACSL4). *P* = 0.9910, 0.4435, 0.4162, 0.999997, 0.6996, 0.0243, 0.0005, 0.0422, 0.5183, 0.0000991, 0.00000209 and 0.00167. **h**, The content of esterified AA (C20:4) or AdA (C22:4) PE molecular species in cells in (i) are shown. *P* = 0.5369, 0.0032, 0.000073, 0.021, 0.0315, 0.0000190, 0.00000224, 0.0326, 0.0158, 0.000000079, 0.00000000851, 0.0004, 0.0991, 0.4924, 0.9986, 0.6389, 0.7626, 0.0002, 0.0024, 0.1500, 0.1951, 0.0005, 0.0005, 0.99994. **i**, The content of esterified AA (C20:4) or AdA (C22:4) PC molecular species in cells in (i) are shown. *P* = 0.0344, 0.000000134, 0.0000000163, 0.000947, 0.2556, 0.000000694, 0.0000000808, 0.0031, 0.9897, 0.0000000844, 0.00000002, 0.0097, 0.1196, 0.00000042, 0.000000063, 0.0051, 0.7878, 0.0856, 0.0256, 0.8973, 0.0966, 0.307, 0.311, 0.999999996. Data are shown as mean ± SD (**a**-**b**, **g**-**i**), one-way ANOVA (**g**-**i**), n = 3 independent experiments. One of three experiments is shown (**c**, **d**). Source data

Then, we sought to further clarify the functional significance of mitochondrial distribution of ACSL4. First, by LC–MS analysis, the presence of PUFA in mitochondria was confirmed (Extended Data Fig. 7a,b). Second, we constructed an ACSL4 mutant fused with mitochondrial targeting sequence (mitoACSL4) and compared its effects with ACSL4-WT and another ACSL4 mutant fused with nuclear localization sequence (NLS-ACSL4) (Extended Data Fig. 7c–e). Compared to cells overexpressing ACSL4-WT, overexpression of mitoACSL4 exhibited increased sensitivity to ferroptosis and higher levels of PUFA-PLs, particularly arachidonoyl and adrenoyl-PE and PC species, whereas cells re-expressing NLS-ACSL4 showed similar levels of PUFA-PLs and ferroptosis sensitivity with that of ACSL4^−/−^ cells (Extended Data Fig. 7f–i). Collectively, these data suggest that the mitochondrial distribution of ACSL4 and PCK2-mediated ACSL4 T679 phosphorylation are key events to determine cellular PL remodeling and sensitivity to ferroptosis.

### STAT3 activation downregulates PCK2 in TRCs

**Fig. 5:**
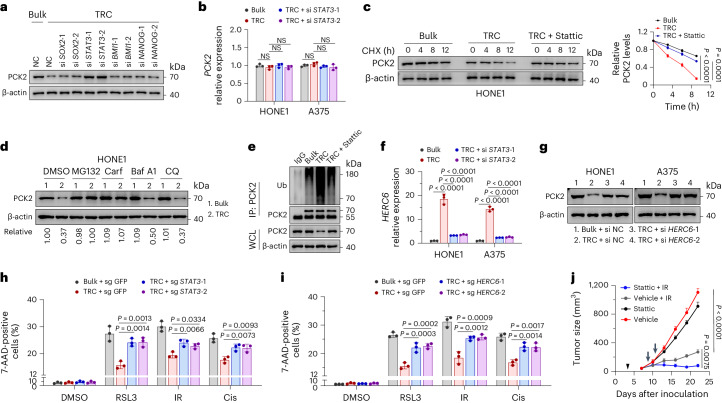
Activation of STAT3 in TRCs promotes PCK2 degradation. **a**, Immunoblots showing the expression of PCK2 in HONE1 TRCs and bulk tumor cells transfected with indicated siRNAs. **b**, RT–qPCR analysis of *PCK2* expression in HONE1 or A375 TRCs and bulk cells with *STAT3* knockdown. **c**, Inhibition STAT3 with Stattic (5 µM) stabilized PCK2 protein in HONE1 TRCs with CHX treatment for indicated times. The immunoblot analysis of PCK2 protein level (left) and corresponding grayscale analysis (right) are shown. *P* = 0.00000042 and 0.000002. **d**, Immunoblot analysis showing PCK2 protein level in HONE1 TRCs and bulk cells treated with MG132 (10 μM), carfilzomib (100 nM), bafilomycin A1 (Baf A1, 0.2 μM) or chloroquine (CQ, 50 μM) for 6 h. **e**, HONE1 TRCs and bulk cells treated with or without Stattic (5 µM), after which the cell lysates were subjected to immunoprecipitation with anti-PCK2 antibody and immunoblotting with anti-ubiquitin (Ub) antibody. **f**, RT–qPCR analysis of *HERC6* expression in HONE1 or A375 TRCs and bulk cells with *STAT3* knockdown. *P* = 0.0000001, 0.0000003, 0.00000037, 0.000000003, 0.0000000067 and 0.0000000077. **g**, Immunoblots showing the expression of PCK2 in TRCs and bulk tumor cells with or without *HERC6* knockdown. **h**,**i**, TRCs from *STAT3*-knockout HONE1 cells or parental cells and bulk HONE1 cells were subjected to indicated treatments (**h**). TRCs from *HERC6*-knockout HONE1 cells or parental cells and bulk HONE1 cells were subjected to indicated treatments (**i**). RSL3, 15 µM; radiation (IR), 8 Gy; cisplatin (Cis), 20 µM. The percentage of dead cells was measured. **j**, HONE1 cells were implanted subcutaneously into female BALB/c nude mice and were locally exposed to radiotherapy (8 Gy, arrow) or not. Mice were provided with Stattic (3 mg kg^−1^, every 2 d, starting from ‘arrowhead’ until the endpoint) or not. Tumor volumes for each group are shown (*n* = 5). *P* = 0.00002 and 0.0075. Data are shown as mean ± s.d. (**b**,**f**,**h**,**i**) or mean ± s.e.m. (**j**); one-way ANOVA (**b**,**f**,**h**,**i**) or two-way ANOVA (**j**). *n* = 3 independent experiments. One of three experiments is shown (**a**,**c**–**e**,**g**) CHX, cycloheximide. Source data

**Extended Data Fig. 8:**
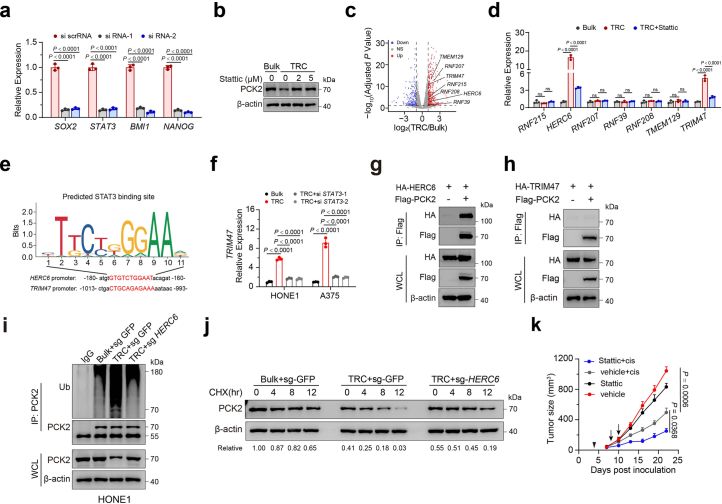
STAT3 promotes PCK2 degradation via HERC6- dependent proteasome pathway in TRCs. **a**, The indicated mRNA expression was analyzed by qRT-PCR for genes knockdown efficiency assessment in HONE1 cells. *P* = 0.0000005, 0.0000006, 0.0000005, 0.00000058, 0.00000028, 0.00000016, 0.000000025 and 0.000000019. **b**, Immunoblots showing the expression of PCK2 in HONE1 cells treated with or without STAT3 inhibitor (Stattic). **c**, Volcano plot showing upregulation of seven E3 ligases in HONE1 TRCs. **d**, Quantitative RT-PCR analysis of the relative mRNA expression of indicated genes in HONE1 TRCs and bulk cells treated with or without Stattic. *P* = 0.168, 0.168, 0.00000057, 0.0000016, 0.267, 0.987, 0.999, 0.818, 0.536, 0.872, 0.581, 0.913, 0.000006, 0.00002. **e**, STAT3-binding elements on the promoters of *HERC6* and *TRIM47* genes were predicted by JASPAR. **f**, qRT-PCR analysis of *TRIM47* expression in HONE1 or A375 TRCs and bulk cells with *STAT3* knockdown. *P* = 0.0000000047, 0.000000017, 0.000000014, 0.00000011, 0.00000034 and 0.00000029. **g**, Immunoprecipitation and immunoblot analysis showing the binding of PCK2 to HERC6 in HEK293T cells transfected with Flag- PCK2 and HA-HERC6. **h**, Immunoprecipitation and immunoblot analysis showing that PCK2 does not bind to TRIM47 in HEK293T cells that were transfected with Flag-PCK2 and HA-TRIM47. **i**, *HERC6*-knockout HONE1 cells (sg *HERC6*) or parental HONE1 cells (sg GFP) were cultured in 3D fibrin gels, after which the cell lysates were subjected to immunoprecipitation with anti-PCK2 antibody and immunoblotting with anti-ubiquitin (Ub) antibody. **j**, Immunoblots showed that knocking out *HERC6* stabilized PCK2 protein in HONE1 TRCs with CHX treatment for indicated times. **k**, The HONE1 cells were implanted subcutaneously into female BALB/c nude mice to construct xenograft growth models. Mice were provided with Stattic (3 mg/kg, every two days, starting from ‘arrowhead’, until the endpoint) or not. Cisplatin or vehicle was intraperitoneally injected (arrow). Tumors volumes for each group were shown (n = 5). Data are shown as mean ± SD (a, d, f) or mean ± SEM (k), one- way ANOVA (a, d, f) or two-way ANOVA (k). n = 3 independent experiments. One of three experiments is shown (b, g-j). Source data

To identify the key transcription factor that regulates PCK2 expression, we screened transcription factors identified in our previous studies^20,43^ that played critical roles in TRCs (Extended Data Fig. 8a). We found that only knocking down *STAT3* abolished PCK2 downregulation in TRCs, whereas knocking down of *SOX2*, *BMI-1* and *NANOG* showed little effect (Fig. 5a). Through specific STAT3 inhibitor (Stattic), we further confirmed the role of STAT3 in regulating PCK2 (Extended Data Fig. 8b). However, in the presence or absence of *STAT3* knockdown, no significant change was observed in PCK2 mRNA level between TRCs and bulk tumor cells (Fig. 5b), indicating that STAT3 did not regulate PCK2 through transcriptional level. Interestingly, we found that PCK2 showed a decreased half-life in HONE1 TRCs, which could be prolonged under Stattic treatment (Fig. 5c). More importantly, the degradation of PCK2 in TRCs could be blocked by proteasome inhibitors (MG132; carfilzomib) but not lysosome inhibitors (Baf A1; chloroquine), indicating that TRCs regulate PCK2 protein levels depending on proteasome (Fig. 5d and Supplementary Fig. 7a). Ubiquitination is a key step for the proteasome-dependent degradation of protein^44^. Consistently, we found that the ubiquitination level of PCK2 was increased in TRCs compared to bulk tumor cells and could be reversed under Stattic treatment (Fig. 5e).

We tested whether STAT3 promoted PCK2 degradation by upregulating key E3 ubiquitin ligases. RNA sequencing analysis showed that seven E3 ubiquitin ligases were upregulated in HONE1 TRCs compared to bulk tumor cells (Extended Data Fig. 8c). Next, we noticed that HERC6 and TRIM47 were upregulated in TRCs and decreased upon Stattic treatment through RT–qPCR (Extended Data Fig. 8d). The promoters of both genes had a conserved STAT3 binding sequence, and *STAT3* knocking down decreased the expression of these two genes (Fig. 5f and Extended Data Fig. 8e,f). However, immunoprecipitation and immunoblotting analysis showed that PCK2 could interact only with HERC6 but not with TRIM47 (Extended Data Fig. 8g,h). Consistently, knocking down of *HERC6*, but not *TRIM47*, increased PCK2 expression in HONE1 and A375 TRCs (Fig. 5g and Supplementary Fig. 7b). We also observed that knockout of *HERC6* decreased the ubiquitination level and prolonged the half-life of PCK2 in both HONE1 and A375 TRCs (Extended Data Fig. 8i,j and Supplementary Fig. 7c–e).

We then verified the roles of STAT3 and HERC6 in ferroptosis. Compared to WT TRCs, both *STAT3-*knocking-out TRCs and *HERC6*-knocking-out TRCs showed increased cell death under RSL3, radiation or cisplatin treatment (Fig. 5h,i and Supplementary Fig. 7f–h). In addition, STAT3 inhibitor treatment in HONE1 tumor-bearing BALB/c nu/nu mice synergistically increased the effect of radiation or cisplatin, as evidenced by reduced tumor volumes (Fig. 5j and Extended Data Fig. 8k). Together, these data suggest that STAT3 activation is critical in regulating PCK2 expression and, thus, leads to decreased ferroptosis sensitivity of TRCs.

### PCK2-pACSL4(T679) promotes ferroptosis in vivo

**Fig. 6:**
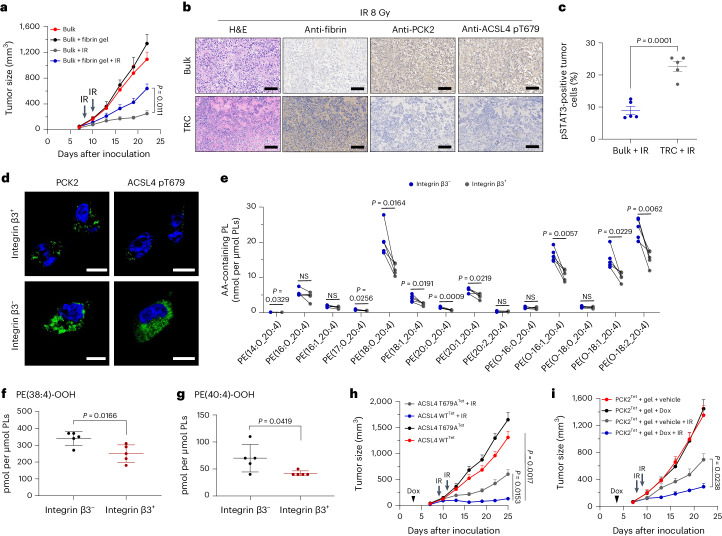
PCK2-ACSL4(T679) phosphorylation promotes ferroptosis in vivo*.* **a**, HONE1 cells with or without fibrin gel were implanted subcutaneously into female BALB/c nude mice to construct xenograft growth models and were exposed to radiotherapy (8 Gy, arrow). Tumor volumes for each group are shown (*n* = 5). **b**, Hematoxylin and eosin (H&E) staining and IHC of fibrin, PCK2 and ACSL4 pT679 showing the presence of fibrin gel and the level of PCK2 and ACSL4 pT679 in TRC tumors and bulk tumors treated with radiotherapy. Images are representative of *n* = 5 images. Scale bars, 100 µm. **c**, Flow cytometric analysis of the proportion of EpCAM^+^pSTAT3^+^ in TRC tumors and bulk tumors treated with radiotherapy. *n* = 5. **d**, Representative immunofluorescence images of PCK2 and ACSL4 pT679 in EpCAM^+^integrin β3^+^ and EpCAM^+^integrin β3^−^ cells. Scale bars, 10 µm. Images are representative of *n* = 10 images. **e**, The content of esterified AA (C20:4)-PE molecular species in integrin β3^+^ and integrin β3^−^ tumor cells isolated from radiation-treated HONE1 tumors. *n* = 5. **f**,**g**, The content of PE (38:4)-OOH (**f**) and PE (40:4)-OOH (**g**) in integrin β3^+^ and integrin β3^−^ tumor cells isolated from radiation-treated HONE1 tumors. *n* = 5. **h**, HONE1 cells stably transfected with indicated plasmids were implanted subcutaneously into female BALB/c nude mice to construct xenograft growth models. Indicated groups were exposed to radiotherapy (8 Gy, arrow). All groups were provided with a Dox drink (100 mg kg^−1^, intragastrical administration, every 2 d, starting from ‘arrowhead’ until the endpoint). Tumor volumes for each group are shown (*n* = 5). **i**, PCK2^Tet^ HONE1 cells with or without fibrin gel were implanted subcutaneously into female BALB/c nude mice to construct xenograft growth models. Indicated groups were exposed to radiotherapy (8 Gy, arrow). Mice were provided with a Dox drink (100 mg kg^−1^, intragastrical administration, every 2 d, starting from ‘arrowhead’ until the endpoint) or a normal drink. Tumor volumes for each group are shown (*n* = 5). Data are shown as mean ± s.e.m. (**a**,**h**,**i**) or mean ± s.d. (**c**,**e**–**g**); paired two-tailed *t*-test (**e**), unpaired two-tailed *t*-test (**c**,**f**,**g**) or two-way ANOVA (**a**,**h**,**i**). Source data

**Extended Data Fig. 9:**
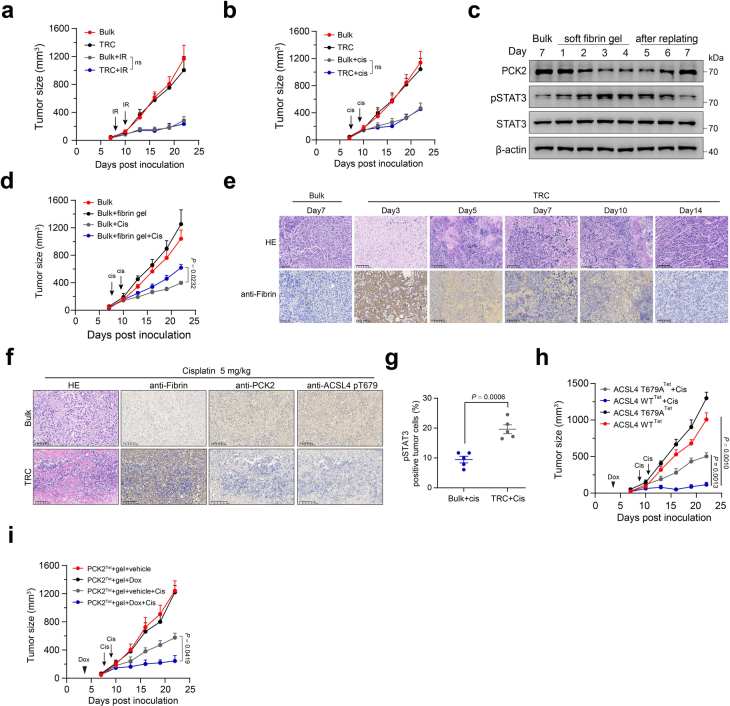
PCK2-ACSL4(T679) phosphorylation axis promotes ferroptosis *in vivo.* **a-b**, The TRCs and bulk tumor cells from HONE1 cells were implanted subcutaneously into female BALB/c nude mice to construct xenograft growth models and exposed to radiotherapy (8 Gy, arrow) **(a)** or cisplatin (5 mg/kg, arrow) **(b)**. Tumor volume for each group were shown (n = 5). **c**, Immunoblots showing the expression of PCK2, pSTAT3 and STAT3 in bulk tumor cells and TRCs from HONE1 cells in indicated conditions. One of three experiments is shown. **d**, The HONE1 cells with or without fibrin gel were implanted subcutaneously into female BALB/c nude mice to construct xenograft growth models and exposed to cisplatin (arrow). Tumor volumes for each group were shown (n = 5). **e**, H&E staining and Immunohistochemistry of fibrin showing the presence of fibrin gel in TRC tumors. Images are representative of n = 5 images. **f**, H&E staining and immunohistochemistry of fibrin, PCK2 and ACSL4 pT679 showing the presence of fibrin gel and the level of PCK2 and ACSL4 pT679 in TRC tumors and bulk tumors treated with cisplatin. Images are representative of n = 5 images. **g**, Flow cytometric analysis of the proportion of EpCAM^+^ pSTAT3^+^ in TRC tumors and bulk tumors treated with cisplatin. n = 5. **h-i**, The HONE1 cells stably transfected with indicated plasmids **(h)** or PCK2^Tet^ HONE1 cells with or without fibrin gel **(i)** were implanted subcutaneously into female BALB/c nude mice to construct xenograft growth models. Indicated groups were exposed to cisplatin (arrow). Mice were provided with a Dox drink (100 mg/kg, every two days, starting from ‘arrowhead’, until the endpoint) or a normal drink. Tumor volumes for each group were shown (n = 5). Data are shown as mean ± SEM (a-b, d, h-i) or mean ± SD (g), unpaired two-tailed *t*-test (g) or two-way ANOVA (a-b, d, h-i). Source data

Next, we validated our experimental findings in in vivo models. We harvested and digested HONE1 TRC spheroids to obtain single-cell suspension and then generated subcutaneous tumor xenograft models in BALB/c nu/nu mice. Compared to the bulk tumor group, the TRC tumors exhibited no difference in tumor growth after radiation or cisplatin (Extended Data Fig. 9a,b). This could be explained by our observation that the stem-cell-like characteristics of TRCs can be maintained for only 2–3 d without the aid of soft fibrin gel (Extended Data Fig. 9c). Thus, we mixed HONE1 cells with or without fibrinogen and thrombin^45^, and we immediately performed subcutaneous tumor injection to generate an in-vitro-like TRC culture microenvironment and the corresponding controls. We found no differences in tumor formation time between the bulk tumor group and the TRC tumor group, whereas TRC tumors exhibited obviously decreased sensitivity to radiation or chemotherapy by showing larger tumor volumes than bulk tumors (Fig. 6a and Extended Data Fig. 9d). To examine the maintenance of fibrin gel within tumors, we collected TRC tumors on the 3rd, 5th, 7th, 10th and 14th day after subcutaneous injection. Through fibrin staining, we observed that the fibrin gel could persist in tumor bed for at least 10 d (Extended Data Fig. 9e). The expression of PCK2 and ACSL4 pT679 remained lower and pSTAT3 expression remained higher in TRC tumors collected on the 11th day (Fig. 6b,c, Extended Data Fig. 9f,g and Supplementary Fig. 8a). Integrin β3 was used to identify TRCs in in vitro experiments, because it was one of the stem-cell-like cancer cell markers and was critical in the maintenance of TRCs^45^. Consistently, we found that integrin β3^+^ tumor cells isolated from HONE1 tumors exhibited lower levels of pACSL4(T679) and PCK2 than those of integrin β3^−^ tumor cells (Fig. 6d and Supplementary Fig. 8b). Integrin β3^+^ tumor cells isolated from irradiated HONE1 tumors also showed less AA-containing PE species and di-oxygenated arachidonoyl and adrenoyl-PE species (Fig. 6e–g).

To further determined the in vivo effect of ACSL4 T679 phosphorylation, we constructed tumor-bearing mice by subcutaneously injecting ACSL4^TetOn^-*ACSL4*^−/−^ (ACSL4 WT^Tet^) HONE1 cells and ACSL4-T679A^TetOn^-*ACSL4*^−/−^ (ACSL4 T679A^Tet^) HONE1 cells, followed by gene expression induction with doxycycline (Dox). We found that ACSL4-T679A overexpression tumors were larger than those of ACSL4 WT overexpression tumors after radiation or cisplatin treatment (Fig. 6h and Extended Data Fig. 9h). To illuminate the role of PCK2 in determining the sensitivity of TRC tumors to radiation or chemotherapy, we mixed PCK2^TetOn^-*PCK2*^−/−^ (PCK2^Tet^) HONE1 cells with fibrinogen and thrombin to generate PCK2^Tet^ TRC tumors grown in fibrin gel. The PCK2^Tet^ TRC tumors treated with Dox showed significantly reduced tumor volume than tumors without Dox treatment after radiation or chemotherapy treatment (Fig. 6i and Extended Data Fig. 9i). Taken together, our in vivo experiments suggest that the PCK2-pACSL4(T679) axis plays a key role in ferroptosis sensitivity.

**Extended Data Fig. 10:**
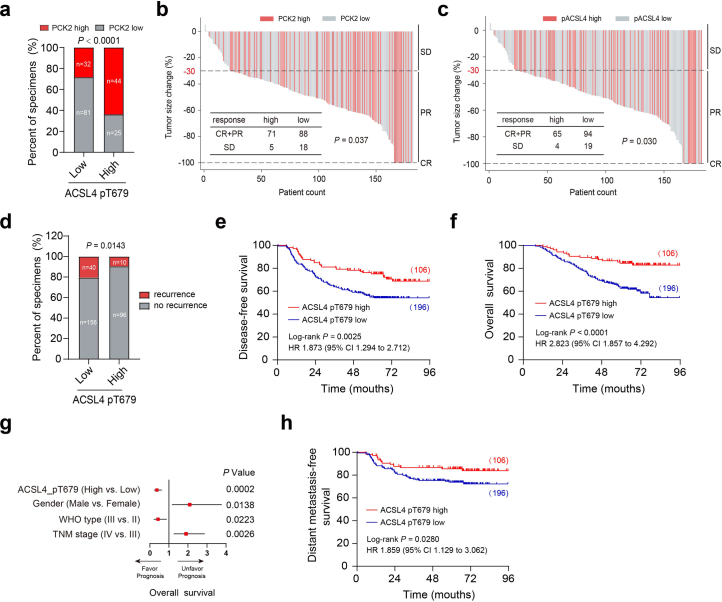
PCK2-ACSL4(T679) phosphorylation is related to better therapy response in NPC. **a**, Correlation analysis of PCK2 and ACSL4 pT679 expression detected by IHC. The *P* value was determined using the two- tailed *χ*^*2*^ test. **b-c**, The PCK2 **(b)** and ACSL4 pT679 **(c)** expression in pre-treatment biopsies are positively related to clinical response of NPC patients to platinum-based induction chemotherapy. CR, complete response, n = 17; PR, partial response, n = 142; SD, stable disease, n = 23. The *P* value was determined using the two-tailed *χ*^*2*^ test. **d**, Correlations of locoregional recurrence status with the level of ACSL4 pT679 detected by IHC. The *P* value was determined using the two-tailed *χ*^*2*^ test. **e-f**, Kaplan-Meier analysis of disease-free survival **(h)** and overall survival **(i)** according to the ACSL4 pT679 expression levels. The *P* values in **h-i** were determined using the log-rank test. *P* = 0.0025 and 0.000049. **g**, Forest plots showing the results of multivariate Cox regression analysis, indicated that ACSL4 pT679 expression is a significant prognostic indicator for overall survival in NPC patients (Cox proportional-hazards model). pACSL4(T679) level (High vs. Low: HR = 0.219–0.631), gender (Male vs. Female: HR = 1.163–3.777), WHO type (III vs. II: HR = 0.206–0.887) and TNM stage (IV vs. III: HR = 1.252–2.886). **h**, Kaplan-Meier analysis of distant metastasis-free survival according to the ACSL4 pT679 expression levels. The *P* value was determined using the log-rank test. Source data

Finally, we validated our findings in clinical patient samples. In a retrospective induction chemotherapy cohort of patients with NPC from our center (*n* = 182; Sun Yat-sen University Cancer Center (SYSUCC) NPC cohort-1) (Supplementary Table 1), we performed IHC staining of samples with anti-pACSL4(T679) or anti-PCK2 antibodies (Supplementary Fig. 9a), and we observed a strong correlation between the signal intensity of PCK2 staining and that of pACSL4(T679) staining (Extended Data Fig. 10a). Combining treatment response with IHC staining results, we found that patients with higher PCK2 expression or higher pACSL4(T679) levels showed better response to chemotherapy (Supplementary Fig. 9b and Extended Data Fig. 10b,c).

In another retrospective radiotherapy cohort of patients with NPC (*n* = 302; SYSUCC NPC cohort-2) (Supplementary Table 2), we performed IHC staining with anti-pACSL4(T679) antibody (Supplementary Fig. 9c), and we found that patients with higher pACSL4(T679) levels correlated with less local recurrence after radiotherapy (Extended Data Fig. 10d). Similarly, Kaplan–Meier analysis showed that higher pACSL4(T679) levels correlated with better disease-free survival and overall survival (Extended Data Fig. 10e,f). Additional multivariate Cox regression analyses identified pACSL4(T679) level, gender, WHO type and TNM stage as independent prognostic indicators for NPC overall survival (Extended Data Fig. 10g and Supplementary Table 3).

More importantly, tumor cells that are less prone to lipid peroxidation are more likely to develop distant metastasis^30^. Consistently, we observed that patients with lower pACSL4(T679) levels correlated with worse distant metastasis-free survival in the radiotherapy cohort (Extended Data Fig. 10h). In public datasets, patients with lower PCK2 expression in the primary tumor of colorectal cancer or hepatocellular carcinoma were more likely to develop distant metastasis (Supplementary Fig. 9d,e). In patients with melanoma or clear cell renal cell carcinoma, the PCK2 expression was also lower in metastatic tumor than in primary tumor (Supplementary Fig. 9f,g). Together, these data suggest that higher PCK2 and pACSL4(T679) expression indicates a better response to radiotherapy and chemotherapy as well as a good survival prognosis and a low likelihood of relapse or distant metastasis in patients.

## Discussion

In this study, we revealed that PCK2 directly interacted with ACSL4 to phosphorylate T679, enhancing its catalytic effect, mediating phospholipid remodeling and increasing cell sensitivity to ferroptosis. Moreover, we also found that the TRCs activated STAT3, which downregulated PCK2 expression, leading to ferroptosis insensitivity, and participated in their tolerance to chemotherapy and radiotherapy. This state was maintained by the extracellular matrix component fibrin through interaction with integrin β3. Furthermore, we confirmed the role of PCK2-ACSL4(T679) phosphorylation in increasing ferroptosis in in vivo tumor models. More importantly, we identified that higher levels of PCK2 and pACSL4(T679) were associated with better response in NPC chemotherapy and radiotherapy cohorts. Collectively, our data further unveiled the close connection between mitochondria and ferroptosis, uncovered the non-canonical function of the metabolic kinase PCK2 as a protein kinase and identified T679 on ACSL4 as an unrecognized core activation site.

Lipid peroxidation is the hallmark of ferroptosis. Ferroptosis occurs when lipid peroxidation exceeds the tolerance of the cell. It is suggested that the intracellular levels of reactive oxygen species (ROS) are not equal to the degree of cellular lipid peroxidation. Mitochondria are the main source of ROS^46^, and they are involved in mediating cysteine deficiency-induced ferroptosis through ROS-associated and non-associated pathways related to tricarboxylic acid cycle, electron transport chain and glutaminolysis^47^. Our data here demonstrated that PCK2 in mitochondria sensitized cells to various ferroptosis inducers (erastin, FIN56, RSL3, FINO2 and IFNγ + AA) of different mechanisms through activation of ACSL4. Moreover, we also noticed that the mitochondrial distribution of ACSL4 was an important condition for this process. Enhanced mitochondrial ACSL4 distribution induced stronger ACSL4 activation and further sensitized cells to ferroptosis. Taken together, our study revealed not only that mitochondria are the place for oxidizing fatty acids to provide energy but also that they participate in the regulation of acyl-CoA production and affect PL remodeling, thereby affecting the sensitivity of cells to ferroptosis rather than merely indirectly by generating ROS or influencing the production of intracellular reducing agents, such as GSH, NADPH and NADH.

Recently, several metabolic enzymes were found to function as protein kinases or phosphatases, directly transmitting signals by interacting with downstream proteins^15^. Our previous study showed that downregulation of PCK2 accelerated the biosynthesis and transportation of citrate from the mitochondria to the cytosol, leading to cytosolic glucose carbon flow via the OAA-malate-pyruvate and acetyl-CoA-fatty acid pathways and balancing biomass-producing glucose intermediates and ATP production in TRCs^48^. Through in vitro kinase activity assays and construction of kinase region mutants, we uncovered a non-canonical function of PCK2 as a protein kinase. Although cytosolic PCK1 has a protein kinase activity and phosphorylates INSIG1/2 to promote celluar lipogenesis^49^, the role of PCK2 as a protein kinase has not been fully elucidated. PCK1 and PCK2 have 63.4% sequence identity in humans, and the composition of protein domains are also highly similar^40,49^. Another recent study also found that PCK2 could translocate to the cytosol under lipopolysaccharide (LPS) stimulation and regulate the phosphorylation of p65 in the NF-κB and AKT pathways^50^, but this study did not explore whether PCK2 directly acts as a protein kinase. From a metabolic viewpoint, PCK2 is the key rate-limiting enzyme of gluconeogenesis, and its expression is regulated by metabolic substrates and various extracellular factors, indicating that glucose metabolism and other regulatory factors may affect lipid remodeling and ferroptosis through PCK2.

ACSL4 mediates cellular PL remodeling by catalyzing the production of PUFA-CoA^10^. It has been shown that PKCβII phosphorylates ACSL4 on T328 to improve its catalytic activity by enhancing ACSL4 dimerization^12^. However, the phosphorylation regulation of ACSL4 is complex and involves various kinases and specific modification sites. Here we showed that T679 phosphorylation on ACSL4 was critical for its activity, and the biological effect of T679 phosphorylation had not been reported. To elucidate the mechanism underlying this effect, we conducted molecular dynamics simulations and found that phosphorylation of T679 had a significant impact on the stability of the catalytic center of ACSL4, the stability of the bond between AA and ACSL4 and the probability of the interaction between AA and CoA within ACSL4. These findings lay the foundation for further proteomic mining of kinases and phosphatases that regulate ACSL4 activity. Overall, our study provides important insights into ACSL4 activity regulation and its potential role in cancer therapy.

## Methods

### Clinical specimens

We collected 182 paraffin-embedded locoregionally advanced NPC specimens between January 2020 and December 2022 (SYSUCC NPC cohort-1) and 302 paraffin-embedded locoregionally advanced NPC specimens between January 2006 and December 2009 (SYSUCC NPC cohort-2) from SYSUCC in Guangzhou, China. None of the patients who provided specimens had been treated with anti-cancer therapies before biopsy. The TNM stages were reclassified according to the 7th edition of the American Joint Committee on Cancer (AJCC) Staging Manual. In SYSUCC NPC cohort-1, all patients underwent platinum-based induction chemotherapy. The clinical features of selected patients are shown in Supplementary Table 1. In SYSUCC NPC cohort-2, all patients underwent radical radiotherapy combined with platinum-based chemotherapy. The clinical features of selected patients are shown in Supplementary Table 2. Our study was approved by the institutional ethical review board of SYSUCC (B2022-569), and the requirement for informed consent was waived by the ethics review boards.

### Cell culture

Human tumor cell lines A375 (melanoma) and HCT116 (colorectal tumor) and HEK293T cells were obtained from the China Center for Type Culture Collection. The human NPC cell lines HONE1 and HK1 were provided by M.-S. Zeng at SYSUCC. All cell lines were cultured in RPMI 1640 medium or DMEM (Invitrogen) supplemented with 10% FBS (Gibco).

### 3D fibrin gel cell culture of tumor cells

TRC culture was conducted according to our previously described method^20,51^. In brief, fibrinogen (Sea Run Holdings) was diluted into 2 mg ml^−1^ with T7 buffer (pH 7.4, 50 mmol l^−1^ Tris, 150 mmol l^−1^ NaCl). Then, a total of 250 μl of cell/fibrinogen mixtures were seeded into each well of a 24-well plate and mixed well with pre-added 5 µl of thrombin (0.1 U μl^−1^; Sea Run Holdings). The cell culture plate was moved into 37 °C cell culture incubator, and then 1 ml of completed culture medium was added into every well after 30 min. After 4 d, Dispase II (Roche) was added into the supernatant of 3D fibrin gels for 10 min at 37 °C. The spheroids were harvested and further digested with 0.25% trypsin for 3 min to obtain a single-cell suspension for the following cultured in conventional two-dimensional (2D) conditions. The indicated drugs (RSL3, FIN56, FINO2 or cisplatin) were added into the medium supernatant for indicated times.

### CRISPR–Cas9-mediated genome editing

To generate indicated knockout cells, optimal sgRNA target sequences (Supplementary Table 4) were designed using Benchling. The annealed guide RNA oligonucleotides were inserted into a PX458 vector (Addgene) digested with the BbsI restriction enzyme. Cells were seeded at 60% confluence, followed by transfection of sgRNAs (1 μg). Ten hours after transfection, cells were trypsinized and sorted for GFP-positive cells. The cells were diluted for single cells and seeded into 96-well plates. The knockout efficiency was validated by western blot.

### Lentivirus-mediated gene transfer

HEK293T cells were co-transfected with pSin-EF2-Puro-based constructed vector, psPAX2 and pMD2.G. Eight hours after transfection, the culture medium was changed to UltraCULTURE medium (Lonza). Forty-eight hours later, the cell virus supernatant was harvested, filtered and used to infect indicated tumor cell lines at a multiplicity of infection (MOI) of 100 overnight. Western blotting assays were conducted to check the transfection efficiency.

### RT–qPCR

Total RNA from cultured cells and clinical samples was isolated with TRIzol reagent (Invitrogen). Total RNA was reverse transcribed using random primers and M-MLV reverse transcriptase (Promega). Complementary DNA was produced using random primers and M-MLV reverse transcriptase (Promega). RT–qPCR was performed using SYBR Green PCR Master Mix (Applied Biosystems) and a CFX96 Touch sequence detection system (Bio-Rad, CFX96). Relative gene expression was calculated by the 2^−ΔΔCT^ equation with GAPDH as an internal control. All the experiments were performed in triplicate, and the primer sequences are shown in Supplementary Table 4.

### Immunoprecipitation

Cells were lysed on ice with immunoprecipitation lysis buffer supplemented with protease and phosphatase inhibitors. The lysates were immunoprecipitated with the indicated antibodies overnight at 4 °C. Pierce Protein A/G Magnetic Beads (Thermo Fisher Scientific) were used to capture the immune complexes at room temperature for 1 h, which were washed with immunoprecipitation wash buffer and then subjected to immunoblotting analysis.

### Immunoblotting analysis

Total protein was obtained using RIPA buffer (Beyotime Biotechnology) containing EDTA-free Protease Inhibitor Cocktail (Beyotime Biotechnology). Total protein was separated by SDS–PAGE (GenScript) and transferred to nitrocellulose filter membranes. The membranes were blocked and incubated with primary antibodies (Supplementary Table 5) overnight at 4 °C. Peroxidase-conjugated secondary antibody was used, and the antigen–antibody reaction was visualized by enhanced chemiluminescence assay (Thermo Fisher Scientific).

### Immunofluorescence analysis

For immunofluorescent staining, the cells were fixed with 4% paraformaldehyde for 15 min at room temperature and washed with PBS three times. Then, the cells were permeabilized with 0.1% Triton X-100 in PBS for 15 min and washed with PBST three times. After blocking and incubating with primary antibodies (Supplementary Table 5), the cells were incubated with Alexa Fluor-conjugated secondary antibodies (Invitrogen) for 1 h at room temperature. DAPI was used to counterstain the nuclei, and images were obtained using a confocal laser scanning microscope (LSM 880, Zeiss).

### IHC analysis

Paraffin-embedded samples were sectioned at 3-μm thickness. A pressure cooker performed antigen retrieval for 15–20 min in 0.01 M citrate buffer (pH 6.0). Specimens were incubated with dilutional primary antibodies (Supplementary Table 5) overnight at 4 °C, and the immunodetection was performed on the next day using DAB (Dako) according to the manufacturer’s instructions. Images were obtained with an AxioVision Rel.4.6 computerized image analysis system (Zeiss). All sections were scored by two experienced pathologists according to the immunoreactive score (IRS) system. The staining intensity score was defined as follows: 0, negative staining; 1, weak staining; 2, moderate staining and 3, strong staining. The positive rate score was defined as follows: 1, <10%; 2, 10–35%; 3, 35–70% and 4, >70%. The total score of indicated proteins was calculated as the staining intensity score multiplied by the positive rate score.

### Ferroptosis assay

For the cell viability assay, 2,000 cells were plated in replicates in 96-well plates 1 d before adding the indicated drug. Cell viability was assessed 2 d after drug treatment by Cell Counting Kit-8 (TargetMol) and normalized to an untreated control. Curve fitting and calculation of half-maximal inhibitory concentration (IC_50_) values were conducted using GraphPad Prism software.

To estimate ferroptotic cell death, 7-AAD-positive tumor cell percentage was measured after incubating cells with indicated drugs or irradiation through 7-AAD (BioLegend) staining and flow cytometric analysis. The data were collected with CytoFLEX LX and CytExpert 2.4 and analyzed with FlowJo 10 software.

### In vitro kinase assay

Human-activated PCK2-Flag protein was immunoprecipitated and purified using anti-DYKDDDDK magnetic Agarose from HEK293T cells that expressed PCK2-Flag. Human recombinant ACSL4-His (AA552-711, GenScript) protein was incubated with or without PCK2-Flag or λ-phosphatase (P2316S, Beyotime Biotechnology) in 1× kinase buffer (9802, Cell Signaling Technology) supplemented with 200 µM cold GTP (D7380, Beyotime Biotechnology) for 30 min at 30 °C. The reaction of active PCK2 kinase without substrate was carried out under the same conditions as the negative control. The kinase assay was stopped with 10 μl of 5× SDS sample buffer and boiled at 100 °C for 10 min. The ACSL4 Thr679 phosphorylation level was measured using the indicated antibody by immunoblotting analysis.

### Identification of oxidized PLs by LC–MS

Lipids were extracted according to the reported Folch procedure^52^. Global oxidized phospholipidomics was performed as previously described^34,53^. PLs were analyzed by LC–MS using a Dionex UltiMate 3000 LC system coupled with a Q-Exactive mass spectrometer (Thermo Fisher Scientific). Samples were separated on a normal phase column (Luna Silica (2), 3 μm, 150 × 2.0 mm (Phenomenex)) at a flow rate of 0.2 ml min^−1^. The mobile phase consists of 10 mM ammonium formate in isopropanol/hexane/water (285:215:5, v/v/v, solvent A) and isopropanol/hexane/water (285:215:40, v/v/v, solvent B). All solvents were LC–MS grade. The gradient elution program was set as follows: 0 min, 10% B; 23 min, 32%; 32 min, 65%; 35 min, 100%; 70 min, 100%. The column temperature was set at 35 °C. The injection volume was 5 μl. Analysis was performed in negative ion mode. Data were acquired at a resolution of 70,000 for the full MS scan and 17,500 for the MS/MS scan in data-dependent mode. The scan range for MS analysis was 400–1,800 *m/z* with a maximum injection time of 200 ms using one microscan. A maximum injection time of 500 ms was used for MS/MS analysis with collision energy set to 24 eV. An isolation window of 1.0 Da was set for the MS/MS scans.

Analysis of raw LC–MS data was performed using MZmine v.2.5.3 (ref. ^54^) with an in-house-generated analysis workflow and database. Peaks with a signal-to-noise ratio of more than 3 were searched and identified against an oxidized PL database. Values for *m/z* were matched within 5 ppm to identify the lipid species, further filtered by retention time and confirmed by MS/MS analysis with the fragments used for their identification (https://www.lipidmaps.org/). Deuterated PLs (Avanti Polar Lipids) were used as internal standards. PLs were quantified from full-scan LC–MS spectra with ratiometric comparison to the pre-selected internal standard using a corresponding standard curve for each PL class.

### Analysis of AA-d8-containing PLs by LC–MS

Total lipids were separated on a reverse-phase column (Acquity HSS T3, 1.8 μm, 100 × 2.1 mm, Waters) at a flow rate set at 0.3 ml min^−1^. The mobile phase consists of 10 mM ammonium formate in water/acetonitrile (50:50, v/v, solvent A) and isopropanol/acetonitrile (90:10, v/v, solvent B). The gradient elution program was set as follows: 0 min, 30% B; 5 min, 43%; 5.1 min, 50%; 14 min, 70%; 14.1 min, 70%; 23 min, 99%; 26 min, 99%. The column temperature was set at 40 °C. MS and MS/MS analyses were performed on a Q-Exactive mass spectrometer (Thermo Fisher Scientific). Analysis was in both positive and negative ion mode (profile) at a resolution of 70,000 for the full MS scan and 17,500 for the MS/MS scan in data-dependent mode. The scan range for MS analysis was 114–1,700 *m/z* with a maximum injection time of 100 ms using one microscan and an AGC target of 1 × 10^5^. A maximum injection time of 50 ms was used for MS/MS analysis with normalized collision energy set at 20%, 30% and 40%. An isolation window of 1.0 Da was set for the MS/MS scans. Analysis of raw LC–MS data and identification of PLs were performed using MS-DIAL.

### Assessment of LPCAT3 activity

LPCAT3 activity assay was conducted in the 96-well plates according to the previously described method^55^. In brief, cell lysates of HONE1 TRCs and bulk tumor cells were prepared in LPCAT3 assay buffer (10 mM Tris-HCL (pH 7.4), 1 mM EDTA, 150 mM NaCl), and total protein concentrations were detected. Then, 100 μg of whole-cell lysates was incubated with or without 10 μM (*R*)-HTS-3 (selectively inhibit the activity of LPCAT3) for 10 min, followed by adding reaction cocktail (20 μM C18:0 lyso-PE, 20 μM AA-CoA, 20 μM DTNB) in a final volume of 100 μl. After incubating at room temperature for 15 min, the absorbance was measured at 412 nm with a Bio-Tek Epoch 2 microplate reader.

### Assessment of ACSL4 activity

For ACSL4 purified protein-based enzyme activity detection, ACSL4-WT protein or ACSL4-T679 mutation protein was purified from HEK293T cells transfected with indicated plasmids and ACSL4 or tumor cells. ACSL4 activity reaction was carried out in a final volume of 100 μl at 37 °C for 8 min or 15 min in the following conditions^56^: 240 μg of purified protein, 5 mM ATP, 100 mM Tris-HCl (pH 7.4), 250 μM coenzyme A, 10 μM AA-d8, 0.03% Triton X-100, 1 μM EDTA, 8 mM MgCl_2_, 5 mM DTT. The reaction was stopped by the addition of acetonitrile (0.6 ml) and full vortex to extract. Centrifugation was performed at 18,400*g* for 30 min at 4 °C, and the upper liquid was collected to be dried by nitrogen. Then, 200 μl of 50% methanol was added to fully dissolve the sample. Finally, centrifugation was performed at 18,400*g* for 20 min at 4 °C, and the upper liquid was taken for LC–MS detection.

For whole-cell-lysate-based ACSL4 enzyme activity detection between HONE1 TRCs and bulk tumor cells, 80 μg of whole-cell lysates were incubated with or without 20 μM rosiglitazone (selectively inhibit the activity of ACSL4) for 10 min, followed by adding reaction cocktail (5 mM ATP, 100 mM Tris-HCl (pH 7.4), 250 μM coenzyme A, 10 μM AA-d8, 0.03% Triton X-100, 1 μM EDTA, 8 mM MgCl_2_, 5 mM DTT) in a final volume of 100 μl. Enzyme activity reaction was performed as described above, followed by LC–MS detection. The ACSL4 enzyme conversion rate was calculated by the conversion rate of whole-cell lysates without rosiglitazone (includes all ACS activity) group minus the conversion rate of whole-cell lysates with rosiglitazone (includes ACS activity except ACSL4) group to exclude the enzymatic conversion to AA-d8-CoA of other ACSs in cell lysates.

AA-d8-CoA was analyzed by LC–MS using a Dionex UltiMate 3000 LC system coupled with a Q-Exactive mass spectrometer (Thermo Fisher Scientific) using a reverse-phase column (Acquity HSS T3, 1.8 μm, 100 × 2.1 mm, Waters). The mobile phase consists of 5 mM ammonium formate and 0.01% ammonia in water/acetonitrile (85:15, v/v, solvent A) and water/acetonitrile (10:90, v/v, solvent B). The gradient elution program was set as follows: 0 min, 50% B; 5 min, 100%. The column temperature was set at 40 °C. The injection volume was 2 μl. Spectra were acquired in both positive and negative ion mode at a resolution of 70,000 for the full MS scan and 17,500 for the MS/MS scan in data-dependent mode. The capillary spray voltage was set at 3.0 kV and −2.8 kV, respectively, and the capillary temperature was 350 °C. The S-lens RF level was set to 60. AA-d8-CoA was identified by comparing the retention time of AA-CoA and further confirmed by MS/MS spectra. Analytical data were acquired and analyzed using Xcalibur v.4.2 Quan Browser (Thermo Fisher Scientific).

### MST for ACSL4

GFP-fused ACSL4-WT or ACSL4-T679A mutation plasmid was transfected into HEK293T cells, and then the cell lysates were collected and diluted tenfold in PBST to provide an optimal level of fluorescence for the MST experiment. Fatty acids were dissolved in DMSO at 160 mM and diluted in PBS supplemented with 0.5% Tween 20 and 5% BSA in a series of 16 1:1 dilutions, producing ligand concentrations ranging from 122 nM to 20 mM. Each ligand dilution was mixed with one volume of cell lysate. After 10-min incubation at room temperature, the samples were loaded into standard Monolith NT.115 Capillaries (NanoTemper Technologies). MST was measured using a Monolith NT.115 instrument (NanoTemper Technologies). Data of three independently pipetted measurements were fitted with a nonlinear regression model in GraphPad Prism v.8.0.

### Molecular dynamics simulations

The 3D structure of the ACSL4 protein predicted by the AlphaFold2 package was selected to build the ACSL4 WT model or the ACSL4 phosphorylation model. Three crystal structures of human ACS medium-chain family member 2A (ref. ^57^) from the Protein Data Bank (PDB) (PDB codes: 3C5E, 3EQ6 and 2WD9) were selected to construct the WT ACSL4 model with the substrates of ATP, CoA and AA bound by the Molecular Operating Environment (MOE2020) program. The constructed WT model was employed to further construct the phosphorylation model by connecting a phosphate group with the hydroxyl of T679 residue in the MOE2020 program.

Before the molecular dynamics simulation, each above-prepared model was first hydrogenated by the Leap program in AMBER20; then neutralized by using the AmberTools package; and finally solvated with a 10-Å buffer distance between the solvent box wall and the nearest solute atoms. The molecular dynamics simulations were performed in AMBER20 by employing the GPU-accelerated pmemd.cuda module. With a target temperature of 300 K and under the periodic boundary condition, about 400-ns NPT molecular dynamics simulations were further performed to produce trajectories. During the entire molecular dynamics simulations, FF14SB force fields were applied to describe the behaviors of the ACSL4 protein and the TIP3P water box, and the general AMBER force field (GAFF) was applied to describe the ATP, CoA and AA. The partial atomic charges of the ATP, CoA, and AA were obtained from restrained electrostatic potential (RESP) charges first computed at the HF/6-31G* level with the Gaussian 16 package and then restricted fitted with the antechamber program in AMBER20 (ref. ^58^).

### Construction of tumor xenotransplantation model

Six-week-old female, specific pathogen-free BALB/c nude mice were purchased from Charles River Laboratories. These animals were maintained in five animals per group on a 12h/12-h light/dark cycle at 20–26 °C with 40–70% humidity and were given ad libitum access to standard food and water. BALB/c nude mice were subcutaneously inoculated with 1 × 10^6^ HONE1 cells or stable infection HONE1 cells. After the diameter of xenograft tumors reached 5 mm, the mice were locally irradiated or treated with cisplatin. Tumor volume was calculated using the following formula: length × width^2^ × 0.5. After indicated days, tumor samples were paraffin embedded for IHC analyses. All experimental protocols were approved by the Institutional Animal Care and Use Committee of Sun Yat-sen University and complied with the Declaration of Helsinki. We did our best to minimize animal suffering. The maximal tumor diameter was 20 mm, as permitted by our ethics committee, and the maximal tumor size was not exceeded in our study.

### Statistics and reproducibility

Data are presented of at least three independent experiments. Statistical analyses were performed using GraphPad Prism v.8 (GraphPad Software), SPSS Statistics v.25 (IBM), FlowJo 10 or R v.4.2.3. Two-tailed unpaired Student’s *t*-test and ANOVA with multiple comparisons were used to calculate *P* values. Time-to-event data were described using Kaplan–Meier curves, and differences in survival were determined using the log-rank test. The chi-square (*χ*^*2*^) test was used to compare clinical characteristics. A multivariable Cox proportional hazards model was used to estimate independent prognostic factors. *P* values less than 0.05 were considered statistically significant. Raw LC–MS data were analyzed with MZmine v.2.5.3 and MS-DIAL. PCA was performed with SIMCA v.13.0 software.

### Reporting summary

Further information on research design is available in the Nature Portfolio Reporting Summary linked to this article.

## Online content

Any methods, additional references, Nature Portfolio reporting summaries, source data, extended data, supplementary information, acknowledgements, peer review information; details of author contributions and competing interests; and statements of data and code availability are available at 10.1038/s41589-024-01612-6.

## Supplementary information

Supplementary Information Supplementary Figs. 1–9, Tables 1–5 and Source Data for Supplementary Figs. 4 and 7.

Reporting Summary

Supplementary Video 1 PC1 motion trajectory of ACSL4 WT model.

Supplementary Video 2 PC2 motion trajectory of ACSL4 WT model.

Supplementary Video 3 PC1 motion trajectory of ACSL4 phosphorylation model.

Supplementary Video 4 PC2 motion trajectory of ACSL4 phosphorylation model.

Supplementary Video 5 Representative conformations of CoA in ACSL4 WT model.

Supplementary Video 6 Representative conformations of CoA in ACSL4 WT model_zoom.

Supplementary Video 7 Representative conformations of CoA in ACSL4 phosphorylation model.

Supplementary Video 8 Representative conformations of CoA in ACSL4 phosphorylation model_zoom.

Supplementary Data Statistical source data for supplementary figures.

## Source data

Source Data Figs. 1–6 Statistical source data.

Source Data Figs. 3–5 Unprocessed western blots.

Source Data Extended Data Figs. 1–10 Statistical source data.

Source Data Extended Data Figs. 1–9 Unprocessed western blots.

## Data Availability

All data generated and analyzed in this study are included in the article and its Supplementary Information files. Tumor metastasis data were obtained from the Gene Expression Omnibus (https://www.ncbi.nlm.nih.gov/geo/; accession numbers GSE87211, GSE45114, GSE15605 and GSE22541). The key raw data have been deposited to Research Data Deposit public platform (https://www.researchdata.org.cn/), with an approval number of RDDB2024135520. Source data are provided with this paper.
