# Supplementary material for: Tumor-repopulating cells evade ferroptosis via PCK2-dependent phospholipid remodeling
Source: Nat Chem Biol. 2024 May 8;20(10):1341–52. doi: 10.1038/s41589-024-01612-6 (PMC11427348; doi:10.1038/s41589-024-01612-6)

nature chemical biology

Article

https://doi.org/10.1038/s41589-024-01612-6

Tumor-repopulating cells evade ferroptosis via PCK2-dependent phospholipid remodeling

In the format provided by the authors and unedited

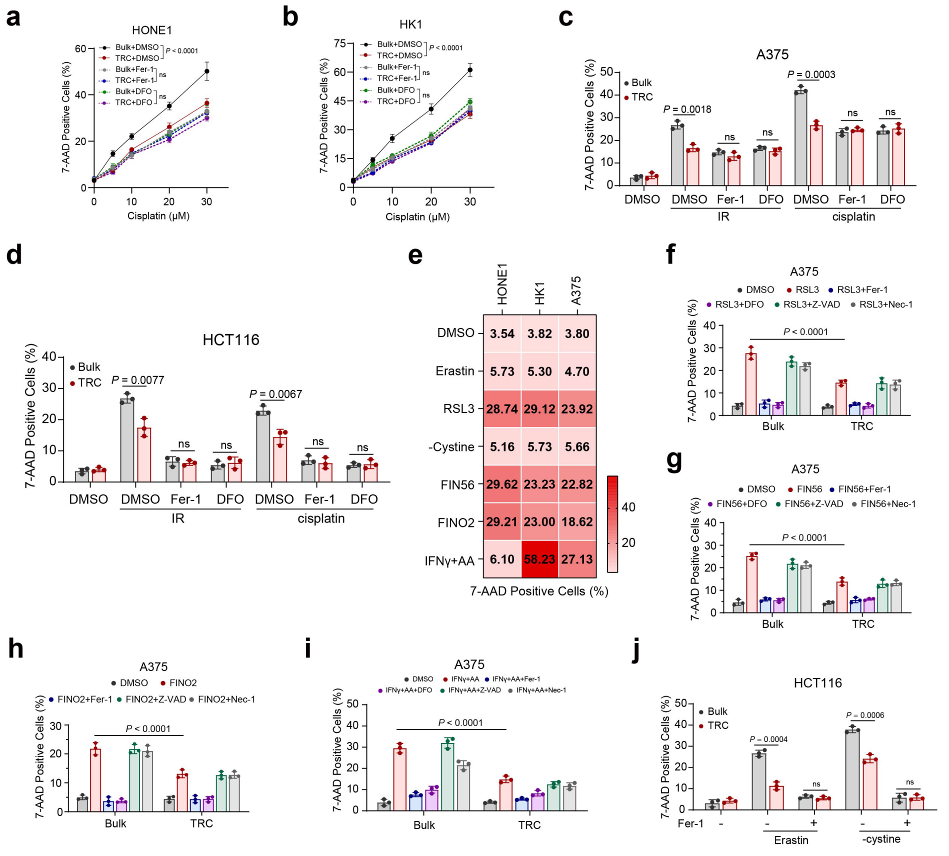

Supplementary Fig.1 TRCs resist radiotherapy and chemotherapy via evading ferroptosis.

a-b, Percentage of dead cells in TRCs and bulk tumor cells from HONE1 cells **(a)** or HK1 cells **(b)** treated with cisplatin in the absence or presence of Fer-1 and DFO. *P* = 1.09E-13 and 2.416E-20.
c-d, Percentage of dead cells in TRCs and bulk tumor cells from A375 **(c)** or HCT116 **(d)** treated with IR or cisplatin in the absence or presence of Fer-1 and DFO.
e, Heatmaps showing the percentage of dead cells of tumor cells treated with indicated ferroptosis inducers. Erastin, 5 μM; RSL3, 10 μM; -Cystine, cystine-free medium; FIN56, 10 μM; FINO2, 10 μM; IFNγ, 30 ng/mL; AA, 25μM.
f-i, Percentage of dead cells in TRCs and bulk tumor cells from A375 treated with indicated inhibitors and RSL3 **(f)**, FIN56 **(g)**, FINO2 **(h)** or IFNγ+AA **(i)**. Fer-1, 1 μM; Z-VAD (Z-VAD-FMK), 10 μM; Nec-1 (Necrostatin-1), 2 μM. *P* = 0.0000000399, 0.00000000607, 0.00000267 and 0.0000000025.
j, Percentage of dead cells in TRCs and bulk tumor cells from HCT116 treated with Fer-1and indicated ferroptosis inducers.

Data are shown as mean ± SD (a-d, f-j), unpaired two-tailed *t*-test (c-d, j), one-way ANOVA (f-i), two-way ANOVA (a-b). n = 3 independent experiments. ns, not significant.

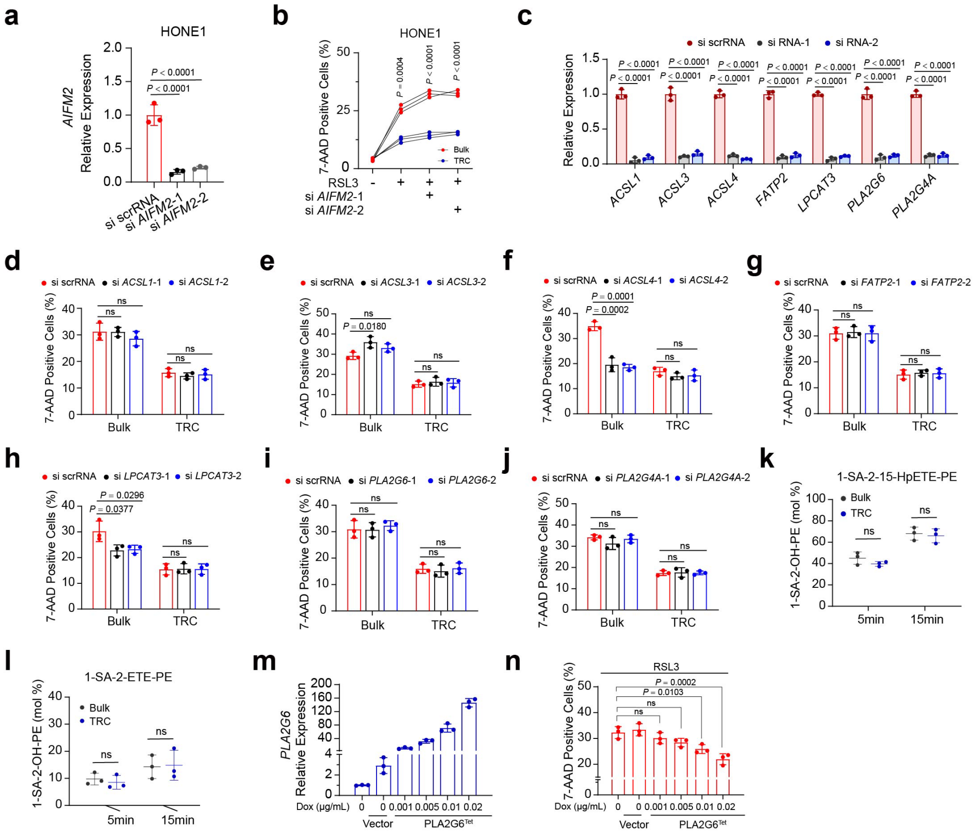

Supplementary Fig.2 Interrogation the involvement of previously identified key genes in mediating resistance to ferroptosis in TRCs.

a, The relative mRNA expression of *AIFM2* in HONE1 cells after transfecting with si-*AIFM2*. *P* = 0.0000734, 0.000114 and 0.69803.

b, Percentage of dead cells in HONE1 TRCs and bulk tumor cells treated with RSL3. si-*AIFM2* were transfected before the treatment of RSL3. *P* = 0.0004, 0.0000827 and 0.00002896.

c, The relative mRNA expression was analyzed by qRT-PCR for indicated gene knockdown efficiency in HONE1 cells. *P* = 0.00000096, 0.0000011, 0.0000031, 0.0000041, 0.00000015, 0.000000095, 0.00000037, 0.00000042, 0.0000000047, 0.0000000084, 0.0000013, 0.0000015, 0.00000021 and 0.00000022.

d-j, TRCs and bulk tumor cells from HONE1 cells were transfected with siRNAs specific for *ACSL1* **(d)**, *ACSL3* **(e)**, *ACSL4* **(f)**, *FATP2* **(g)**, *LPCAT3* **(h)**, *PLA2G6* **(i)** or *PLA2G4A* **(j)** followed by treatment with RSL3. The percentage of dead cells was measured.

k-l, Time course of the formation of 1-SA-2-OH-PE **(k)** or 1-SA-2-OH-PE **(l)** in reactions catalyzed by iPLA2β purified from HONE1 TRCs and bulk cells. 1-SA-2-15-HpETE-PE **(k)** or 1-SA-2-ETE-PE **(l)** was used as substrates.

m, The mRNA expression of *PLA2G6* in HONE1 cells transfected with Dox-inducible PLA2G6 expression vector and parental HONE1 cells.

n, Percentage of dead cells in Dox-inducible PLA2G6 expression HONE1 cells and parental HONE1 cells treated with RSL3.

Data are shown as mean ± SD, unpaired two-tailed *t*-test (b, k-l) or one-way ANOVA (a, c-j, n), n = 3 independent experiments. ns, not significant.

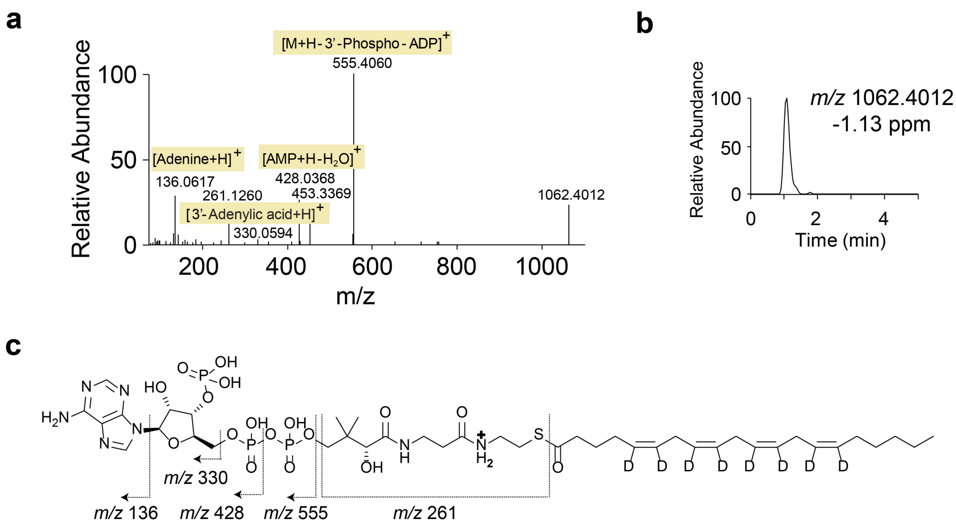

Supplementary Figure 3: LC-MS-based characterization of AA-d8-CoA

a-c, Detection and identification of AA-d8-CoA, the synthetic product of AA-d8 and CoA. MS^2 fragmentation pattern of molecular ions with *m/z* = 1062.4012, corresponding to AA-d8-CoA **(a)**. Base peak chromatogram of molecular ions with *m/z* = 1062.4012, corresponding to AA-d8-CoA **(b)**. Respective structure and fragments formed during MS^2 analysis **(c)**.

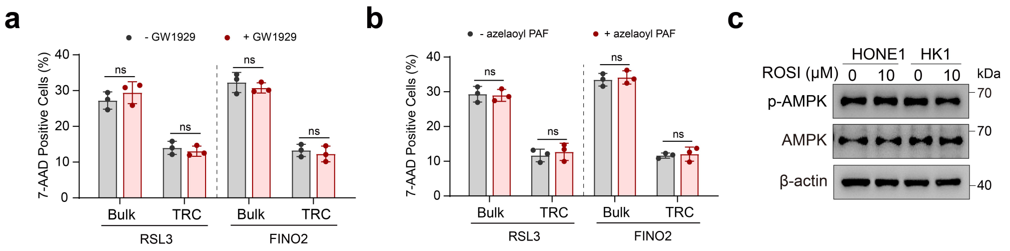

Supplementary Fig.4 Interrogation the possibility that rosiglitazone exerted ferroptosis protective effect through PPARγ or AMPK.

a-b, Percentage of dead cells in TRCs and bulk tumor cells from HONE1 cells co-treated with non-TZD PPARγ agonists GW1929 **(a)** or azelaoyl PAF **(b)** and RSL3 or FINO2.
c, Immunoblots showing the expression of p-AMPK and AMPK in HONE1 and HK1 cells treated with or without ACSL4 inhibitor rosiglitazone.

Data are shown as mean ± SD, unpaired two-tailed *t*-test (a-b). n = 3 independent experiments. One of three experiments is shown (c). ns, not significant.

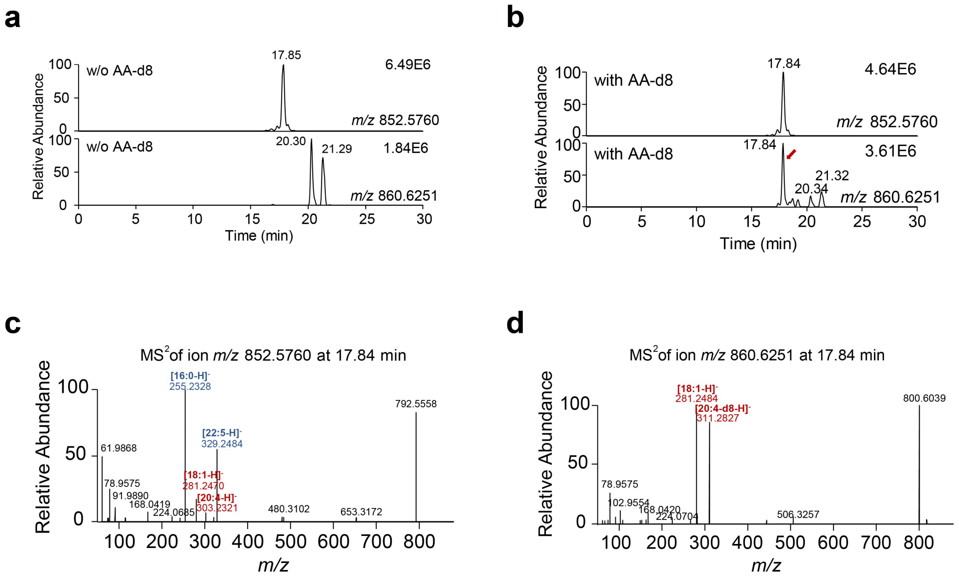

Supplementary Figure 5: LC-MS-based characterization of PL-AA-d8

a, Extract ion currents of $[M+CHOO]^-$ ions of *m/z* 852.5760 (PC (38:5)) and *m/z* 860.6251 (PC (38:5)-d8) in tumor cells without AA-d8 treatment, respectively.

b, Extract ion currents of $[M+CHOO]^-$ ions of *m/z* 852.5760 (PC (38:5)) and *m/z* 860.6251 (PC (38:5)-d8) in tumor cells with AA-d8 treatment, respectively. The red arrow points to the newly added chromatographic peak after AA-d8 treatment.

c, MS^2 of PC (38:5) (ion *m/z* 852.5760 at 17.84 min). Characteristic fragments used for the identification of PC (16:0_22:5) and PC (18:1_20:4) are shown in blue and red, respectively.

d, MS^2 of PC (38:5)-d8 (ion *m/z* 860.6251 at 17.84 min). Characteristic fragments used for the identification of PC (18:1_20:4-d8) are shown in red.

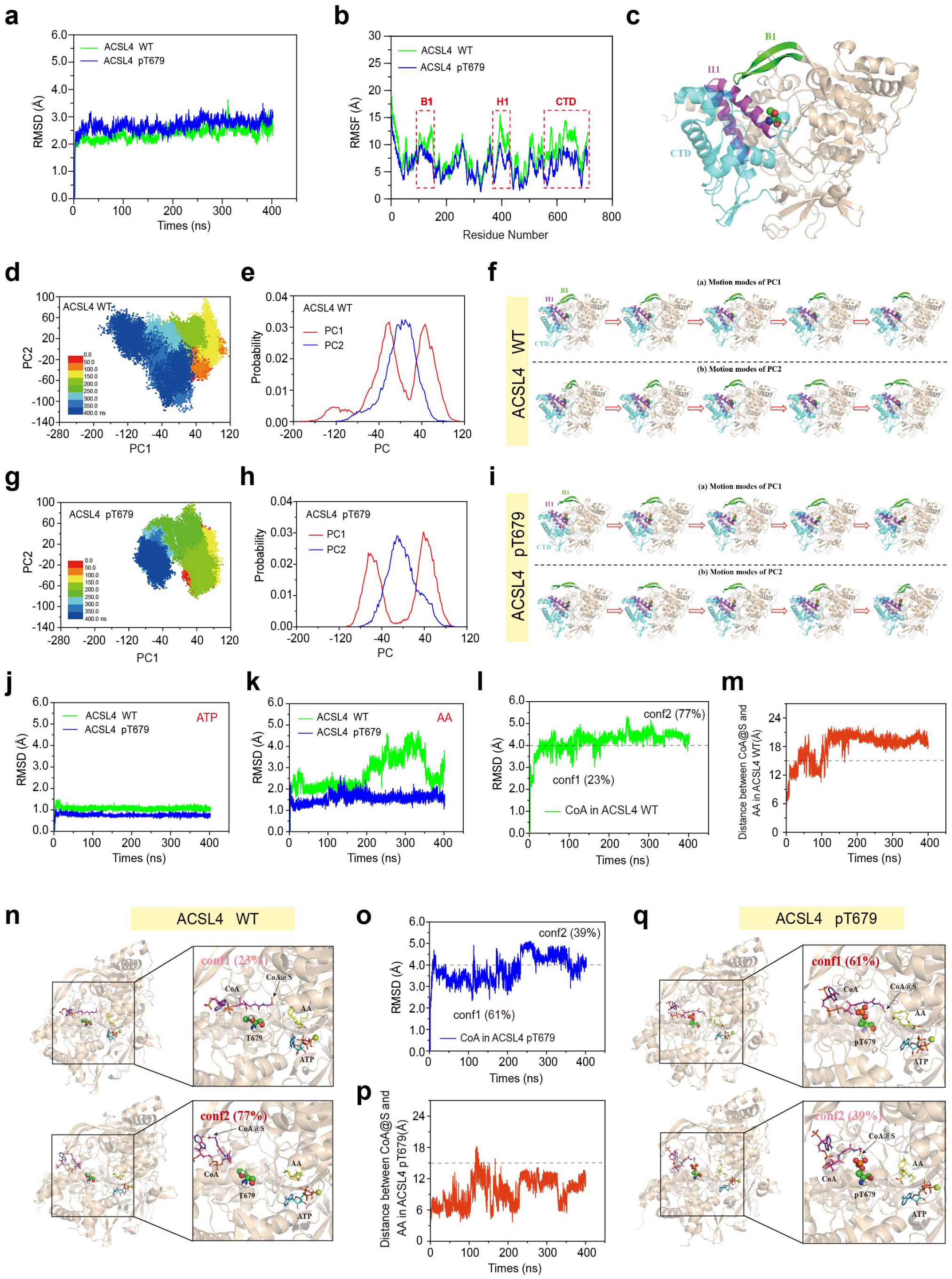

Supplementary Fig.6 Molecular dynamics simulation for ACSL4 T679 phosphorylation.

a, Root-mean-square deviations (RMSD) of the heavy atoms in ACSL4 protein in ACSL4^{WT} model and ACSL4^{pT679} model.

b, Root-mean-square fluctuations (RMSF) of each residue in MD simulations in ACSL4^{WT} model and ACSL4^{pT679} model.

c, The structure of ACSL4 was computationally modeled based on the AlphaFold2 package. B1 (green, residues 103-125), H1 (magenta, residues 375-420) and CTD (cyan, residues 552-711) were used to demonstrate structure differences between ACSL4^{WT} model and ACSL4^{pT679} model.

d-e, The variation trend of principal components (PC) with simulation time **(d)** and histogram of the PC **(e)** in ACSL4^{WT} model during MD simulations.

f, Motion modes of PC1 and PC2 in ACSL4^{WT} model during MD simulation. Top, the PC1 motion trajectory. Bottom, the PC2 motion trajectory.
g-h, The variation trend of PC with simulation time **(g)** and histogram of the PC **(h)** in ACSL4^{pT679} model during MD simulations.
i, Motion modes of the PC1 and PC2 in ACSL4^{pT679} model during MD simulation. Top, the PC1 motion trajectory. Bottom, the PC2 motion trajectory.
j, The heavy atoms RMSD of ATP in the MD simulations in ACSL4^{WT} model and ACSL4^{pT679} model. In both model, the RMSD of ATP rapidly reached a stable state at a very short time, and until the end of the simulations.
k, The heavy atoms RMSD of AA in the MD simulations of in ACSL4^{WT} model and ACSL4^{pT679} model. In ACSL4^{WT} model, the RMSD of AA could not maintain stable in the last 200 ns.
l, The heavy atoms RMSD of substrate CoA. The proportion of "conf1" and "conf2" conformation in ACSL4^{WT} model were 23% and 77%, respectively.
m, The distance change between the S atom of CoA and substrate AA in ACSL4^{WT} model.
n, Representative structures of the substrate CoA in in ACSL4^{WT} model during MD simulations.
o, The heavy atoms RMSD of substrate CoA. The proportion of "conf1" and "conf2" conformation in ACSL4^{pT679} model were 61% and 39%, respectively.
p, The distance change between the S atom of CoA and substrate AA in ACSL4^{pT679} model.
q, Representative structures of the substrate CoA in ACSL4^{pT679} model during MD simulations.

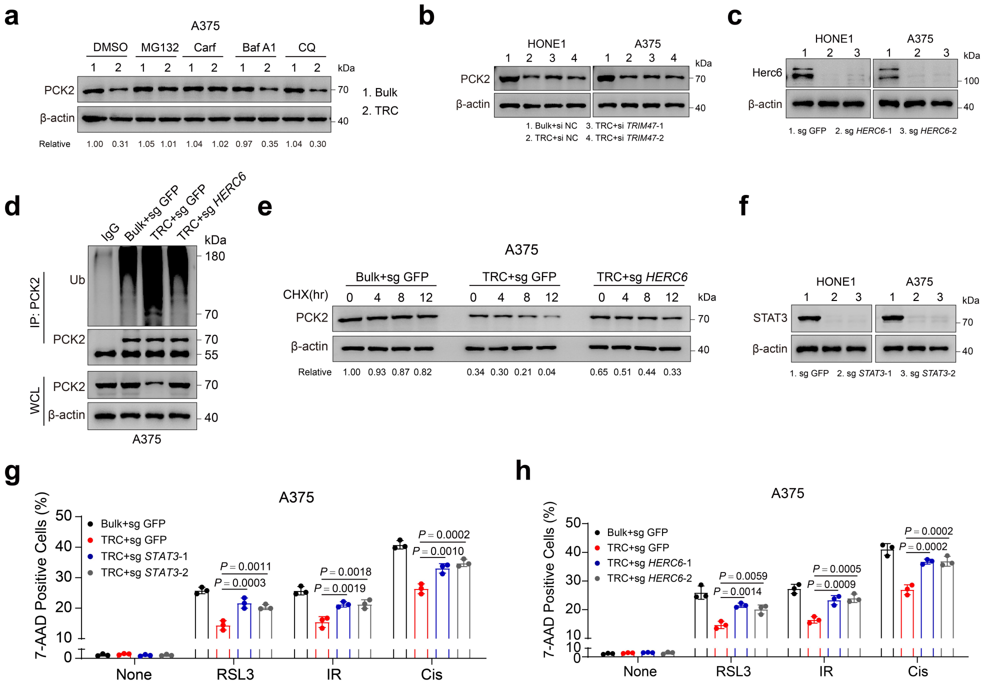

Supplementary Fig.7 STAT3 promotes PCK2 degradation via HERC6-dependent proteasome pathway in TRCs.

a, Immunoblots showing PCK2 protein level in A375 TRCs and bulk tumor cells treated with proteasome inhibitors MG132 (10 μM), carfilzomib (100 nM), or autophagy inhibitor bafilomycin A1 (Baf A1), chloroquine (CQ).

b, Immunoblots showing the expression of PCK2 in TRCs and bulk tumor cells with or without *TRIM47* knockdown (A375).

c, Immunoblots of HERC6 in *HERC6* knockout cells and parental cells.

d, *HERC6*-knockout A375 cells (sg *HERC6*) or parental HONE1 cells (sg GFP) were cultured in 3D fibrin gels, after which the cell lysates were subjected to immunoprecipitation with anti-PCK2 antibody and immunoblotting with anti-ubiquitin (Ub) antibody.

e, Immunoblots showed that knocking out *HERC6* stabilized PCK2 protein in A375 TRCs with CHX treatment for indicated times.

f, Immunoblots of STAT3 in *STAT3*-knockout cells and parental cells.

g, TRCs from *STAT3*-knockout cells and parental cells, and bulk A375 cells were subjected to indicated treatments. The percentage of dead cells was measured.

h, TRCs from *HERC6*-knockout cells and parental cells, and bulk A375 cells were subjected to indicated treatments. The percentage of dead cells was measured.

Data are shown as mean ± SD (g-h), one-way ANOVA (g-h). n = 3 independent experiments. One of three experiments is shown (a-f).

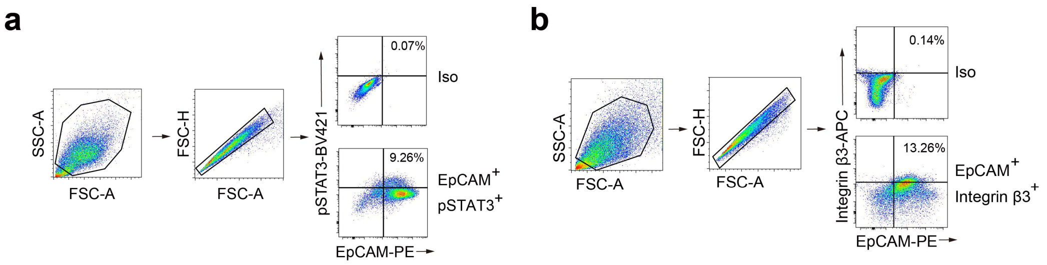

Supplementary Figure 8: FACS gating strategy

a, Representative image of flow cytometric analysis, associated with Fig.6c and Extended Data Fig.9g.

b, $EpCAM^{+}$ integrin $\beta3^{+}$ and $EpCAM^{+}$ integrin $\beta3^{-}$ cells were sorted from HONE1 tumors of BALB/c nude mice. Representative image of flow cytometric analysis, associated with Fig.6d-g.

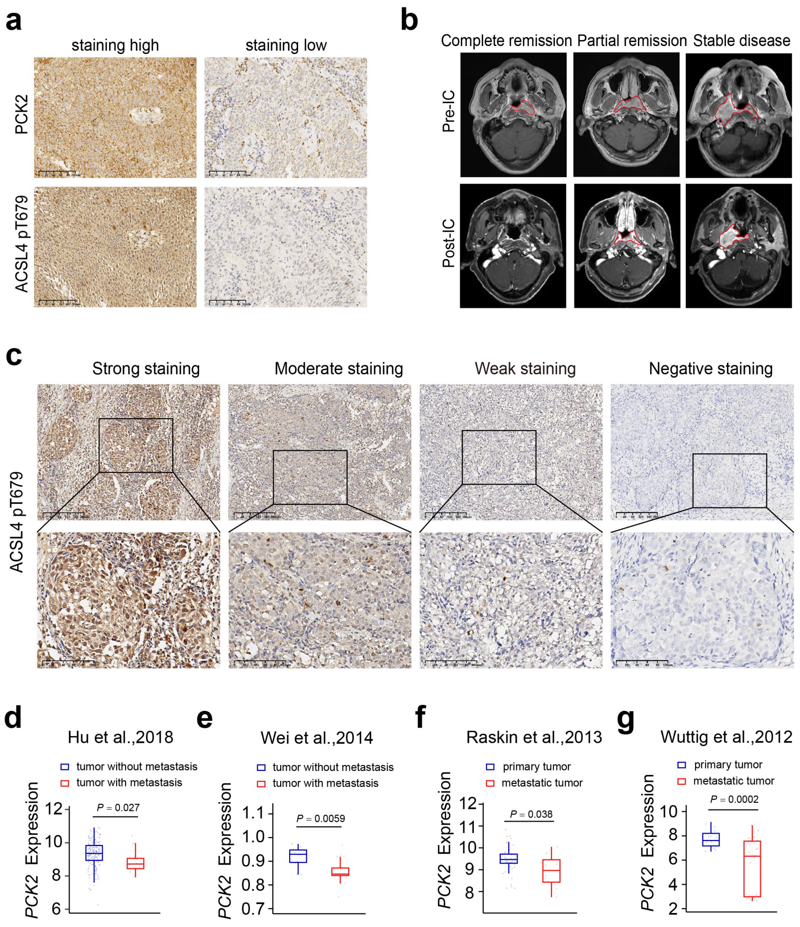

Supplementary Fig.9 PCK2-ACSL4(T679) phosphorylation is related to better therapy response in NPC.

a, Representative images of immunohistochemical staining for PCK2 and ACSL4 pT679 expression which are graded by the staining intensity in 182 NPC tumor tissues.

b, Representative magnetic resonance imaging of SYSUCC NPC cohort-1 before and after platinum-based induction chemotherapy. The red contour depicts radiographically evaluable tumors.

c, Representative images of immunohistochemical staining for ACSL4 pT679 expression which are graded by the staining intensity in 302 NPC tumor tissues.

d, Boxplot illustrated the *PCK2* expression in primary tumor with (n=16) or without metastasis (n=186) in GSE87211 cohort.

e, Boxplot illustrated the *PCK2* expression in primary tumor with (n=11) or without metastasis (n=13) in GSE45114 cohort.

f, Boxplot illustrated the *PCK2* expression in primary tumor tissues (n=46) and metastasis tumor tissues (n=12) in GSE15605 cohort.

g, Boxplot illustrated the *PCK2* expression in primary tumor tissues (n=17) and metastasis tumor tissues (n=44) in GSE22541 cohort.

d-g, Each dot represents a data point for individual patients. The comparison was based on the two-tailed Wilcoxon test.

Supplementary Table 1. Correlations between PCK2 or ACSL4 pT679 expression and clinicopathological characteristics in SYSUCC NPC cohort-1 (n = 182).

Characteristic	No. of patients	PCK2 expression		
		Low expression no.(%)	High expression no.(%)	*P* value
**Age**				
<45 years	71(39.0%)	38(35.8%)	33(43.4%)	0.302
≥45 years	111(61.0%)	68(64.2%)	43(56.6%)	
**Sex**				
Male	128(70.3%)	74(69.8%)	54(71.1%)	0.857
Female	54(29.7%)	32(30.2%)	22(28.9%)	
**WHO type**				
II	4(2.2%)	1(0.9%)	3(3.9%)	0.310
III	178(97.8%)	105(99.1%)	73(96.1%)	
**TNM Stage**				
I-II	8(4.4%)	7(6.6%)	1(1.3%)	0.142
III-IV	174(95.6%)	99(93.4%)	75(98.7%)	
**EBV DNA(copies/ml)**				
<2000	124(68.1%)	77(72.6%)	47(61.8%)	0.123
≥2000	58(31.9%)	29(27.4%)	29(38.2%)	
**VCA-IgA**				
<1:80	22(12.1%)	13(12.3%)	9(11.8%)	0.813
≥1:80	133(73.1%)	75(70.8%)	58(76.3%)	
NA	27(14.8%)	18(17.0%)	9(11.8%)	
**EA-IgA**				
<1:10	12(6.6%)	7(6.6%)	5(6.6%)	0.910
≥1:10	143(78.6%)	81(76.4%)	62(81.6%)	
NA	27(14.8%)	18(17.0%)	9(11.8%)	
**Response**				
CR	17(9.3%)	6(5.7%)	11(14.5%)	0.024
PR	142(78.0%)	82(77.4%)	60(78.0%)	
SD	23(12.6%)	18(17.0%)	5(6.6%)	

CR: complete response; PR: partial response; SD: stable disease

Characteristic	No. of patients	ACSL4 pT679 expression		
		Low expression no.(%)	High expression no.(%)	*P* value
**Age**				
<45 years	71(39.0%)	40(35.4%)	31(44.9%)	0.201
≥45 years	111(61.0%)	73(64.6%)	38(55.1%)	
**Sex**				
Male	128(70.3%)	80(70.8%)	48(69.6%)	0.860
Female	54(29.7%)	33(29.2%)	21(30.4%)	
**WHO type**				
II	4(2.2%)	1(0.9%)	3(4.3%)	0.153
III	178(97.8%)	112(99.1%)	66(95.7%)	
**TNM Stage**				
I-II	8(4.4%)	6(5.3%)	2(2.9%)	0.691
III-IV	174(95.6%)	107(94.7%)	67(97.1%)	
**EBV DNA(copies/ml)**				
<2000	124(68.1%)	79(69.9%)	45(65.2%)	0.510
≥2000	58(31.9%)	34(30.1%)	24(34.8%)	
**VCA-IgA**				
<1:80	22(12.1%)	14(12.4%)	8(11.6%)	0.757
≥1:80	133(73.1%)	80(70.8%)	53(76.8%)	
NA	27(14.8%)	19(16.8%)	8(11.6%)	
**EA-IgA**				
<1:10	12(6.6%)	8(7.1%)	4(5.8%)	0.891
≥1:10	143(78.6%)	86(76.1%)	57(82.6%)	
NA	27(14.8%)	19(16.8%)	8(11.6%)	
**Response**				
CR	17(9.3%)	7(6.2%)	10(14.5%)	0.026
PR	142(78.0%)	87(77.0%)	55(79.7%)	
SD	23(12.6%)	19(16.8%)	4(5.8%)	

CR: complete response; PR: partial response; SD: stable disease

Supplementary Table 2. Correlations between ACSL4 pT679 expression levels and clinical features in SYSUCC NPC cohort-2 (n = 302).

Characteristic	No. of patients (%)	Low expression no.(%)	High expression no.(%)	*P* Value
**Age**		196	106	
≤45 years	158(52.3%)	103(52.6%)	55(51.9%)	0.912
>45 years	144(47.7%)	93(47.4%)	51(48.1%)	
**Sex**				
Male	78(25.8%)	46(23.5%)	32(30.2%)	0.203
Female	224(74.2%)	150(76.5%)	74(69.8%)	
**WHO type**				
II	13(4.3%)	10(5.1%)	3(2.8%)	0.353
III	289(95.7%)	186(94.9%)	103(97.2%)	
**Family history**				
No	259(85.8%)	169(86.2%)	90(84.9%)	0.754
Yes	43(14.2%)	27(13.8%)	16(15.1%)	
**T stage**				
T1-2	50(16.6%)	32(16.3%)	18(17.0%)	0.884
T3-4	252(83.4%)	164(83.7%)	88(83.0%)	
**N stage**				
N0-1	162(53.6%)	100(51.0%)	62(58.5%)	0.214
N2-3	140(46.4%)	96(49.0%)	44(41.5%)	
**Stage**				
III	161(53.3%)	102(52.0%)	59(55.7%)	0.547
IV	141(46.7%)	94(48.0%)	47(44.3%)	
**EBV DNA(copies/ml)**				
<2000	147(48.7%)	100(51.0%)	47(44.3%)	0.268
≥2000	155(51.3%)	96(49.0%)	59(55.7%)	
**VCA-IgA**				
<1:80	41(13.6%)	25(12.8%)	16(15.1%)	0.571
≥1:80	261(86.4%)	171(87.2%)	90(84.9%)	
**EA-IgA**				
<1:10	60(19.9%)	36(18.4%)	24(22.6%)	0.374
≥1:10	242(80.1%)	160(81.6%)	82(77.4%)	

Supplementary Table 3. Univariate and multivariate analysis of clinicopathological characteristics and overall survival in SYSUCC NPC cohort-2.

	Univariate	Multivariate	HR	*P* value
**Age**				
≤45 years	0.019			NS
>45 years				
**Sex**				
Male	0.005		2.096(1.163-3.777)	0.014
Female				
**WHO type**				
II	0.008		0.428(0.206-0.887)	0.022
III				
**Family history**				
No	0.492	n.d.		
Yes				
**T stage**				
T1-2	0.404	n.d.		
T3-4				
**N stage**				
N0-1	0.005			NS
N2-3				
**Stage**				
III	0.001		1.901(1.252-2.886)	0.003
IV				
**EBV DNA (copies/ml)**				
<2000	0.454	n.d.		
≥2000				
**VCA-IgA**				
<1:80	0.074	n.d.		
≥1:80				
**EA-IgA**				
<1:10	0.167	n.d.		
≥1:10				
**ACSL4 pT679**				
Low expression	<0.0001		0.371(0.219-0.631)	0.0002
High expression				

Supplementary Table 4. Primer sequences for qPT-PCR, siRNA and sgRNA assays.

Name	Forward	Reverse
Primer sequences for qRT-PCR		
Human GAPDH	5’-TGATGACATCAAGAAGGTGG-3’	5’-TTGTCATACCAGGAAATGAGC-3’
Human AIFM2	5’-GTGAGCGGGTGAGCAATCT-3’	5’-CTTGATGCCGGTGCAGAGAA-3’
Human PCK2	5’-AGTAGAGAGCAAGACGGTGAT-3’	5’-TGCTGAATGGAAGCACATACAT-3’
Human HERC6	5’-CCACTCCCTGGCATTATCAAAA-3’	5’-GCCAAACGAAGTCCCACAGA-3’
Human RNF215	5’-GGAGACTGGCATCCCTCAAGA-3’	5’-GGTGAAACTCGTGCTTACAGG-3’
Human RNF207	5’-GGCGGAGATCATGGGAGAC-3’	5’-CACTCGCTGATAGGCTTCCTC-3’
Human RNF39	5’-AGCCTGAGGTCTAATGTGCG-3’	5’-GTTGGGACTTCAAATCGTCTCC-3’
Human RNF208	5’-GGATGAGGTCATTGTGAATCAGT-3’	5’-GCTGGGTGACATTGTAGGAGTG-3’
Human TMEM129	5’-CTCTGTCAACACTGAGTTCCG-3’	5’-GACTCCGTCACAGTCAGGTG-3’
Human TRIM47	5’-TGTGTCCTATCAACTACCCCTTG-3’	5’-GGAGAAGTCTTCGGCCATGAC-3’
Human ACSL1	5’-CTTATGGGCTTCGGAGCTTTT-3’	5’-CAAGTAGTGCGGATCTTCGTG-3’
Human ACSL3	5’-ATGGAAAACCAACCTCATAGCAA-3’	5’-GCCATCCCAGTTATACCAGCAA-3’
Human ACSL4	5’-ACTGGCCGACCTAAGGGAG-3’	5’-GCCAAAGGCAAGTAGCCAATA-3’
Human FATP2	5’-GGCGCTCCTTATGGGTAACG-3’	5’-CTTGGCAGTATCTCTTCGACAG-3’
Human LPCAT3	5’-GGAGACCTACCTCATCCACCT-3’	5’-CGGCCCATTAGTCGAAGGA-3’
Human PLA2G6	5’-CATCACAGCCGTATCATCAGC-3’	5’-TCGGTGACATCCATCTGAGTG-3’
Human PLA2G2F	5’-AAGGCTGTCACCCCTATGTG-3’	5’-CCTCTCGGTACGTCTGGTTCA-3’
Human PLA2G4A	5’-TACCAGCACATTATAGTGGAGCA-3’	5’-GCTGTCAGGGGTTGTAGAGAT-3’
Human PCK1	5’-TTGAGAAAGCGTTCAATGCCA-3’	5’-CACGTAGGGTGAATCCGTCAG-3’
Human ALOX15	5’-GGGCAAGGAGACAGAACTCAA-3’	5’-CAGCGGTAACAAGGGAACCT-3’
Human PEBP1	5’-CCTGCAAGAAGTGGACGAG-3’	5’-ACCAAGGTGTAGAGCTTCCCT-3’
Primer sequences for siRNA		

Human PCK2-1	5’-GAUGAGGUUUGACAGUGAATT-3’	5’-UUCACUGUCAAACCUCAUCTT-3’
Human PCK2-2	5’-CCCAAGUACAAUAACUGCUTT-3’	5’-AGCAGUUAUUGUACUUGGGTT-3’
Human AIFM2-1	5’-GGGCAAGUUUAAUGAGGUUTT-3’	5’-AACCUCAUUAAACUUGCCCTT-3’
Human AIFM2-2	5’-GCUGCCUCUCAAUGAGUAUTT-3’	5’-AUACUCAUUGAGAGGCAGCTT-3’
Human HERC6-1	5’-GCACACACUGCGGUGCUUATT-3’	5’-UAAGCACCGCAGUGUGUGCTT-3’
Human HERC6-2	5’-GCUGAUCCAGAUGCUUAAATT-3’	5’-UUUAAGCAUCUGGAUCAGCTT-3’
Human TRIM47-1	5’-GGGACUAUUUCCUCAAGUUTT-3’	5’-AACUUGAGGAAAUAGUCCCTT-3’
Human TRIM47-2	5’-GCAGCUGUUUGGAACCAAATT-3’	5’-UUUGGUUCCAAACAGCUGCTT-3’
Human PKM-1	5’-GAUCAGUGGAGACGUUGAATT-3’	5’-UUCAACGUCUCCACUGAUCTT-3’
Human PKM-2	5’-GAUAACGCCUACAUGGAAATT-3’	5’-UUUCCAUGUAGGCGUUAUCTT-3’
Human PFKP-1	5’-GGGCCAAGGUGUACUUCAUTT-3’	5’-AUGAAGUACACCUUGGCCCTT-3’
Human PFKP-2	5’-GCUCCAUCGACAAUGAUUUTT-3’	5’-AAAUCAUUGUCGAUGGAGCTT-3’
Human PPM1G-1	5’-GCAAGCUACAGAAGGCUUUTT-3’	5’-AAAGCCUUCUGUAGCUUGCTT-3’
Human PPM1G-2	5’-CCUGAGGAACAGAUGAUUUTT-3’	5’-AAAUCAUCUGUUCCUCAGGTT-3’
Human SOX2-1	5’-CUGCAGUACAACUCCAUGATT-3’	5’-UCAUGGAGUUGUACUGCAGTT-3’
Human SOX2-2	5’-GCUCGCAGACCUACAUGAATT-3’	5’-UUCAUGUAGGUCUGCGAGCTT-3’
Human STAT3-1	5’-GCUGAACAACAUGUCAUUUTT-3’	5’-AAAUGACAUGUUGUUCAGCTT-3’
Human STAT3-2	5’-GCAACAGAUUGCCUGCAUUTT-3’	5’-AAUGCAGGCAAUCUGUUGCTT-3’
Human BMI1-1	5’-GCCACAACCAUAAUAGAAUTT-3’	5’-AUUCUAUUAUGGUUGUGGCTT-3’
Human BMI1-2	5’-CCUGGAGACCAGCAAGUAUTT-3’	5’-AUACUUGCUGGUCUCCAGGTT-3’
Human NANOG-1	5’-GACCAGAACUGUGUUCUCUTT-3’	5’-AGAGAACACAGUUCUGGUCTT-3’
Human NANOG-2	5’-GCAUCCGACUGUAAAGAAUTT-3’	5’-AUUCUUUACAGUCGGAUGCTT-3’
Human LPCAT3-1	5’-CCGGCAACUACGAUAUCAATT-3’	5’-UUGAUAUCGUAGUUGCCGGTT-3’
Human LPCAT3-2	5’-GGAGGGAAAGAUCAGAAUUTT-3’	5’-AAUUCUGAUCUUUCCCUCCTT-3’
Human PLA2G6-1	5’-CCAGCUGCUACCCUUCUAUTT-3’	5’-AUAGAAGGGUAGCAGCUGGTT-3’
Human PLA2G6-2	5’-CUGGCCAUGUCGAAAGACATT-3’	5’-UGUCUUUCGACAUGGCCAGTT-3’
Human PLA2G4A-1	5’-CCAGUAUUCCCACAAGUUUTT-3’	5’-AAACUUGUGGGAAUACUGGTT-3’

Human PLA2G4A-2	5’-CCUGGUAUAUGUCAACCUUTT-3’	5’-AAGGUUGACAUAUACCAGGTT-3’
Human ACSL1-1	5’-GGUGAUCGUUCCACUUUAUTT-3’	5’-AUAAAGUGGAACGAUCACCTT-3’
Human ACSL1-2	5’-CCUGAAGAUCUUGCAGUAATT-3’	5’-UUACUGCAAGAUCUUCAGGTT-3’
Human ACSL3-1	5’-CCCGAUGGAUGCUUAAAGATT-3’	5’-UCUUUAAGCAUCCAUCGGGTT-3’
Human ACSL3-2	5’-GGUUAUUCUUGGACAGUAUTT-3’	5’-AUACUGUCCAAGAAUAACCTT-3’
Human ACSL4-1	5’-GCAAUUUGAUAGCUGGAAUTT-3’	5’-AUUCCAGCUAUCAAAUUGCTT-3’
Human ACSL4-2	5’-GCAGAGAUAUCUUGCUUUATT-3’	5’-UAAAGCAAGAUAUCUCUGCTT-3’
Human SLC27A2-1	5’-GGUGUCGCCAGAACUACAATT-3’	5’-UUGUAGUUCUGGCGACACCTT-3’
Human SLC27A2-2	5’-UCACACAACUUACACCAUUTT-3’	5’-AAUGGUGUAAGUUGUGUGATT-3’
Primer sequences for sgRNA		
Human ACSL4-1	5’-CACCGAAGTGTGTGACAGAGCGATA-3’	5’-AAACTATCGCTCTGTCACACACTTC-3’
Human ACSL4-2	5’-CACCGCTAGCTGTAATAGACATCCC-3’	5’-AAACGGGATGTCTATTACAGCTAGC-3’
Human ACSL4-3	5’-CACCGTGCCAAGAAGAAAAACGCTA-3’	5’-AAACTAGCGTTTTTCTTCTTGGCAC-3’
Human PCK2-1	5’-CACCGCCACATCTGTGATGGAACTG-3’	5’-AAACCAGTTCCATCACAGATGTGGA-3’
Human PCK2-2	5’-CACCGCTGACCCTGCTGGAGCAGCA-3’	5’-AAACTGCTGCTCCAGCAGGGTCAGA-3’
Human PCK2-3	5’-CACCGACAAAATCTCGAATGCCAGT-3’	5’-AAACACTGGCATTCGAGATTTTGTA-3’
Human STAT3-1	5’-CACCGAGCTACAGCAGCTTGACACA-3’	5’-AAACTGTGTCAAGCTGCTGTAGCTC-3’
Human STAT3-2	5’-CACCGCTACAGTGACAGCTTCCCAA-3’	5’-AAACTTGGGAAGCTGTCACTGTAGC-3’
Human STAT3-3	5’-CACCGAATCTTGACTCTCAATCCAA-3’	5’-AAACTTGGATTGAGAGTCAAGATTC-3’
Human HERC6-1	5’-CACCGCCTGCAGTAGCTCAGCCCCG-3’	5’-AAACCGGGGCTGAGCTACTGCAGGC-3’
Human HERC6-2	5’-CACCGGCTGCTGCTGACCAACCACA-3’	5’-AAACTGTGGTTGGTCAGCAGCAGCC-3’
Human HERC6-3	5’-CACCGCCAGGGAGCTGCAGCGCCGG-3’	5’-AAACCCGGCGCTGCAGCTCCCTGGC-3’

Supplementary Table 5. List of antibodies used in this study.

Antibodies	Supplier	Catalogue	Application	Host species	Species activity	clone	Dilution
Antibodies used for Western blotting (WB), immunoprecipitation (IP) and immunofluorescence (IF)							
anti-mouse IgG, HRP-linked antibody	Cell Signaling Technology	7076S	WB	Horse	Mo	N.A	1:3000
anti-rabbit IgG, HRP-linked antibody	Cell Signaling Technology	7074S	WB	Goat	Rabbit	N.A	1:3000
anti-PCK2	Cell Signaling Technology	8565S	WB, IF, IP	Rabbit	Hu	N.A	1:1000 for WB, 1:100 for IF, 5μg for IP
anti-pStat3	Cell Signaling Technology	9145	WB	Rabbit	Hu	N.A	1:2000
anti-Stat3	Cell Signaling Technology	9139	WB	Mouse	Hu	N.A	1:2000
anti-SOX2	Cell Signaling Technology	3579	WB	Rabbit	Hu	N.A	1:1000
anti-SLC7A11	Cell Signaling Technology	12691	WB	Rabbit	Hu	N.A	1:1000
anti-Ubiquitin	Cell Signaling Technology	43124S	WB	Rabbit	Hu	N.A	1:1000
anti-tubulin	Cell Signaling Technology	3873	WB	Mouse	Hu	N.A	1:2000
anti-β-actin	Cell Signaling Technology	3700	WB	Mouse	Hu	N.A	1:2000
anti-Cytochrome c	Cell Signaling Technology	11940T	WB	Rabbit	Hu	N.A	1:2000
anti-Lamin B	Cell Signaling Technology	13435S	WB	Rabbit	Hu	N.A	1:2000
anti-Phospho-AMPKα (Thr172)	Cell Signaling Technology	2535T	WB	Rabbit	Hu	N.A	1:2000
anti-AMPKα	Cell Signaling Technology	5832T	WB	Rabbit	Hu	N.A	1:2000

anti-MYC	Cell Signaling Technology	2278S	WB	Rabbit	Hu	N.A	1:2000
anti-HA	Cell Signaling Technology	3724S	WB	Rabbit	Hu	N.A	1:2000
anti-Flag	Cell Signaling Technology	14793S	WB	Rabbit	Hu	N.A	1:2000
anti-PCK2	Abcam	ab70359	WB, IHC	Rabbit	Hu	N.A	1:2000 for WB, 1:100 for IHC
anti-ACSL4	Abcam	ab155282	WB, IF, IP	Rabbit	Hu	N.A	1:2000 for WB, 1:300 for IF, 5μg for IP
anti-LPCAT3	Abcam	ab239585	WB	Mouse	Hu	N.A	1:1000
anti-GPX4	Abcam	ab125066	WB	Rabbit	Hu	N.A	1:1000
anti-phospho Ser/Thr	Abcam	ab17464	WB	Rabbit	Hu	N.A	1:1000
anti-TIM22	Abcam	ab167423	WB	Rabbit	Hu	N.A	1:2000
anti-MnSOD	Abcam	ab68155	WB	Rabbit	Hu	EPR2560Y	1:2000
anti-Fibrin	Merck	MABS2155	IHC	Mouse	Hu	59D8	1:100
anti-iPLA2	Santa Cruz	sc-376563	WB	Mouse	Hu	D-4	1:1000
anti-FSP1	Santa Cruz	sc-377120	WB	Mouse	Hu	B-6	1:1000
anti-HERC6	NOVUS	NBP1-55025	WB	Rabbit	Hu	N.A	1:1000
anti-FATP2	NOVUS	NBP2-37738/6B3A9	WB	Mouse	Hu	6B3A9	1:1000
anti-ACSL4 (pT679)	Genscript Biotechnology	N.A	WB, IHC, IF	Rabbit	Hu	N.A	1:800 for WB, 1:100 for IHC, 1:100 for IF
Alexa Fluor 488 IgG	Invitrogen	A11008	IF	Goat	Rabbit	N.A	1:1000
Alexa Fluor 594 IgG	Invitrogen	A11012	IF	Goat	Rabbit	N.A	1:1000

Antibodies used for flow cytometric analysis							
**Antibodies**	**Supplier**	**Catalogue**	**Application**	**Host species**	**Species activity**	**clone**	**Dilution**
PE anti-human CD326	Biolegend	324205	Fc	Mouse	Hu	9C4	5ug per test
APC anti-human CD61	Biolegend	336411	Fc	Mouse	Hu	VI-PL2	5ug per test
BV421 anti-STAT3 Phospho (Tyr705)	Biolegend	651009	Fc	Mouse	Hu	13A3-1	5ug per test

Source Data-Supplementary Fig. 4

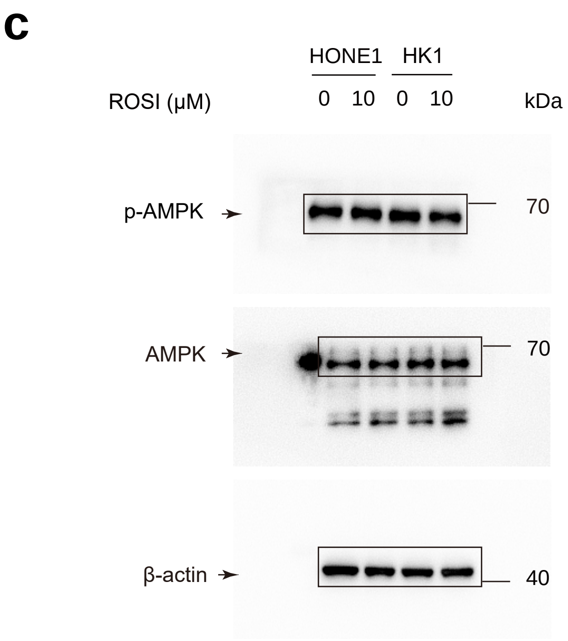

Source Data-Supplementary Fig. 7

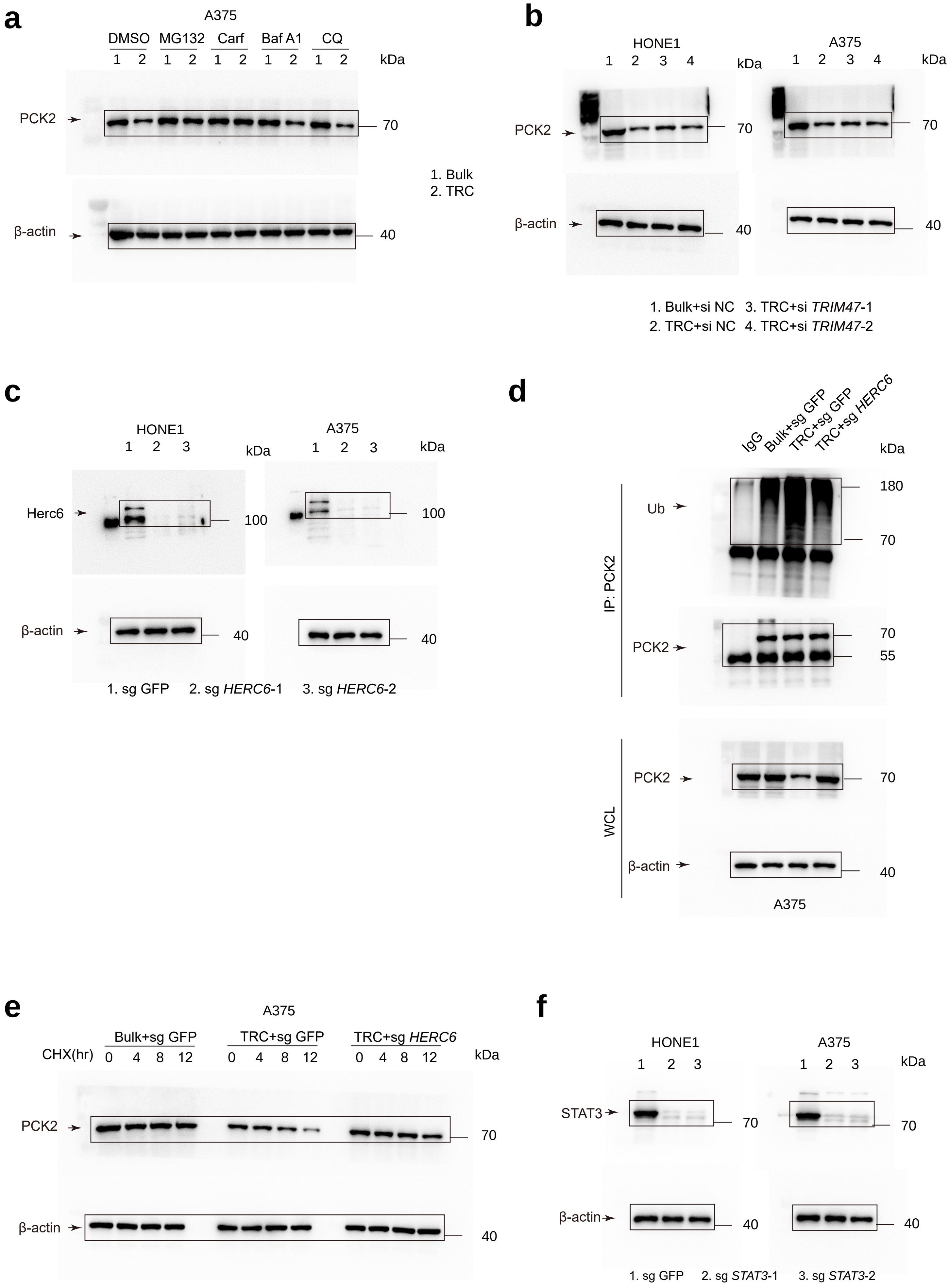

Supplement: Supplementary file 1 — Supplementary Figs. 1–9, Tables 1–5 and Source Data for Supplementary Figs. 4 and 7. [file 41589_2024_1612_MOESM1_ESM.pdf]
