# Supplementary material for: Tumor-repopulating cells evade ferroptosis via PCK2-dependent phospholipid remodeling
Source: Nat Chem Biol. 2024 May 8;20(10):1341–52. doi: 10.1038/s41589-024-01612-6 (PMC11427348; doi:10.1038/s41589-024-01612-6)
Supplement: Supplementary file 2 — Reporting Summary [file 41589_2024_1612_MOESM2_ESM.pdf]

nature portfolio

Corresponding author(s): Zhuo-wei Liu

Last updated by author(s): Mar 17, 2024

Reporting Summary

Nature Portfolio wishes to improve the reproducibility of the work that we publish. This form provides structure for consistency and transparency in reporting. For further information on Nature Portfolio policies, see our Editorial Policies and the Editorial Policy Checklist.

Statistics

For all statistical analyses, confirm that the following items are present in the figure legend, table legend, main text, or Methods section.

n/a	Confirmed	
☐	☒	The exact sample size (*n*) for each experimental group/condition, given as a discrete number and unit of measurement
☐	☒	A statement on whether measurements were taken from distinct samples or whether the same sample was measured repeatedly
☐	☒	The statistical test(s) used AND whether they are one- or two-sided<br>*Only common tests should be described solely by name; describe more complex techniques in the Methods section.*
☐	☒	A description of all covariates tested
☐	☒	A description of any assumptions or corrections, such as tests of normality and adjustment for multiple comparisons
☐	☒	A full description of the statistical parameters including central tendency (e.g. means) or other basic estimates (e.g. regression coefficient) AND variation (e.g. standard deviation) or associated estimates of uncertainty (e.g. confidence intervals)
☐	☒	For null hypothesis testing, the test statistic (e.g. *F*, *t*, *r*) with confidence intervals, effect sizes, degrees of freedom and *P* value noted<br>*Give P values as exact values whenever suitable.*
☒	☐	For Bayesian analysis, information on the choice of priors and Markov chain Monte Carlo settings
☒	☐	For hierarchical and complex designs, identification of the appropriate level for tests and full reporting of outcomes
☒	☐	Estimates of effect sizes (e.g. Cohen's *d*, Pearson's *r*), indicating how they were calculated

Our web collection on statistics for biologists contains articles on many of the points above.

Software and code

Policy information about availability of computer code

Data collection	Flow Cytometry data was collected with cytoFLEX LX and CytExpert 2.4; Real-time PCR data was collected with Bio-Rad CFX96; Immunofluorescence images were obtained with confocal laser-scanning microscope (LSM880, Zeiss); LC/MS data was collected with Q-Exactive mass spectrometer(Thermo) and Xcalibur 4.2 Quan Browser(Thermo);The absorbance data was measured with Bio-Tek EPOCH2 Microplate Reader; Microscale thermophoresis data was measured with Monolith NT.115 instrument (Nano Temper Technologies).
Data analysis	GraphPad Prism 8 and SPSS Statistics version 25 were used for data analysis; FACS data were analyzed with FlowJo 10; RNA-seq data were analyzed with R version 4.2.3; raw LC-MS data was analyzed with MZmine 2.5.3 and MS-DIAL; Principle component analysis (PCA) was performed using SIMCA 13.0 software. IHC images were obtained with AxioVision Rel.4.6 computerized image analysis system (Carl Zeiss).

For manuscripts utilizing custom algorithms or software that are central to the research but not yet described in published literature, software must be made available to editors and reviewers. We strongly encourage code deposition in a community repository (e.g. GitHub). See the Nature Portfolio guidelines for submitting code & software for further information.

Data

Policy information about availability of data

All manuscripts must include a data availability statement. This statement should provide the following information, where applicable:

- Accession codes, unique identifiers, or web links for publicly available datasets
- A description of any restrictions on data availability
- For clinical datasets or third party data, please ensure that the statement adheres to our policy

All data supporting the findings of this study are available within the Article, supplementary information or Source Data file. All original data for this study can be obtained from the corresponding author. Tumor metastasis data were obtained from Gene Expression Omnibus (https://www.ncbi.nlm.nih.gov/geo/, accession numbers: GSE87211, GSE45114, GSE15605 and GSE22541).This paper does not report original code.

Human research participants

Policy information about studies involving human research participants and Sex and Gender in Research.

Reporting on sex and gender	The gender information of patients with nasopharyngeal carcinoma were retrospectively collected through the medical record.
Population characteristics	All samples were pathologically confirmed as nasopharyngeal carcinoma. The clinical characteristics of patients are listed in Supplementary Table 1-3.
Recruitment	All samples were obtained from the Sun Yat-sen University Cancer Center, Sun Yat-sen University, Guangzhou. All patients were pathologically diagnosed with nasopharyngeal carcinoma and collected with informed consent between January 2006 and December 2009 or January 2020 and December 2022.
Ethics oversight	The Institutional Ethical Review Boards of Sun Yat-sen University Cancer Center approved this study (B2022-569).

Note that full information on the approval of the study protocol must also be provided in the manuscript.

Field-specific reporting

Please select the one below that is the best fit for your research. If you are not sure, read the appropriate sections before making your selection.

☒ Life sciences ☐ Behavioural & social sciences ☐ Ecological, evolutionary & environmental sciences

For a reference copy of the document with all sections, see nature.com/documents/nr-reporting-summary-flat.pdf

Life sciences study design

All studies must disclose on these points even when the disclosure is negative.

Sample size	No statistical methods were used to predetermine sample size. Same size were chosen based on previous experience for each experimentaiming to provide sufficient sample number for common statistical test including one way ANOVA or two way ANOVA used in this study.Experiments were performed with three samples per condition unless stated otherwise. The exact number of sample for each experiment wasnoted in the associated figure legend.
Data exclusions	No data was excluded for all in vitro experiments.
Replication	As reported in the figure legends, experiments were performed at least three times with similar results, the findings were reliably reproduced.
Randomization	For all in vivo experiments, animals were randomly assigned into a treatment group after tumor inoculation. The starting tumor burden in the treatment and control groups was similar before treatment. For all in vitro experiments, samples were randomly assigned to different treatment conditions.
Blinding	The investigators were not blinded to sample allocation during experiment and outcome assessment except animal experiment, because results used were obtained using objective quantitative methods. In animal experiments, the person performing data collection were blinded to group allocation.

Reporting for specific materials, systems and methods

We require information from authors about some types of materials, experimental systems and methods used in many studies. Here, indicate whether each material, system or method listed is relevant to your study. If you are not sure if a list item applies to your research, read the appropriate section before selecting a response.

Materials & experimental systems

n/a	Involved in the study
☐	☒ Antibodies
☐	☒ Eukaryotic cell lines
☒	☐ Palaeontology and archaeology
☐	☒ Animals and other organisms
☒	☐ Clinical data
☒	☐ Dual use research of concern

Methods

n/a	Involved in the study
☒	☐ ChIP-seq
☐	☒ Flow cytometry
☒	☐ MRI-based neuroimaging

Antibodies

Antibodies used

Antibodies used for Western blotting (WB), immunoprecipitation (IP) and immunofluorescence (IF):
anti-mouse IgG, HRP-linked Antibody, Cell Signaling Technology, 7076S, 1:3000 for WB
anti-rabbit IgG, HRP-linked Antibody, Cell Signaling Technology, 7074S, 1:3000 for WB
anti-PCK2, Cell Signaling Technology, 8565S, 1:1000 for WB, 1:100 for IF, 5μg for IP
anti-pStat3, Cell Signaling Technology, 9145, 1:2000 for WB
anti-Stat3, Cell Signaling Technology, 9139, 1:2000 for WB
anti-SOX2, Cell Signaling Technology, 3579, 1:1000 for WB
anti-SLC7A11, Cell Signaling Technology, 12691, 1:1000 for WB
anti-Ubiquitin, Cell Signaling Technology, 43124S, 1:1000 for WB
anti-tubulin, Cell Signaling Technology, 3873, 1:2000 for WB
anti-β-actin, Cell Signaling Technology, 3700, 1:2000 for WB
anti-Cytochrome c, Cell Signaling Technology, 11940T, 1:2000 for WB
anti-Lamin B, Cell Signaling Technology, 13435S, 1:2000 for WB
anti-Phospho-AMPKα (Thr172), Cell Signaling Technology, 2535T, 1:2000 for WB
anti-AMPKα, Cell Signaling Technology, 5832T, 1:2000 for WB
anti-MYC, Cell Signaling Technology, 2278S, 1:2000 for WB
anti-HA, Cell Signaling Technology, 3724S, 1:2000 for WB
anti-Flag, Cell Signaling Technology, 14793S, 1:2000 for WB
anti-PCK2, Abcam, ab70359, 1:2000 for WB, 1:100 for IHC
anti-ACSL4, Abcam, ab155282, 1:2000 for WB, 1:300 for IF, 5μg for IP
anti-LPCAT3, Abcam, ab239585, 1:1000 for WB
anti-GPX4, Abcam, ab125066, 1:1000 for WB
anti-phospho Ser/Thr, Abcam, ab17464, 1:1000 for WB
anti-TIM22, Abcam, ab167423, 1:2000 for WB
anti-MnSOD, Abcam, ab68155, 1:2000 for WB, clone EPR2560Y
anti-Fibrin, Merck, MABS2155, 1:100 for IHC, clone 59D8
anti-iPLA2, Santa Cruz, sc-376563, 1:1000 for WB, clone D-4
anti-FSP1, Santa Cruz, sc-377120, 1:1000 for WB, clone B-6
anti-HERC6, NOVUS, NBP1-55025, 1:1000 for WB
anti-FATP2, NOVUS, NBP2-37738/6B3A9, 1:1000 for WB, clone 6B3A9
anti-ACSL4 (pT679), Genscript Biotechnology, 1:800 for WB, 1:100 for IHC, 1:100 for IF
Alexa Fluor 488 IgG, Invitrogen, A11008, 1:1000 for IF
Alexa Fluor 594 IgG, Invitrogen, A11012, 1:1000 for IF

Antibodies used for flow cytometric analysis:
PE anti-human CD326, Biolegend, 324205, clone 9C4, 5ug per test
APC anti-human CD61, Biolegend, 336411, clone VI-PL2,5ug per test
BV421 anti-STAT3 Phospho (Tyr705), Biolegend, 651009, clone 13A3-1,5ug per test

Validation

Antibodies used for Western blotting (WB), immunoprecipitation (IP) and immunofluorescence (IF):
anti-mouse IgG, HRP-linked Antibody, Cell Signaling Technology, 7076S, 1:3000, https://www.cellsignal.com/products/secondary-antibodies/anti-mouse-igg-hrp-linked-antibody/7076
anti-rabbit IgG, HRP-linked Antibody, Cell Signaling Technology, 7074S, 1:3000, https://www.cellsignal.com/products/secondary-antibodies/anti-rabbit-igg-hrp-linked-antibody/7074
anti-PCK2, Cell Signaling Technology, 8565S, 1:1000 for WB, 1:100 for IF, 5μg for IP, https://www.cellsignal.com/products/primary-antibodies/pck2-d3e11-rabbit-mab/8565
anti-pStat3, Cell Signaling Technology, 9145, 1:2000, https://www.cellsignal.com/products/primary-antibodies/phospho-stat3-tyr705-d3a7-xp-rabbit-mab/9145
anti-Stat3, Cell Signaling Technology, 9139, 1:2000, https://www.cellsignal.com/products/primary-antibodies/stat3-124h6-mouse-mab/9139
anti-SOX2, Cell Signaling Technology, 3579, 1:1000, https://www.cellsignal.com/products/primary-antibodies/sox2-d6d9-xp-rabbit-mab/3579
anti-SLC7A11, Cell Signaling Technology, 12691, 1:1000, https://www.cellsignal.com/products/primary-antibodies/xct-slc7a11-d2m7a-rabbit-mab/12691
anti-Ubiquitin, Cell Signaling Technology, 43124S, 1:1000, https://www.cellsignal.com/products/primary-antibodies/ubiquitin-e4i2j-

rabbit-mab/43124
anti-tubulin, Cell Signaling Technology, 3873, 1:2000, https://www.cellsignal.com/products/primary-antibodies/a-tubulin-dm1a-mouse-mab/3873
anti-β-actin, Cell Signaling Technology, 3700, 1:2000, https://www.cellsignal.com/products/primary-antibodies/b-actin-8h10d10-mouse-mab/3700
anti-Cytochrome c, Cell Signaling Technology, 11940T, 1:2000, https://www.cellsignal.com/products/primary-antibodies/cytochrome-c-d18c7-rabbit-mab/11940
anti-Lamin B, Cell Signaling Technology, 13435S, 1:2000, https://www.cellsignal.com/products/primary-antibodies/phospho-ampka-thr172-40h9-rabbit-mab/2535
anti-Phospho-AMPKα (Thr172), Cell Signaling Technology, 2535T, 1:2000, https://www.cellsignal.com/products/primary-antibodies/phospho-ampka-thr172-40h9-rabbit-mab/2535
anti-AMPKα, Cell Signaling Technology, 5832T, 1:2000, https://www.cellsignal.com/products/primary-antibodies/ampka-d63g4-rabbit-mab/5832
anti-MYC, Cell Signaling Technology, 2278S, 1:2000, https://www.cellsignal.com/products/primary-antibodies/myc-tag-71d10-rabbit-mab/2278
anti-HA, Cell Signaling Technology, 3724S, 1:2000, https://www.cellsignal.com/products/primary-antibodies/ha-tag-c29f4-rabbit-mab/3724
anti-Flag, Cell Signaling Technology, 14793S, 1:2000, https://www.cellsignal.com/products/primary-antibodies/dykddddk-tag-d6w5b-rabbit-mab-binds-to-same-epitope-as-sigma-aldrich-anti-flag-m2-antibody/14793
anti-PCK2, Abcam, ab70359, 1:2000 for WB, 1:100 for IHC, https://www.abcam.com/products/primary-antibodies/pck2-antibody-ab70359.html
anti-ACSL4, Abcam, ab155282, 1:2000 for WB, 1:300 for IF, 5ug for IP, https://www.abcam.com/products/primary-antibodies/facl4-antibody-epr8640-ab155282.html
anti-LPCAT3, Abcam, ab239585, 1:1000, https://www.abcam.com/products/primary-antibodies/lpcat3-antibody-6-2-ab239585.html
anti-GPX4, Abcam, ab125066, 1:1000, https://www.abcam.com/products/primary-antibodies/glutathione-peroxidase-4-antibody-epncir144-ab125066.html
anti-phospho Ser/Thr, Abcam, ab17464, 1:1000, https://www.abcam.com/products/primary-antibodies/phospho-serthr-phe-antibody-ab17464.html
anti-TIM22, Abcam, ab167423, 1:2000, https://www.abcam.com/products/primary-antibodies/tim22-antibody-epr9973-ab167423.html
anti-MnSOD, Abcam, ab68155, 1:2000, clone EPR2560Y, https://www.abcam.com/products/primary-antibodies/sod2mnsod-antibody-epr2560y-ab68155.html
anti-Fibrin, Merck, MABS2155, 1:100 for IHC, clone 59D8, https://www.emdmillipore.com/US/en/product/Anti-Fibrin-Antibody-clone-59D8,MM_NF-MABS2155-100UG?ReferrerURL=https%3A%2F%2Fwww.google.com%2F
anti-iPLA2, Santa Cruz, sc-376563, 1:1000, D-4, https://www.scbt.com/p/group-vi-ipla2-antibody-d-4
anti-FSP1, Santa Cruz, sc-377120, 1:1000, B-6, https://www.scbt.com/p/amid-antibody-b-6
anti-HERC6, NOVUS, NBP1-55025, 1:1000, https://www.novusbio.com/products/herc6-antibody_nbp1-55025
anti-FATP2, NOVUS, NBP2-37738/6B3A9, 1:1000, 6B3A9, https://www.novusbio.com/products/fatp2-slc27a2-antibody-6b3a9_nbp2-37738
anti-ACSL4 (pT679), Genscript Biotechnology, 1:800 for WB, 1:100 for IHC, 1:100 for IF

Antibodies used for flow cytometric analysis:
PE anti-human CD326, Biolegend, 324205, clone 9C4, https://www.biolegend.com/en-gb/products/pe-anti-human-cd326-epcam-antibody-3757?GroupID=BLG5134
APC anti-human CD61, Biolegend, 336411, clone VI-PL2, https://www.biolegend.com/nl-nl/clone-search/apc-anti-human-cd61-antibody-7060?GroupID=BLG10331
BV421 anti-STAT3 Phospho (Tyr705), Biolegend, 651009, clone 13A3-1, https://www.biolegend.com/en-ie/products/brilliant-violet-421-anti-stat3-phospho-tyr705-antibody-13030?GroupID=BLG9459

Eukaryotic cell lines

Policy information about cell lines and Sex and Gender in Research

Cell line source(s)	Human tumor cell lines (A375 and HCT116) and HEK293T cells were obtained from China Center for Type Culture Collection. The human NPC cell lines HONE1 and HK1 were provided by Professor Mu-Sheng Zeng at Sun Yat-sen University Cancer Center.
Authentication	STR fingerprint analysis
Mycoplasma contamination	All cell lines in our laboratory are routinely tested for mycoplasma contamination and cells used in this study are negative for mycoplasma.
Commonly misidentified lines (See ICLAC register)	No cell line used in the paper is listed in ICLAC database.

Animals and other research organisms

Policy information about studies involving animals; ARRIVE guidelines recommended for reporting animal research, and Sex and Gender in Research

Laboratory animals	For the in vivo experiments, 6- to 10- week-old female BALB/c nude mice or C57BL/6J were purchased from Charles River Laboratories (Zhejiang, China). These animals were specific pathogen-free and maintained in 5 animals per group on a 12/12-h light/

	dark cycle at 20-26 ℃ with around 40-70% humidity, and give ad libitum access to standard food and water.
Wild animals	The study did not involve wild animals.
Reporting on sex	All animals used in this study were female, and no sex-based analysis was performed.
Field-collected samples	The study did not involve samples collected from field.
Ethics oversight	All animal experiments were approved by the Animal Care and Use Committee of Sun Yat-Sen University.

Note that full information on the approval of the study protocol must also be provided in the manuscript.

Flow Cytometry

Plots

Confirm that:

☒ The axis labels state the marker and fluorochrome used (e.g. CD4-FITC).

☒ The axis scales are clearly visible. Include numbers along axes only for bottom left plot of group (a 'group' is an analysis of identical markers).

☒ All plots are contour plots with outliers or pseudocolor plots.

☒ A numerical value for number of cells or percentage (with statistics) is provided.

Methodology

Sample preparation	For sorting EpCAM+ CD61+ cells, HONE1 tumors from BALB/c nude mice were harvest and digested into single-cell suspension. Cells were resuspended in PBS containing 1% FBS and stained with fluorescent-conjugated antibodies against CD326 and CD61 for 30min at 4°C and analyzed by flow cytometry.<br><br>For cell death analysis, the cells were treated with the indicated compounds and then collected, stained with 7-AAD (Biolegend, 420404) at 25 °C for 15 min and analyzed by flow cytometry.
Instrument	cytoFLEX LX
Software	CytExpert and FlowJo version 10
Cell population abundance	When cells were sorted or enriched, the purity was confirmed by flow cytometry and in each case was above 90% purity.
Gating strategy	The cells were gated on FSC-A/SSC-A basis on the location contain all cells. EpCAM+ CD61+ population were gated.

☒ Tick this box to confirm that a figure exemplifying the gating strategy is provided in the Supplementary Information.
