# Supplementary figures and images for: Tumor-repopulating cells evade ferroptosis via PCK2-dependent phospholipid remodeling
Source: Nat Chem Biol. 2024 May 8;20(10):1341–52. doi: 10.1038/s41589-024-01612-6 (PMC11427348; doi:10.1038/s41589-024-01612-6)

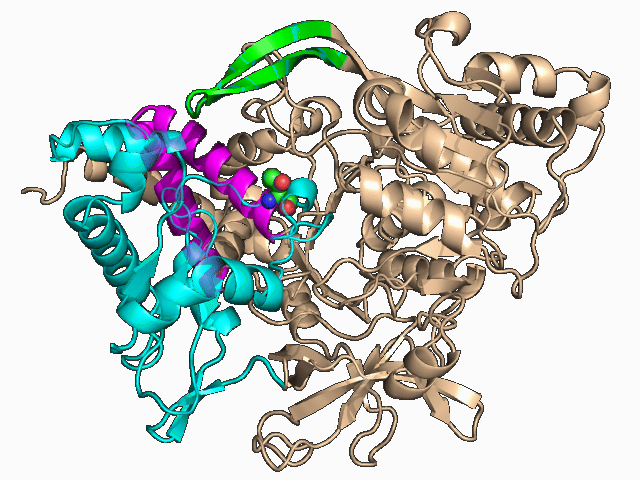

Supplement: Supplementary file 3 — PC1 motion trajectory of ACSL4 WT model. [file 41589_2024_1612_MOESM3_ESM.gif]

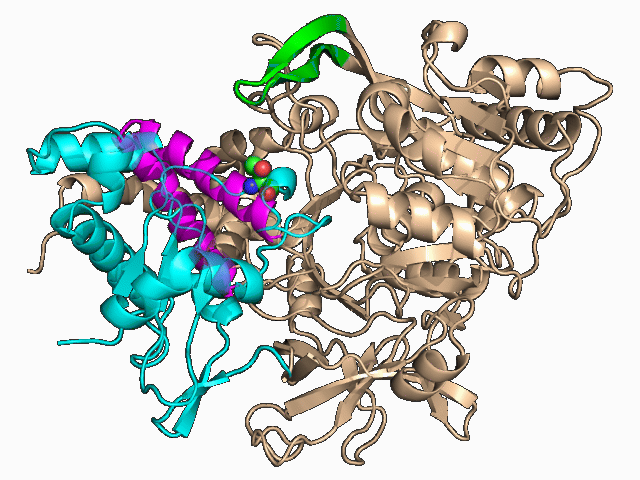

Supplement: Supplementary file 4 — PC2 motion trajectory of ACSL4 WT model. [file 41589_2024_1612_MOESM4_ESM.gif]

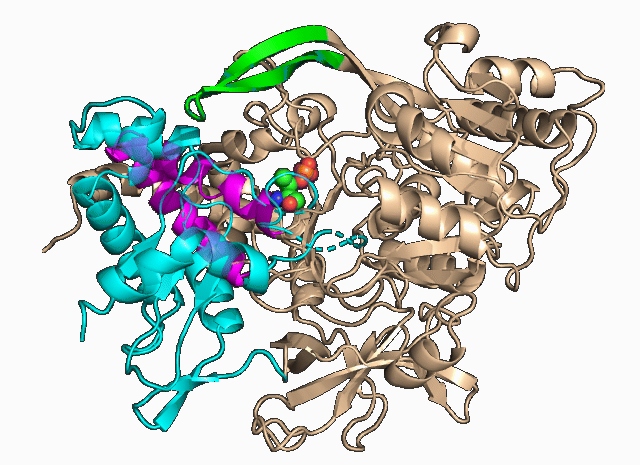

Supplement: Supplementary file 5 — PC1 motion trajectory of ACSL4 phosphorylation model. [file 41589_2024_1612_MOESM5_ESM.gif]

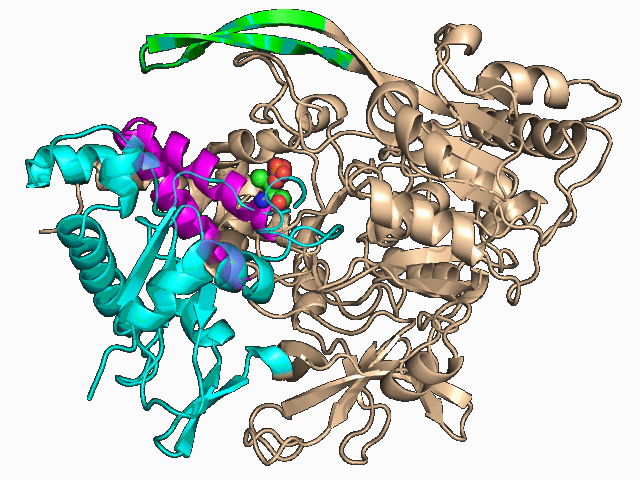

Supplement: Supplementary file 6 — PC2 motion trajectory of ACSL4 phosphorylation model. [file 41589_2024_1612_MOESM6_ESM.gif]

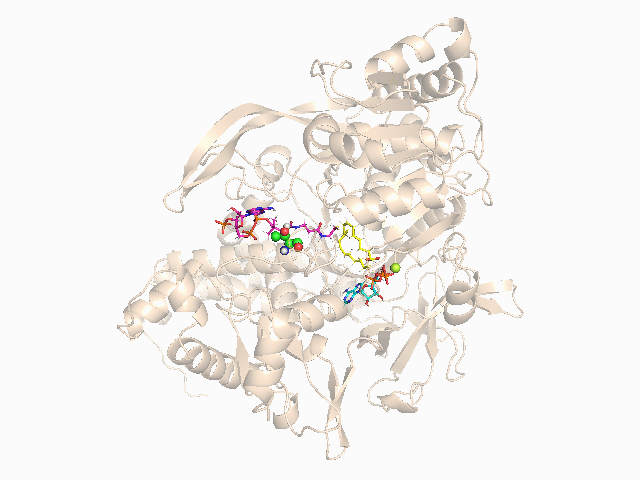

Supplement: Supplementary file 7 — Representative conformations of CoA in ACSL4 WT model. [file 41589_2024_1612_MOESM7_ESM.gif]

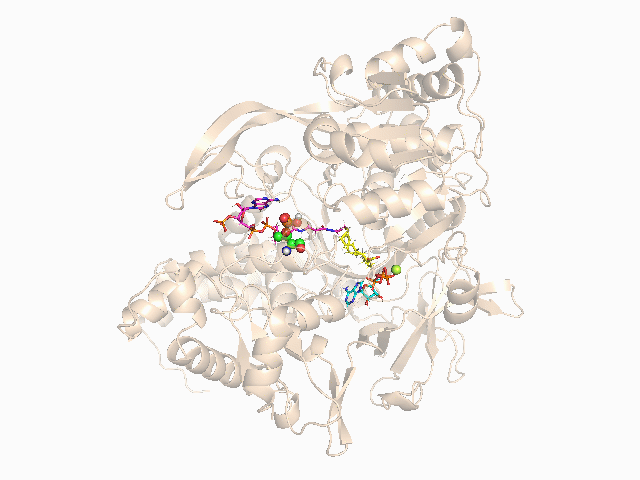

Supplement: Supplementary file 9 — Representative conformations of CoA in ACSL4 phosphorylation model. [file 41589_2024_1612_MOESM9_ESM.gif]

Fig. 3

a

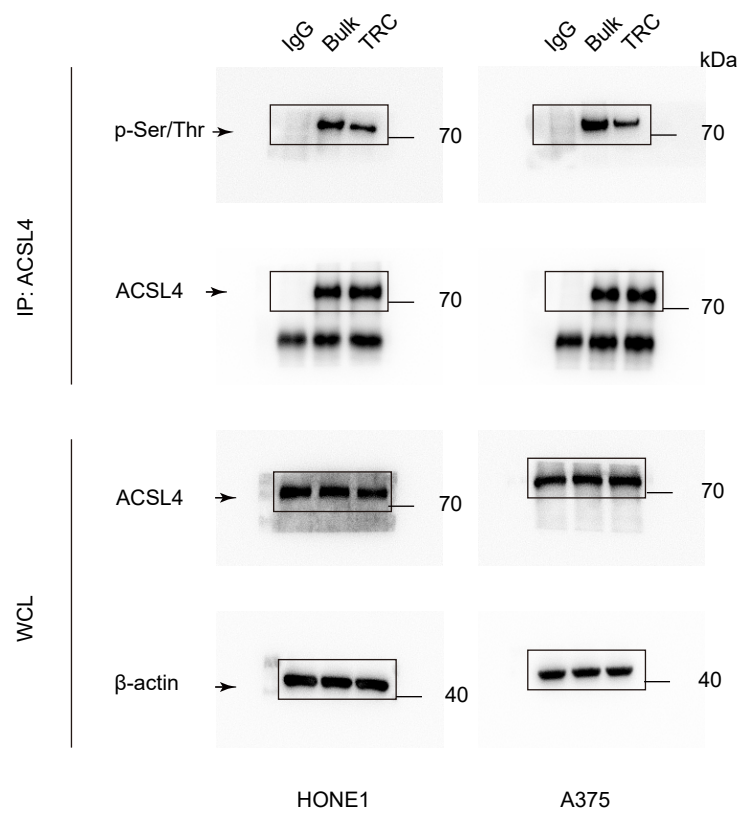

Supplement: Supplementary file 13 — Unprocessed western blots. [file 41589_2024_1612_MOESM13_ESM.zip › Li_unmodified_Gels_Fig/Li_unmodified_Gels_Fig3.pdf]

**Fig. 4**

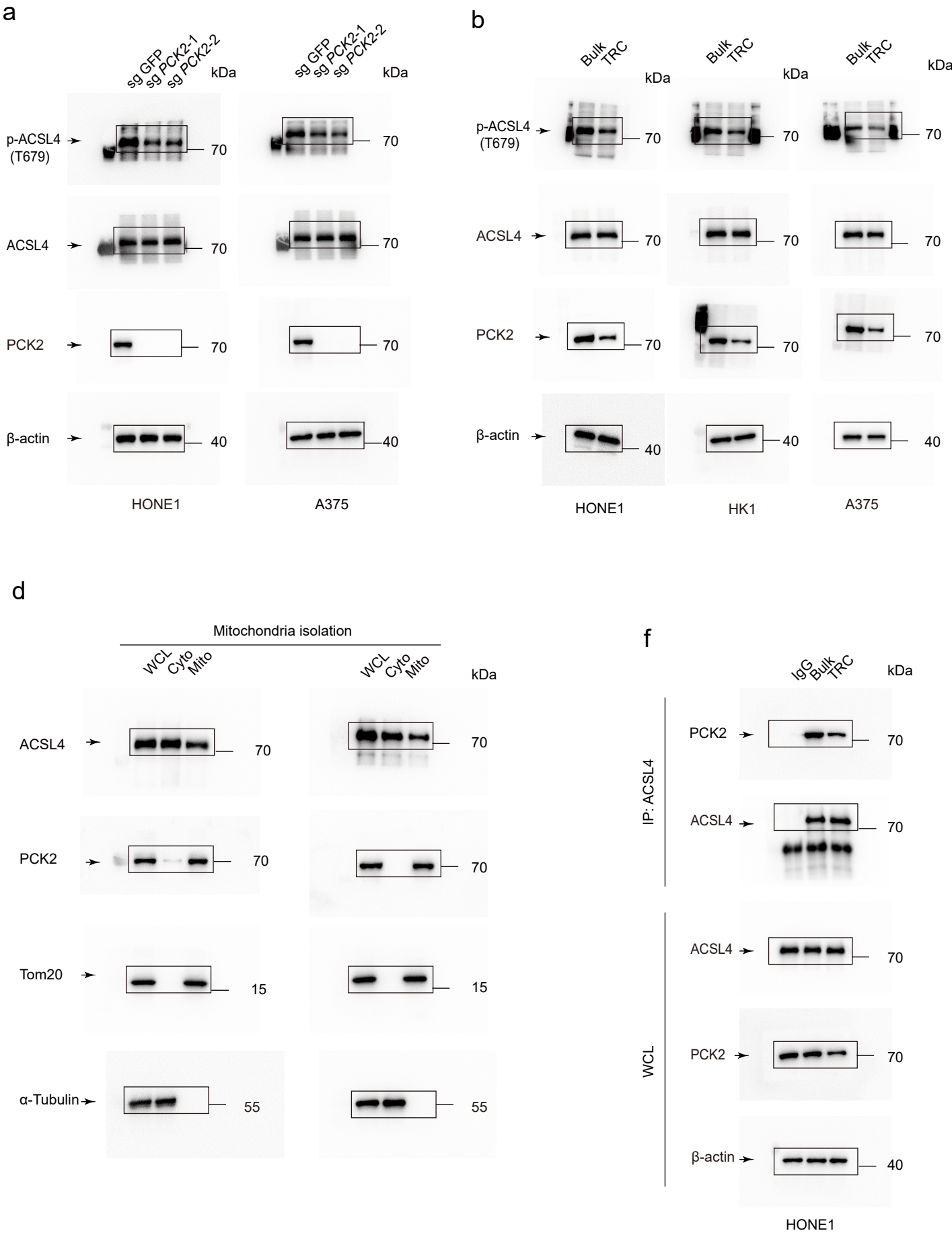

Fig. 4

j

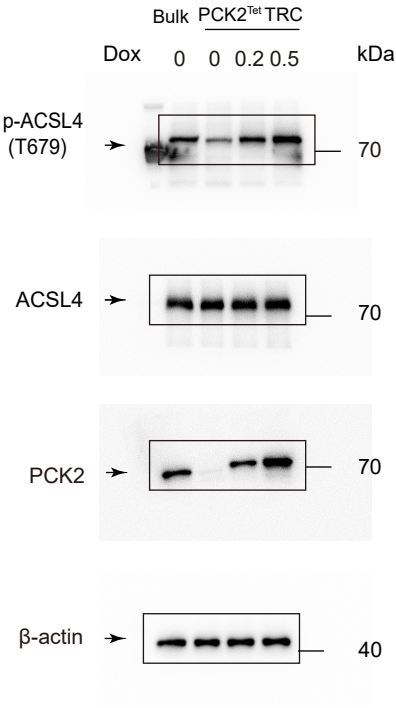

Supplement: Supplementary file 13 — Unprocessed western blots. [file 41589_2024_1612_MOESM13_ESM.zip › Li_unmodified_Gels_Fig/Li_unmodified_Gels_Fig4.pdf]

**Fig. 5**

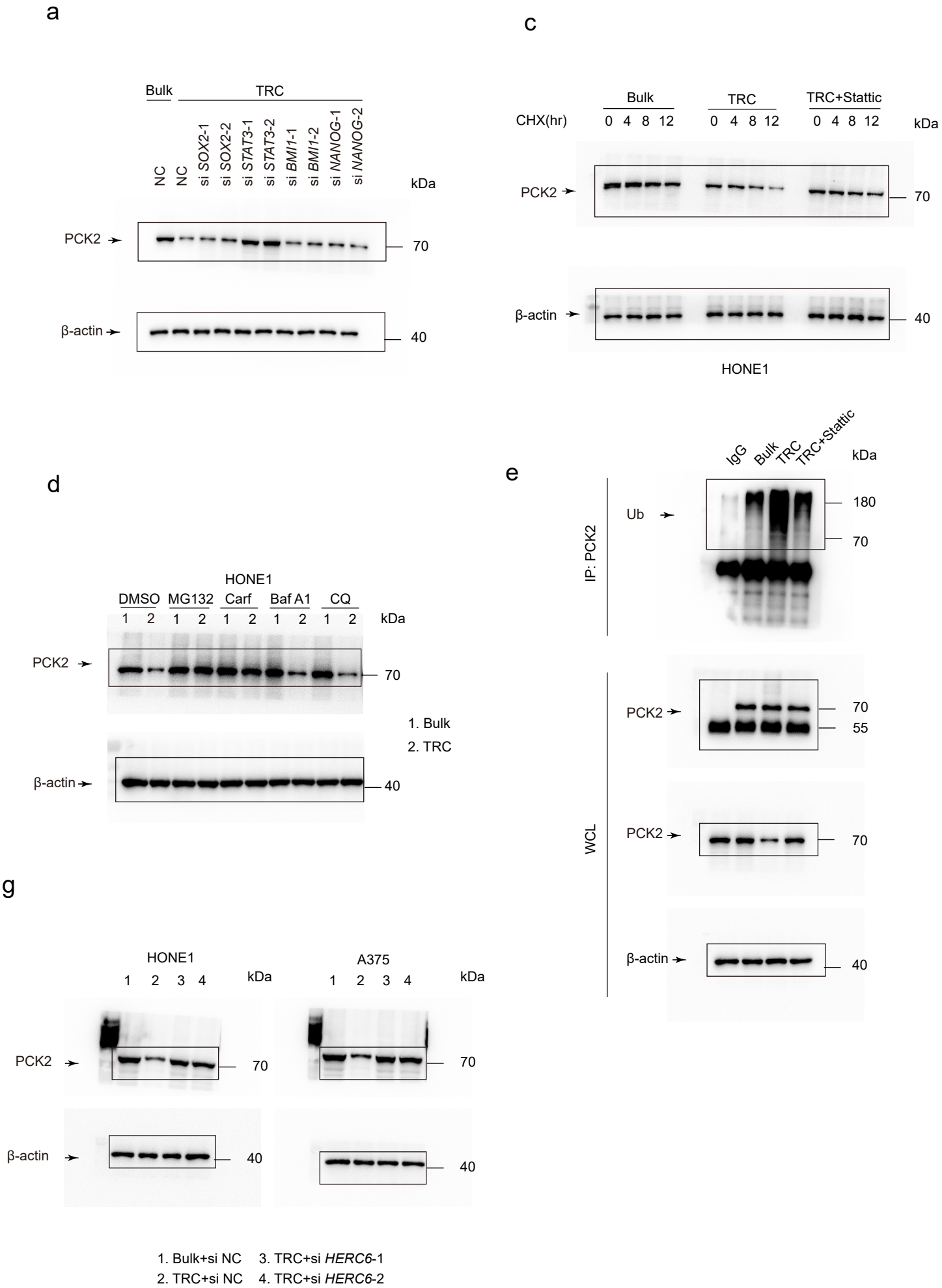

Supplement: Supplementary file 13 — Unprocessed western blots. [file 41589_2024_1612_MOESM13_ESM.zip › Li_unmodified_Gels_Fig/Li_unmodified_Gels_Fig5.pdf]

Extended Data Fig. 1

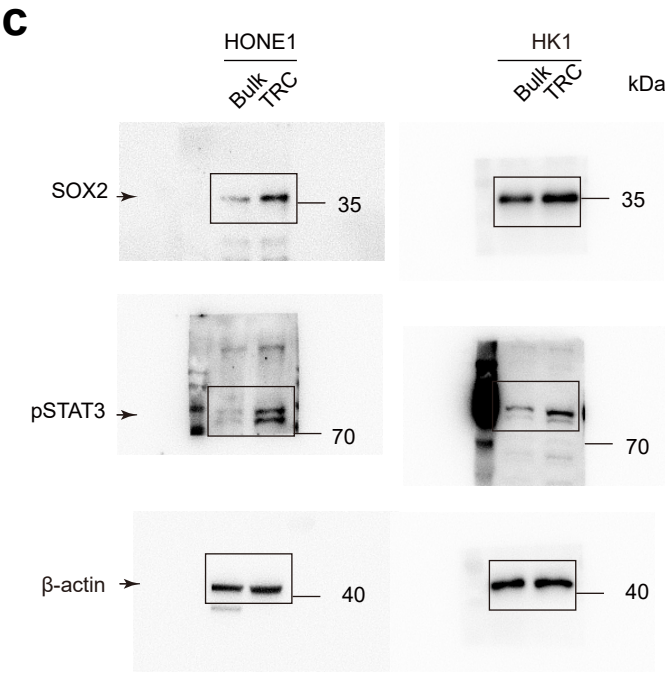

Supplement: Supplementary file 15 — Unprocessed western blots. [file 41589_2024_1612_MOESM15_ESM.zip › Li_unmodified_Gels_ED_Fig/Li_unmodified_Gels_ED_Fig1.pdf]

Extended Data Fig. 2

**a**

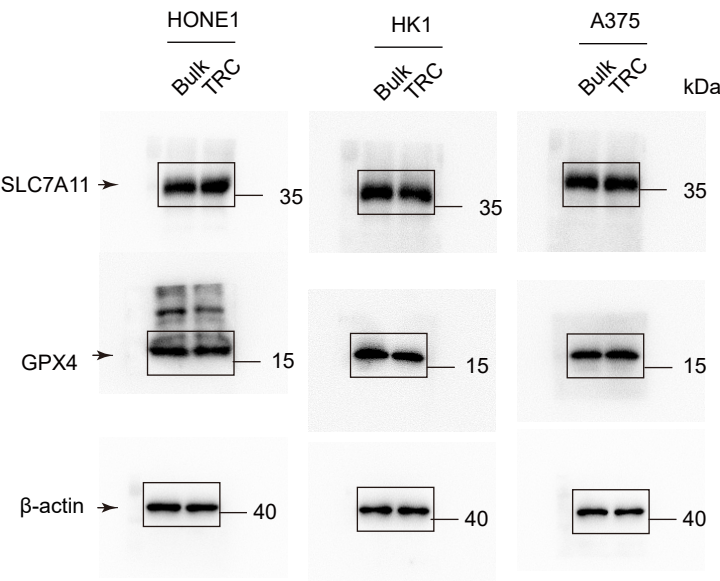

**e**

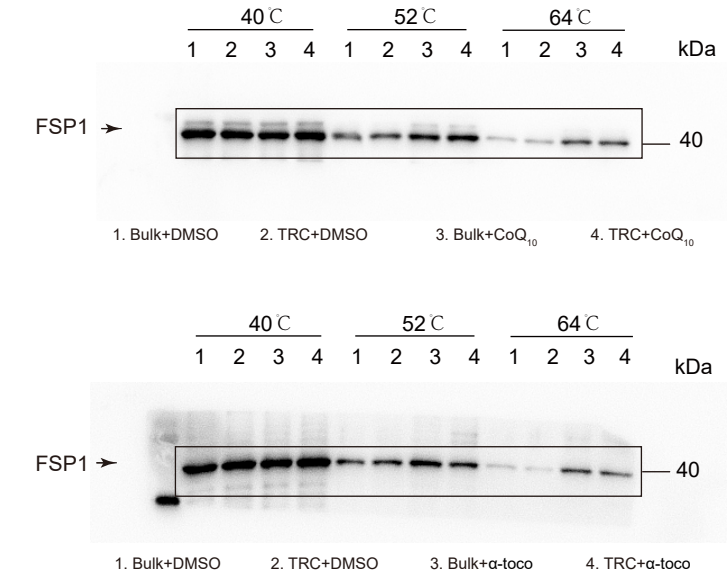

**f**

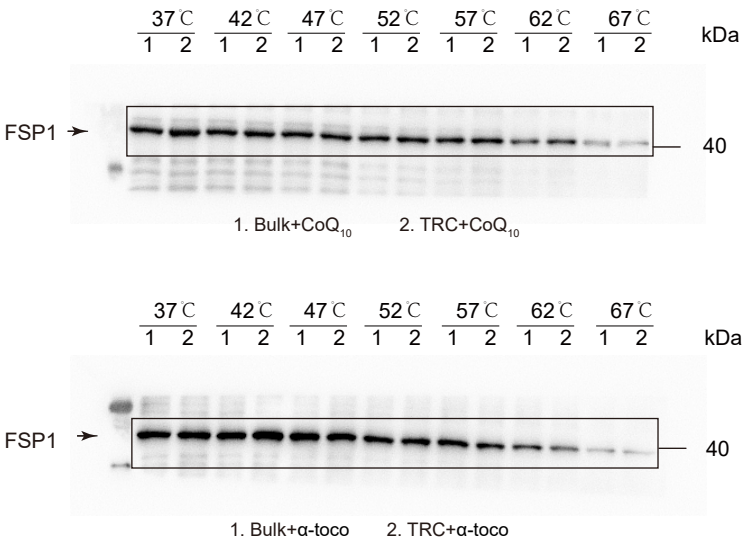

Supplement: Supplementary file 15 — Unprocessed western blots. [file 41589_2024_1612_MOESM15_ESM.zip › Li_unmodified_Gels_ED_Fig/Li_unmodified_Gels_ED_Fig2.pdf]

Extended Data Fig. 3

a

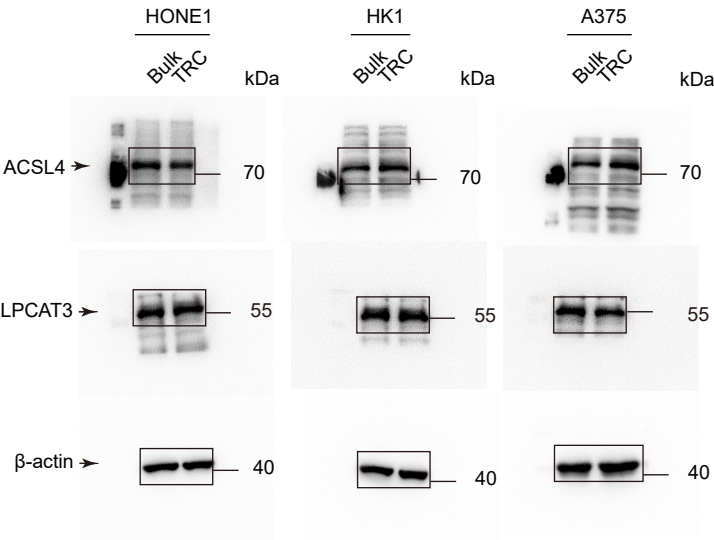

d

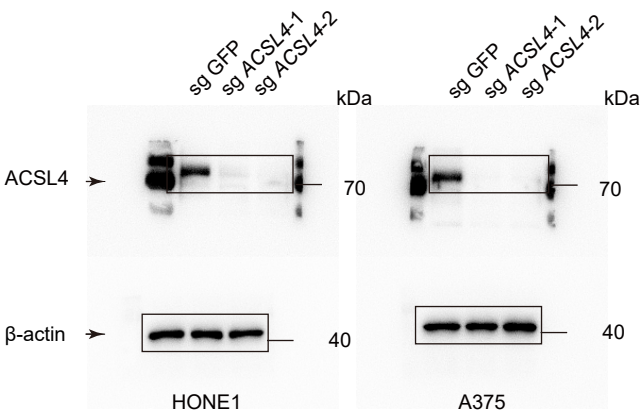

h

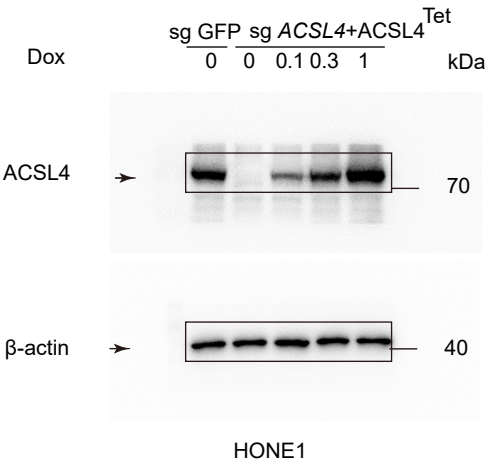

j

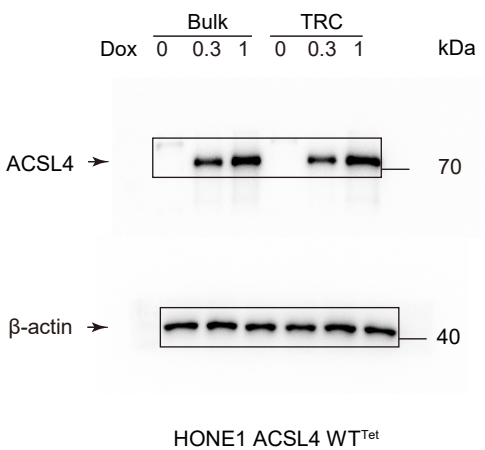

m

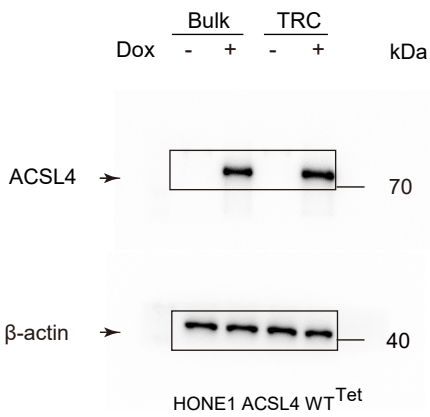

Supplement: Supplementary file 15 — Unprocessed western blots. [file 41589_2024_1612_MOESM15_ESM.zip › Li_unmodified_Gels_ED_Fig/Li_unmodified_Gels_ED_Fig3.pdf]

Extended Data Fig. 4

**b**

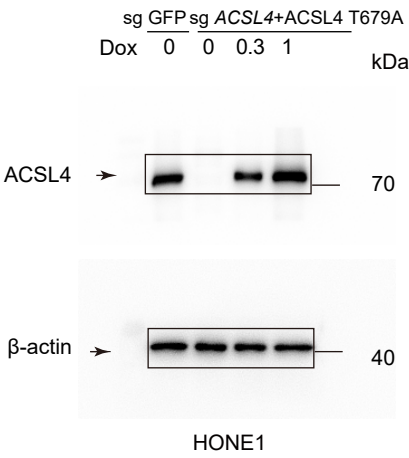

Supplement: Supplementary file 15 — Unprocessed western blots. [file 41589_2024_1612_MOESM15_ESM.zip › Li_unmodified_Gels_ED_Fig/Li_unmodified_Gels_ED_Fig4.pdf]

Extended Data Fig. 5

**a**

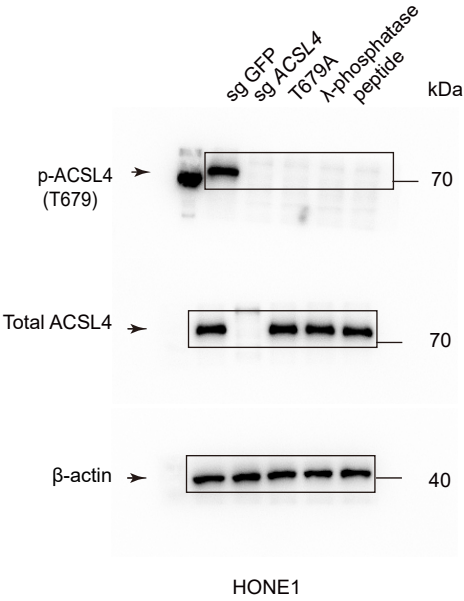

**i**

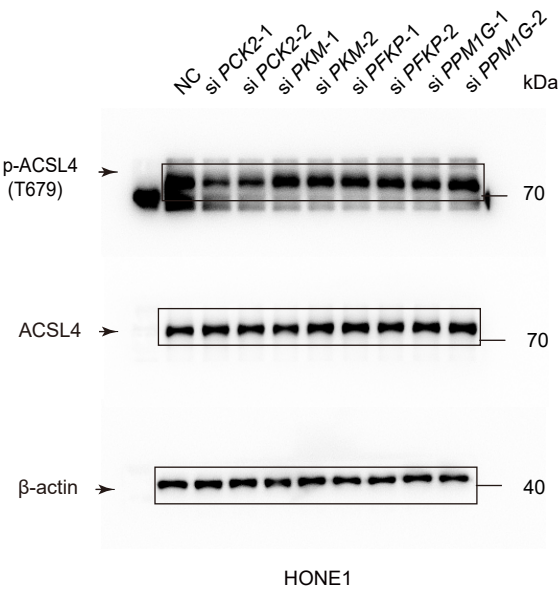

**k**

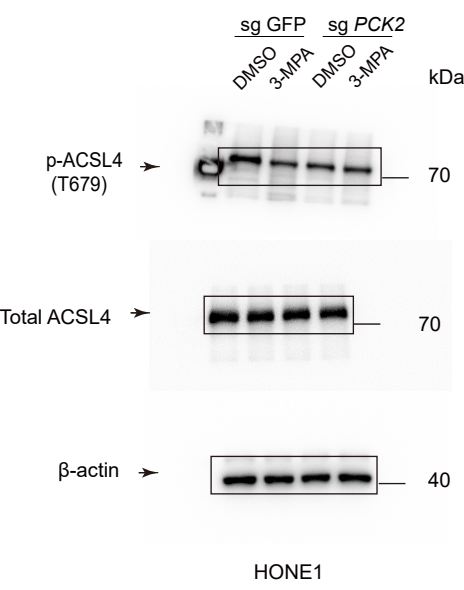

**o**

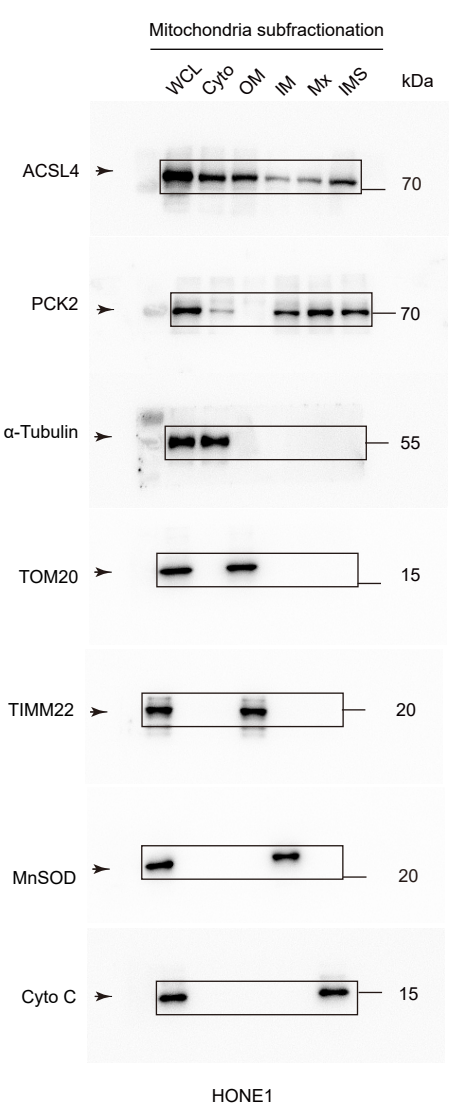

**l**

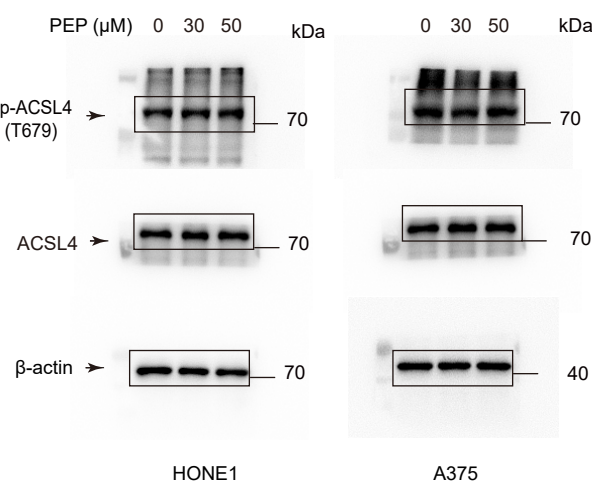

Supplement: Supplementary file 15 — Unprocessed western blots. [file 41589_2024_1612_MOESM15_ESM.zip › Li_unmodified_Gels_ED_Fig/Li_unmodified_Gels_ED_Fig5.pdf]

Extended Data Fig. 6

**a**

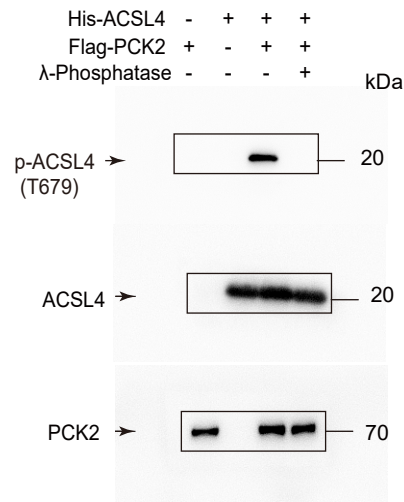

**d**

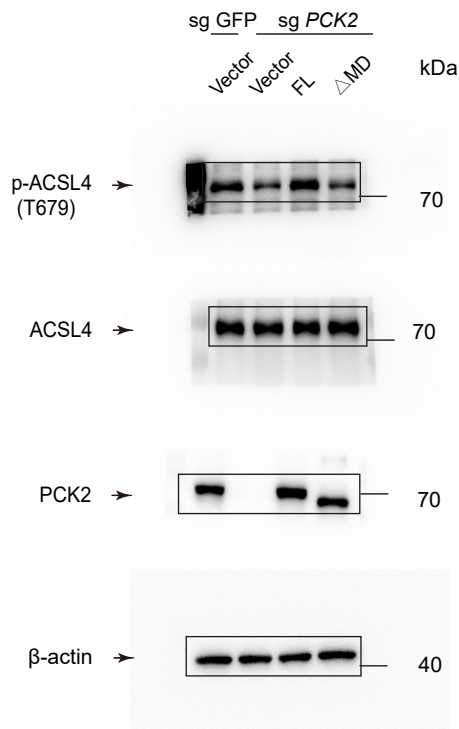

**c**

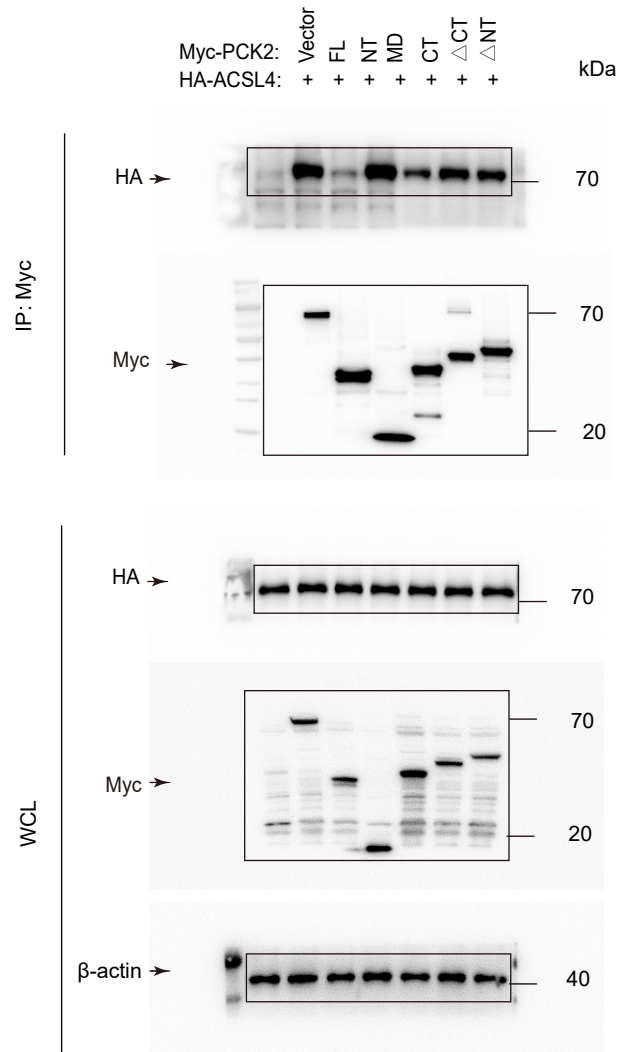

Supplement: Supplementary file 15 — Unprocessed western blots. [file 41589_2024_1612_MOESM15_ESM.zip › Li_unmodified_Gels_ED_Fig/Li_unmodified_Gels_ED_Fig6.pdf]

Extended Data Fig. 7

**c**

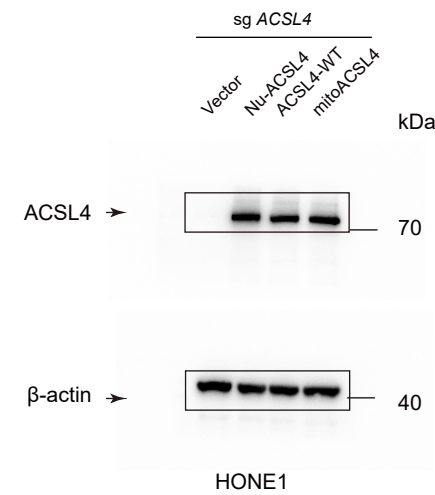

**d**

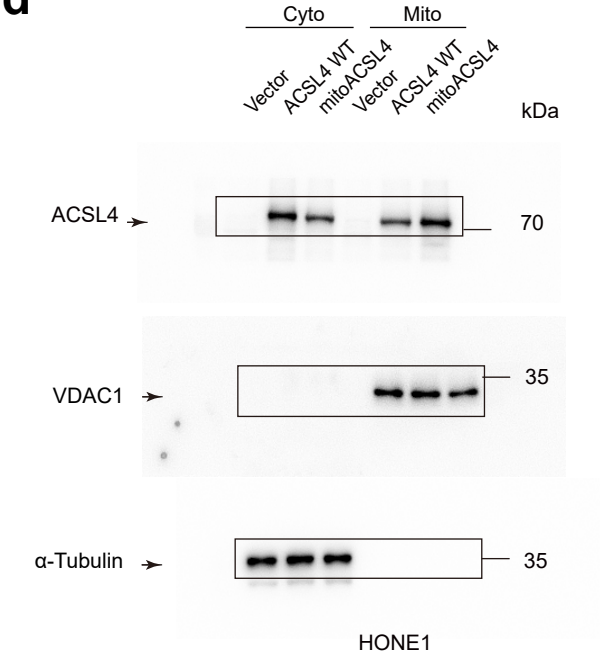

Supplement: Supplementary file 15 — Unprocessed western blots. [file 41589_2024_1612_MOESM15_ESM.zip › Li_unmodified_Gels_ED_Fig/Li_unmodified_Gels_ED_Fig7.pdf]

Extended Data Fig. 8

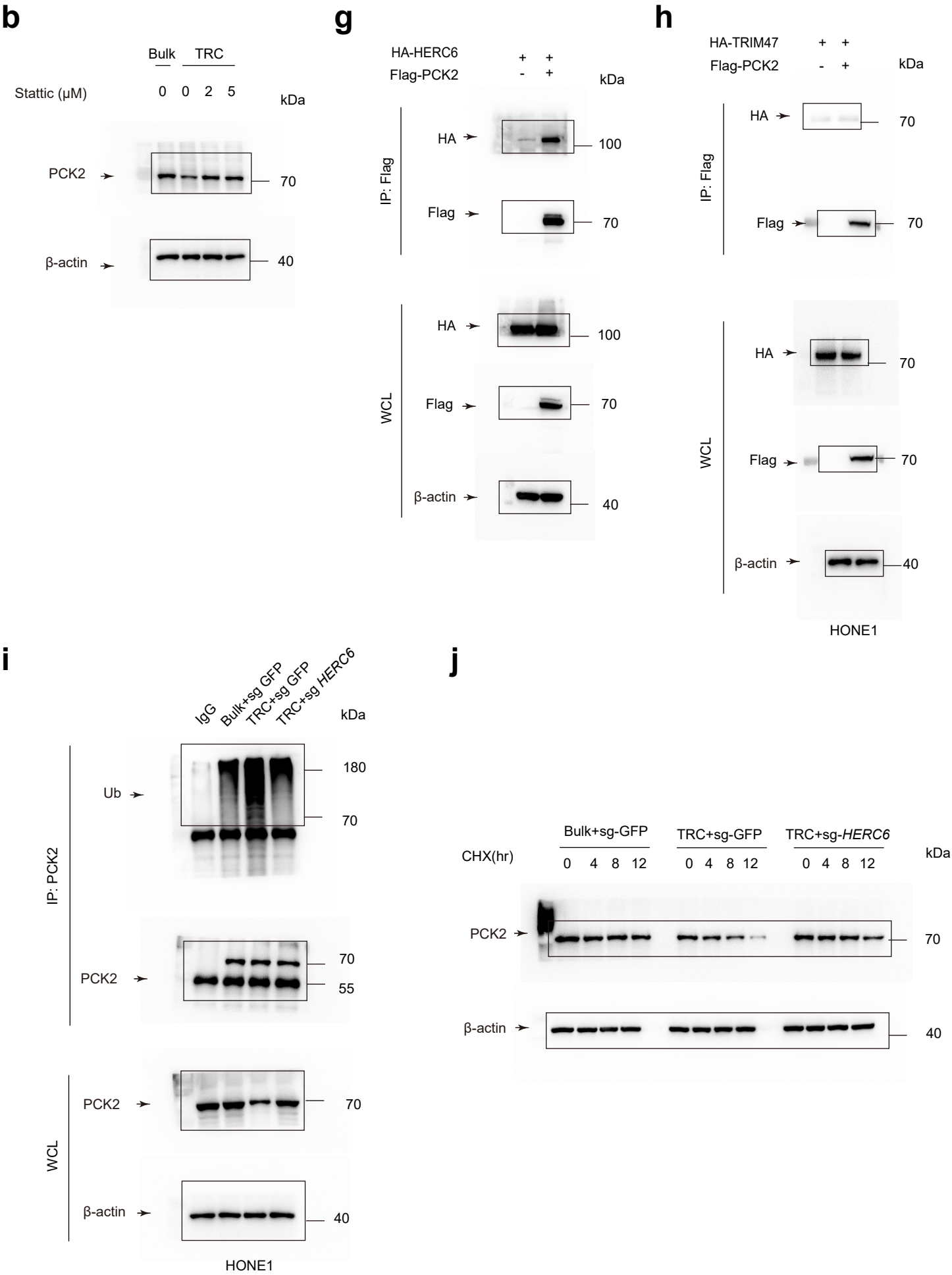

Supplement: Supplementary file 15 — Unprocessed western blots. [file 41589_2024_1612_MOESM15_ESM.zip › Li_unmodified_Gels_ED_Fig/Li_unmodified_Gels_ED_Fig8.pdf]

Extended Data Fig. 9

C

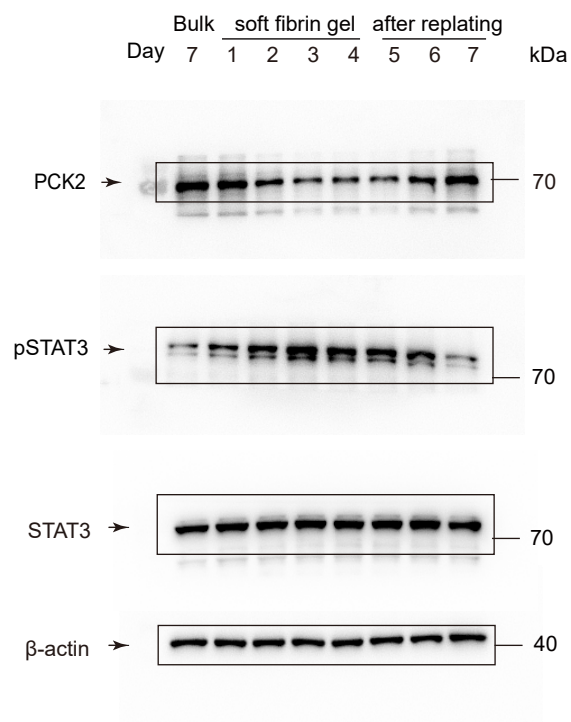

Supplement: Supplementary file 15 — Unprocessed western blots. [file 41589_2024_1612_MOESM15_ESM.zip › Li_unmodified_Gels_ED_Fig/Li_unmodified_Gels_ED_Fig9.pdf]
